# New Rare Triterpene Glycosides from Pacific Sun Star, *Solaster pacificus*, and Their Anticancer Activity

**DOI:** 10.3390/md22010019

**Published:** 2023-12-28

**Authors:** Timofey V. Malyarenko, Alla A. Kicha, Alexandra S. Kuzmich, Olesya S. Malyarenko, Anatoly I. Kalinovsky, Roman S. Popov, Pavel S. Dmitrenok, Natalia V. Ivanchina, Valentin A. Stonik

**Affiliations:** 1 G.B. Elyakov Pacific Institute of Bioorganic Chemistry, Far Eastern Branch, Russian Academy of Sciences, Pr. 100-let Vladivostoku 159, 690022 Vladivostok, Russia; kicha@piboc.dvo.ru (A.A.K.); assavina@mail.ru (A.S.K.); malyarenko.os@gmail.com (O.S.M.); kaaniw@piboc.dvo.ru (A.I.K.); prs_90@mail.ru (R.S.P.); paveldmt@piboc.dvo.ru (P.S.D.); ivanchina@piboc.dvo.ru (N.V.I.); 2 Department of Bioorganic Chemistry and Biotechnology, School of Natural Sciences, Far Eastern Federal University, Russky Island, Ajax Bay, 10, 690922 Vladivostok, Russia

**Keywords:** triterpene glycosides, starfish, *Solaster pacificus*, anticancer activity, HCT 116 cells, MDA-MB-231 cells, colony formation, wound-healing assay

## Abstract

Six previously unknown triterpene glycosides, pacificusosides L–Q (**1**–**6**), and two previously known triterpene glycosides, cucumariosides B_1_ (**7**) and A_5_ (**8**), were isolated from an alcoholic extract of Pacific sun star, *Solaster pacificus*. The structures of **1**–**6** were determined using 1D and 2D NMR, ESIMS, and chemical modifications. Compound **1** is a rare type of triterpene glycoside with non-holostane aglycon, having a linear trisaccharide carbohydrate chain. Pacificusosides M–P (**2**–**5**) have new structures containing a Δ^8(9)^-3,16,18-trihydroxy tetracyclic triterpene moiety. This tetracyclic fragment in sea star or sea cucumber triterpene glycosides was described for the first time. All the compounds under study exhibit low or moderate cytotoxic activity against colorectal carcinoma HCT 116 cells, and breast cancer MDA-MB-231 cells were assessed by MTS assay. Compound **2** effectively suppresses the colony formation of cancer cells at a non-toxic concentration, using the soft-agar assay. A scratch assay has shown a significant anti-invasive potential of compound **2** against HCT 116 cells, but not against MDA-MB-231 cells.

## 1. Introduction

Triterpene glycosides are typical secondary metabolites of sea cucumbers (class Holothuroidea, phylum Echinodermata). These glycosides most commonly have the so-called holostane type of aglycon containing lanostan-3β-ol with (18,20)-lactone in the E-ring of the pentacyclic triterpene core and a carbohydrate chain consisting of five or six monosaccharide residues [1,2]. The terminal monosaccharide residue (D-glucose or D-xylose) in most cases has an additional methoxyl group at C-3, and the carbohydrate chain may contain from one to four sulfate groups. However, rarer triterpene glycosides with a non-holostane type of aglycon [3] or with shorter carbohydrate chains were also found in sea cucumbers. Basically, these secondary metabolites, often exhibiting marked cytotoxic properties, are produced by holothurians to protect themselves against predators [4].

Triterpene glycosides were identified in several starfish species (class Asteroidea, phylum Echinodermata) but their presence in starfish is a rare occurrence. Recently, we have reported about 11 new-to-science triterpene glycosides, pacificusosides A–K, and four previously known triterpene glycosides, cucumariosides A_10_, C_1_, C_2_, and D, isolated from the Pacific sun star *Solaster pacificus* [5,6]. It is likely that some starfish species, being active predators, use sea cucumbers as food and accumulate triterpene glycosides in their bodies. Since each sea cucumber species has its own set of triterpene glycosides, these compounds can be considered as food markers. We previously assumed that starfish can modify sea cucumber triterpene glycosides by their own enzyme systems [5,6]. Thus, we found new triterpene glycosides in *S. pacificus* (Kuril population), presumably specialized in preying on sea cucumbers of the genus *Eupentacta*, which had not previously been reported for this sea cucumber genus [5,6]. This suggests that starfish can modify the most toxic triterpene glycosides by oxidizing and removing part of the aglycon side chain or by reducing the number of monosaccharide residues in the carbohydrate chain of their molecules. This mode of metabolism of triterpene glycosides in *S. pacificus* can be considered as an adaptive mechanism developed for such a specialized diet.

The interest in sea cucumber triterpene glycosides is explained not only by their unusual chemical structure, but also by the diverse biological activities that these compounds exhibit. Thus, antifungal [7,8], bactericidal, hemolytic, antiviral, antiparasitic [9], and immunomodulatory properties [10] of these glycosides were described. Many authors indicate that sea cucumber triterpene glycosides have a significant antitumor potential [11]. For example, frondoside A from *Cucumaria frondosa* inhibits proliferation of AsPC-1 human pancreatic cancer cells by induction of apoptosis of these cells via the mitochondrial pathway and activation of the caspase cascade [12]. Frondoside A decreases the viability of MDA-MB-231 human breast cancer cells in a concentration- and time-dependent manner by activation of p53, followed by emergence of caspases 9 and 3/7 cell death pathways in MDA-MB-231 cells [13]. Furthermore, frondoside A has a potent antimetastatic effect on a syngeneic murine model of metastatic breast cancer [14]. We also showed that pacificusoside C and cucumariosides C_1_ and C_2_ from *S. pacificus* almost completely suppress the colony formation of HT-29, RPMI-7951, and MDA-MB-231 cells at a non-toxic concentration [5]. Moreover, pacificusoside D and cucumarioside D from *S. pacificus* at non-toxic concentrations have the highest inhibiting effect on colony formation of SK-MEL-2 cancer cells, and significantly inhibit neoplastic transformation of JB6 Cl41 cells induced by chemical carcinogens (EGF and TPA) or ionizing radiation (X-rays and UVB) [6].

As a continuation of our research on biologically active low-molecular-weight compounds from the Pacific sun star *S. pacificus* (order Valvatida, family Solasteridae) we herein report the results of studies of six new triterpene glycosides, pacificusosides L–Q (**1**–**6**), and two previously known triterpene glycosides, cucumarioside B_1_ (**7**) and cucumarioside A_5_ (**8**). In this paper, we describe their isolation, structure elucidation, and their effects on the viability, colony formation, and invasion of cancer cells.

## 2. Results and Discussion

### 2.1. Isolation and Structure Elucidation of Compounds ***1**–**8*** from S. pacificus

**Figure 1:**
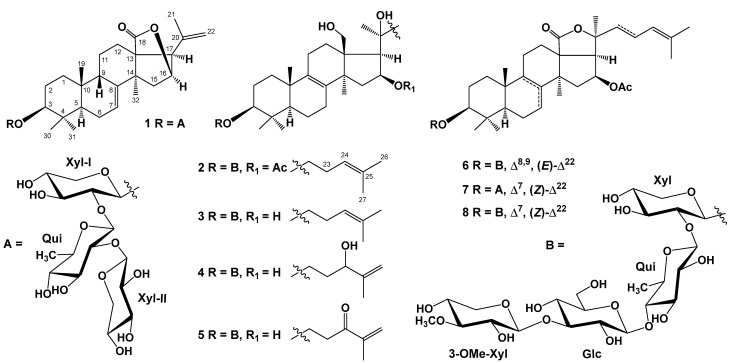
The structures of compounds **1**–**8** isolated from *S. pacificus*.

An ethanol extract from a sun star, *S. pacificus*, was sequentially separated by chromatography on columns with Polychrome 1, Si gel, and Florisil followed by high-performance liquid chromatography (HPLC) on reverse phase Diasfer-110-C18, Discovery C18, and YMC-Pack Pro C18 columns. As a result, six previously unknown triterpene glycosides, referred to as pacificusosides L–Q (**1**–**6**), and two previously known triterpene glycosides (**7** and **8**) were obtained (Figure 1). Compounds **7** and **8** were identified by comparing their ^1^H, ^13^C NMR, and MS spectra to those reported for cucumariosides B_1_ and A_5_ from the sea cucumber *Eupentacta fraudatrix* [1].

**Figure 2:**
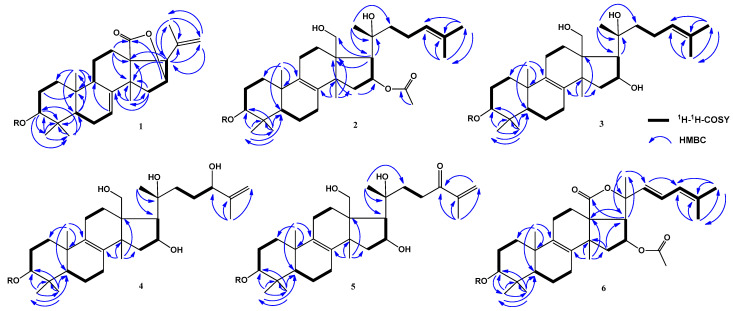
^1^H-^1^H COSY and key HMBC correlations for aglycons of compounds **1**–**6**.

**Figure 3:**
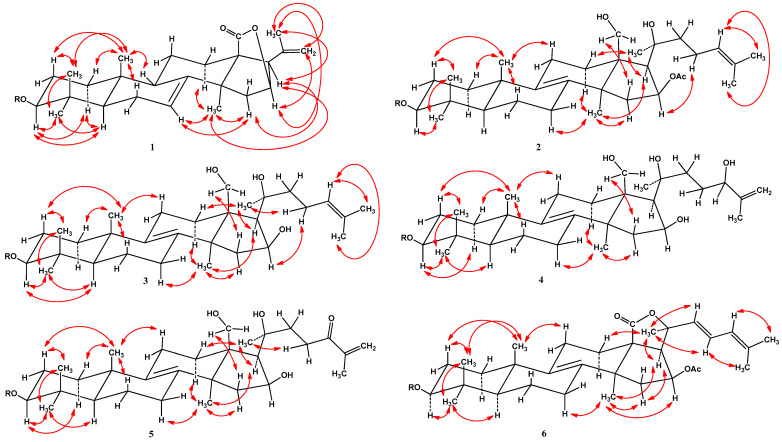
Key ROESY correlations for aglycons of compounds **1**–**6**.

The chemical shifts of protons and carbons of four CH_3_ groups (*δ*_H_ 1.03 s, 1.18 s, 1.32 s, 1.33 s; *δ*_C_ 23.9, 17.3, 28.6, 33.9), the 7(8)-double bond [*δ*_H_ 5.62 brd (*J* = 7.0); *δ*_C_ 122.7, 147.3], and the lactone carbonyl (*δ*_C_ 180.7) were present in the ^1^H and ^13^C NMR spectra of the pentacyclic core of compound **1** (Table 1 and Table 2, Figures S3–S8). The resonances of an acetate group were not observed in the ^1^H and ^13^C NMR spectra of **1**. The respective sequences of protons of polycyclic moiety of **1** shown in Figure 2 were determined by the ^1^H-^1^H COSY and HSQC experiments (Figures S5 and S6). The major HMBC correlations depicted in Figure 2 confirmed the overall structure of the triterpene nucleus of glycoside **1** (Figure S7). The common 5α/9β/10β/13β/14α stereochemistry of the polycyclic moiety and 3β-configuration of oxygenated substituent in **1** were defined from the ROESY cross-peaks (Figure 3 and Figure S8). A CH_3_ group (*δ*_H_ 1.74 s; *δ*_C_ 23.0) and a 20,22-double bond (*δ*_H_ 5.06 s, 4.99 s; *δ*_C_ 139.9, 113.9) were observed in the ^1^H and ^13^C NMR spectra of aglycon side chain of compound **1** (Table 1 and Table 2, Figures S3 and S4). The overall structure of the side chain was supported by major HMBC: H_3_-21/C-17, C-20, C-22 and H-22/C-17, C-21 and ROESY: H_3_-21/H-16, H-17, H-22; and H_2_-22/H-16 correlations (Figure 2, Figure 3, Figures S7 and S8). The NMR spectroscopic data of the aglycon part of glycoside **1** was consistent with those of pacificusosides B and I–K from *S. pacificus* with 23,24,25,26,27-pentanor-lanosta-7,20(22)-diene-18(16)-lactone-3β-ol aglycon [5,6].

Moreover, in the ^1^H NMR spectrum of **1**, three resonances of the anomeric protons of monosaccharide units at *δ*_H_ 4.88, 5.31, and 5.35 were observed, which, in the HSQC experiment, correlated with carbon signals at *δ*_C_ 105.1, 103.4, and 106.4, respectively (Table 3, Figures S3–S8).

**Figure 4:**
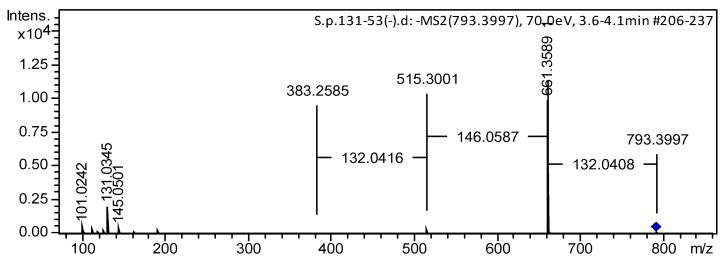
(−)ESIMS/MS spectrum of compound **1**.

ESIMS/MS data confirmed the sequence of monosaccharide units in the carbohydrate chain of glycoside **1**. The (−)ESIMS/MS spectrum of the deprotonated molecule [M − H]^−^ peak at *m*/*z* 793 showed fragmentary peaks obtained through losses of sugar units at *m*/*z* 661 [(M − H)–132]^−^, loss of pentose; at 515 [(M − H)–132–146]^−^, losses of pentose and 6-deoxyhexose; and at 383 [(M − H)–132–146–132]^−^, losses of two pentoses and 6-deoxyhexose (Figure 4 and Figure S9). Accordingly, the (+)ESIMS/MS spectrum of the cationized molecule [M + Na]^+^ at *m*/*z* 817 of **1** showed a series of fragmentary peaks at *m*/*z* 685 [(M + Na)–132]^+^, loss of pentose; at 539 [(M + Na)–132–146]^+^, losses of pentose and 6-deoxyhexose; at 451 [carbohydrate chain + Na]^+^; at 301 [(carbohydrate chain + Na)–132–H_2_O]^+^, losses of pentose and H_2_O from carbohydrate chain; and at 169 [(carbohydrate chain + Na)–2 × 132–H_2_O]^+^, losses of two pentoses and H_2_O from carbohydrate chain (Figure S10).

Along with NMR spectrum information, ESIMS/MS data confirmed the presence of three monosaccharide residues in the carbohydrate chain of glycoside **1**. The ^1^H NMR spectrum of **1** showed a resonance of CH_3_ group of 6-deoxy-sugar unit at *δ*_H_ 1.64. β Configurations of all the glycosidic bonds were determined by the coupling constants of anomeric protons (7.1–7.5 Hz). The chemical shifts of protons and carbons and coupling constants of protons of monosaccharide units in the oligosaccharide moiety of glycoside **1** were identified by the 1D TOCSY, ^1^H-^1^H COSY, HSQC, HMBC, and ROESY experiments (Table 3, Figures S3–S8). The NMR spectroscopic data of the carbohydrate chain exactly coincided with those of the terminal β-d-xylopyranosyl residue and the internal 2-*O*-substituted β-d-quinovopyranosyl and 2-O-substituted β-d-xylopyranosyl residues in the earlier reported ^1^H and ^13^C NMR spectra of the known cucumariosides B_1_ and B_2_ [1]. Cross-peaks between H-1 of Xyl*_p_*-I and C-3 (H-3) of aglycon; H-1 of Qui*_p_* and C-2 (H-2) of Xyl*_p_*-I; and H-1 of Xyl*_p_*-II and C-2 (H-2) of Qui*_p_* in the HMBC and ROESY spectra allowed us to determine the attachment of the oligosaccharide moiety to aglycon and the positions of interglycosidic linkages (Table 3, Figures S7 and S8). The D-configuration for all the monosaccharide units that constitute the carbohydrate chain of **1** was identified in the following way. At the first stage, glycoside **1** was hydrolyzed by 2 M TFA. Next, the resulting mixture of monosaccharides was processed by (*R*)-(−)-octanol, followed by acetylation and GC analysis. Finally, comparison of retention times of the resulting octyl glycoside acetates with the respective derivatives of standard monosaccharides (D-xylose and D-quinovose) showed their almost complete identity (Figures S11–S15). On the basis of these data, the structure of pacificusoside L (**1**) was elucidated as 3β-*O*-[β-d-xylopyranosyl-(1→2)-β-d-quinovopyranosyl-(1→2)-β-d-xylopyranosyl]-23,24,25,26,27-pentanor-5α-lanosta-7,20(22)-diene-18(16)-lactone.

The molecular formula of **2** was identified as C_55_H_90_O_22_ on the basis of the deprotonated molecule peak at *m*/*z* 1101.5857 [M − H]^−^ ([C_55_H_89_O_22_]^−^, 1101.5851) in the (−)HRESIMS spectrum (Figure S16). The IR spectrum of compound **2** showed that hydroxy (3426 cm^−1^) and olefinic (1632 cm^−1^) groups were present (Figure S17). The absorption band of γ-lactone was absent in the IR spectrum of compound **2**, but there was an absorption band of acetate carbonyl (1716 cm^−1^). The chemical shifts of protons and carbons of four CH_3_ groups (*δ*_H_ 1.05 s, 1.11 s, 1.32 s, 1.02 s; *δ*_C_ 19.2, 16.3, 27.7, 26.4), the 8(9)-double bond (*δ*_C_ 132.7, 136.5), an OAc group (*δ*_H_ 2.17 s, *δ*_C_ 21.4, 170.1), and two oxygenated groups CH-3 [*δ*_H_ 3.27 dd (*J* = 12.0, 4.3), *δ*_C_ 88.7] and CH_2_-18 (*δ*_H_ 4.17 m, 3.93 m; *δ*_C_ 62.2) were observed in the ^1^H and ^13^C NMR spectra of the tetracyclic core of compound **2** (Table 1 and Table 2, Figures S18–S23). The respective sequences of protons in the triterpene nucleus from C-1 to C-3, C-5 to C-7, C-11 to C-12, and C-15 to C-17 were determined by the ^1^H-^1^H COSY and HSQC correlations (Figure 2, Figures S20 and S21). The major HMBC cross-peaks: H-15/C-13, C-16; H-17/C-13, C-18, C-20; H_3_-19/C-1, C-5, C-9, C-10; H_3_-30/C-3, C-4, C-5, C-31; H_3_-31/C-3, C-4, C-5, C-30; H_3_-32/C-8, C-13, C-14, C-15; and H_3_C (OAc group)/CO confirmed the overall structure of the tetracyclic triterpene moiety of **2** (Figure 2 and Figure S22). The common 5α/10β/13β/14α stereochemistry of the triterpene nucleus and a 3β,16β-configurations of the oxygenated substituents in **2** were defined from the ROESY cross-peaks (Figure 3 and Figure S23).

Three CH_3_ groups (*δ*_H_ 1.62 s, 2 × 1.67 s; *δ*_C_ 27.6, 17.4, 25.6), the 24(25)-double bond (*δ*_H_ 5.30 m, *δ*_C_ 125.0, 131.1), and a tertiary oxygenated carbon atom (*δ*_C_ 74.6) were observed in the ^1^H and ^13^C NMR spectra of aglycon side chain of compound **2** (Table 1 and Table 2, Figures S18 and S19). The sequence of protons in the aglycon side chain from C-22 to C-27 was determined by the ^1^H-^1^H COSY and HSQC experiments (Table 1 and Table 2, Figures S20 and S21). The major HMBC: H_3_-21/C-17, C-20, C-22; H_3_-26/C-24, C-25, C-27; and H_3_-27/C-24, C-25, C-26, and the ROESY: H_3_-21/H-12, H-17; H-23/H-16; H_3_-26/H-24; and H_3_-27/H-24 correlations confirmed the overall structure of the 20-hydroxy-Δ^24^-lanostane side chain (Figure 1, Figures S22 and S23). The NMR spectroscopic data of the aglycon side chain of **2** were consistent with those of the known cucumarioside A_8_ from *E. fraudatrix*, which contains a non-holostane-type aglycon and a 20-hydroxy-Δ^24^-cholestane side chain [15]. Moreover, an (*S*)-configuration of the C-20 asymmetric center was determined, based on the ROESY cross-peaks H_3_-21/H_β_-12 and H-23/H-16 [15].

Moreover, in the ^1^H NMR spectrum of **2**, four resonances of the anomeric protons of monosaccharide units at *δ*_H_ 4.79, 5.16, 4.97, and 5.20 were observed, which, in the HSQC experiment, correlated with carbon signals at *δ*_C_ 105.5, 105.5, 104.9, and 106.0, respectively, together with the signal of *O*-CH_3_ at *δ*_H_ 3.85, which, in the HSQC experiment, was correlated with a carbon signal at *δ*_C_ 60.5 (Table 3, Figures S18–S23).

**Figure 5:**
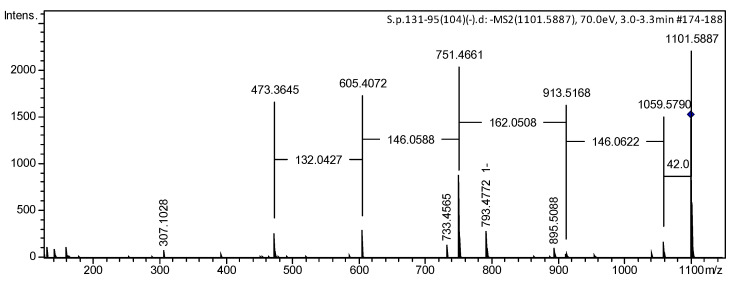
(−)ESIMS/MS spectrum of compound **2**.

ESIMS/MS data confirmed the sequence of monosaccharide units in the carbohydrate chain of glycoside **2**. The (−)ESIMS/MS spectrum of the deprotonated molecule [M − H]^−^ peak at *m*/*z* 1101 showed fragmentary peaks obtained due to the losses of sugar units at *m*/*z* 913 [(M − H)−146−42]^−^, losses of *O*-Me-pentose and Ac-group; at 751 [(M − H)–146–162–42]^−^, losses of *O*-Me-pentose, hexose and Ac-group; at 605 [(M − H)−146−162−146−42]^−^, losses of *O*-Me-pentose, hexose, 6-deoxyhexose, and Ac-group; and at 473 [(M − H)–146–162–146–132–42]^−^, losses of *O*-Me-pentose, hexose, 6-deoxyhexose, pentose and Ac-group (Figure 5 and Figure S24). The (+)ESIMS/MS spectrum of the peak of the cationized molecule [M + Na]^+^ at *m*/*z* 1125 of **2** showed a series of fragmentary peaks at *m*/*z* 1065 [(M + Na)–60]^+^, loss of OAc group; at 627 [carbohydrate chain + Na]^+^ at 477 [(carbohydrate chain + Na)–132–H_2_O]^+^, losses of pentose and H_2_O from carbohydrate chain; at 331 [(carbohydrate chain + Na)–132–146–H_2_O]^+^, losses of pentose, *O*-Me-pentose, and H_2_O from the carbohydrate chain, or losses of pentose, 6-deoxyhexose, and H_2_O from the carbohydrate chain; and at 185 [(carbohydrate chain + Na)–132–146–146–H_2_O]^+^, losses of pentose, *O*-Me-pentose, 6-deoxyhexose, and H_2_O from the carbohydrate chain (Figure S25).

Along with NMR spectrum information, ESIMS/MS data confirmed the presence of four monosaccharide residues in the carbohydrate chain of glycoside **2**. In the ^1^H NMR spectrum of **2**, a resonance of CH_3_ group of 6-deoxy-sugar unit at *δ*_H_ 1.76 was observed. β-Configurations of all the glycosidic bonds were determined by the coupling constants of anomeric protons (7.5–8.0 Hz). The chemical shifts of protons and carbons and coupling constants of protons of monosaccharide units in the oligosaccharide moiety of glycoside **2** were identified by the 1D TOCSY, ^1^H-^1^H COSY, HSQC, HMBC, and ROESY experiments (Table 3, Figures S18–S23).

The NMR spectroscopic data of the carbohydrate chain exactly coincided with those of the terminal 3-*O*-Me-β-xylopyranosyl residue and the internal 3-*O*-substituted β-glucopyranosyl, 4-*O*-substituted β-quinovopyranosyl, and 2-*O*-substituted β-xylopyranosyl residues in the earlier reported ^1^H and ^13^C NMR spectra of the known cucumarioside A_5_ [1]. Cross-peaks between H-1 of Xyl*_p_* and C-3 (H-3) of aglycon; H-1 of Qui*_p_* and C-2 (H-2) of Xyl*_p_*; H-1 of Glc*_p_* and C-4 (H-4) of Qui*_p_*; H-1 of 3-*O*-Me-Xyl*_p_* and C-3 (H-3) of Glc*_p_* in the HMBC and ROESY spectra allowed us to determine the attachment of the oligosaccharide moiety to aglycon and the positions of interglycosidic linkages (Table 3, Figures S22 and S23). The D-series of monosaccharide units was expected to be similar to that in co-occurring glycoside **1**.

Accordingly, the structure of pacificusoside M (**2**) was identified as (20*S*)-3β-*O*-[3-*O*-methyl-β-d-xylopyranosyl-(1→3)-β-d-glucopyranosyl-(1→4)-β-d-quinovopyranosyl-(1→2)-β-d-xylopyranosyl]-16β-acetoxy,18,20-dihydroxy-5α-lanosta-8(9),24-diene.

A comparison of the ^1^H, ^13^C NMR and MS spectra and an application of extensive 2D NMR analysis of compounds **2**–**6** showed that the oligosaccharide moiety of **2** is identical to that in compounds **3**–**6** (Figures S33, S34, S43, S44, S52 and S53), while compounds **2**–**6** differ from each other in triterpene aglycons only (Table 1, Table 2 and Table 3).

The molecular formula of **3** was identified as C_53_H_88_O_21_, on the basis of the cationized molecule peak at *m*/*z* 1083.5693 [M + Na]^+^ (calculated for [C_53_H_88_O_21_Na]^+^, 1083.5710) in the (+)HRESIMS spectrum (Figure S26). A comparison of the molecular weights (MWs) of **3** and **2** showed that the difference between **3** and **2** was 42 atomic mass units (amu’s). Most of the signals in the NMR spectra of **3** attributable to triterpene nucleus were similar to those of **2**, except some resonances belonging to D-ring. The signals of H-15 (m), H-16 (m), and H-17 (m) in **3** were upfield-shifted from *δ*_H_ 2.26 to 2.07, from *δ*_H_ 5.85 to 5.02, and from *δ*_H_ 2.32 to 2.15, respectively, compared to those of **2**. Also, in the ^1^H NMR spectrum of **3**, there was no resonance of acetate group at *δ*_H_ 2.17 s (CH_3_CO). In the ^13^C NMR spectrum of **3**, the signal of C-16 was upfield-shifted from *δ*_C_ 76.6 to 73.5, and resonances of the acetate group at *δ*_C_ 21.4 (CH_3_CO) and 170.1 (CH_3_CO) were absent, compared to those of **2**. Thus, these data indicated the absence of an acetate group at C-16 in **3**. The ^1^H-^1^H COSY, HSQC, HMBC, and ROESY experiments allowed us to determine all proton and carbon resonances of glycoside **3** (Table 1, Table 2 and Table 3; Figure 2, Figure 3 and Figures S27–S32). Accordingly, the structure of pacificusoside N (**3**) was identified as (20*S*)-3β-*O*-[3-*O*-methyl-β-d-xylopyranosyl-(1→3)-β-d-glucopyranosyl-(1→4)-β-d-quinovopyranosyl-(1→2)-β-d-xylopyranosyl]-16β,18,20-trihydroxy-5α-lanosta-8(9),24-diene.

Compounds **4** and **5** were not separated by repeated reversed-phase HPLC. Compound **4** was characterized from a mixture with compound **5** (2:1, *v*/*v*) by estimating ion peak intensities in the ESI mass-spectra. (+)-HRESIMS of this mixture demonstrated two [M+Na]^+^ ion peaks at *m*/*z* 1099.5624 corresponding to **4**, and at *m*/*z* 1097.5501 corresponding to **5**. Accordingly, the molecular formula of **4** was identified as C_53_H_88_O_22_ from the deprotonated molecule peak at *m*/*z* 1075.5664 [M–H]^−^ (calculated for [C_53_H_87_O_22_]^−^, 1075.5694) in the (−)HRESIMS. The molecular formula of **5** was identified as C_53_H_86_O_22_ from the deprotonated molecule peak at *m*/*z* 1073.5542 [M–H]^−^ (calculated for [C_53_H_85_O_22_]^−^, 1073.5538) in the (−)HRESIMS (Figure S35). Thus, the MWs of **4** and **5** differed by 2 amu’s. The IR spectrum of the mixture of **4** and **5** demonstrated the presence of only hydroxy (3439 cm^−1^) and olefinic (1632 cm^−1^) groups (Figure S36). It was found that compounds **4**, **5**, and **3** differed from each other only in signals of their side chains, on the basis of a thorough comparison of their ^1^H and ^13^C NMR data (Table 1, Table 2 and Table 3, Figures S37 and S38).

Two CH_3_ groups (*δ*_H_ 1.67 s, 1.93 s; *δ*_C_ 26.2, 17.5), a tertiary hydroxyl group (*δ*_C_ 76.6), a secondary hydroxyl group [*δ*_H_ 4.47 brd (*J* = 9.2); *δ*_C_ 75.8], and a terminal double bond (*δ*_H_ 5.26 brs, 4.94 brs; *δ*_C_ 148.7, 110.0) were observed in the ^1^H and ^13^C NMR spectra of aglycon side chain of compound **4**. The sequence of protons from H-22 to H-24, which correlated with the respective carbon atoms in the side chain of **4**, was attributed using the COSY and HSQC experiments (Table 1 and Table 2, Figures S39 and S40). The major HMBC correlations depicted in Figure 2 confirmed the overall structure of the (20*S*)-Δ^25^-20,24-dihydroxy-lanostane side chain (Figure 2 and Figure S41). Thus, the aglycon side chain of glycoside **4** was identical to that of the known cucumarioside A_9_ [1]. Therefore, the structure of pacificusoside O (**4**) was identified as (20*S*)-3β-*O*-[3-*O*-methyl-β-d-xylopyranosyl-(1→3)-β-d-glucopyranosyl-(1→4)-β-d-quinovopyranosyl-(1→2)-β-d-xylopyranosyl]-16β,18,20,24ξ-tetrahydroxy-5α-lanosta-8(9),25-diene.

Two CH_3_ groups (*δ*_H_ 1.60 s, 1.94 s; *δ*_C_ 25.5, 17.7), a tertiary hydroxyl group (*δ*_C_ 76.1), and a carbonyl group (*δ*_C_ 201.9) conjugated with the terminal double bond (*δ*_H_ 6.08 brs, 5.67 brs; *δ*_C_ 144.4, 124.2) were observed in the ^1^H and ^13^C NMR spectra of aglycon side chain of compound **5**. The sequences of protons in the aglycon side chain from C-22 to C-23 were determined by the ^1^H-^1^H COSY and HSQC experiments (Table 1 and Table 2, Figures S39 and S40). The major HMBC and ROESY cross-peaks supported the overall structure of the (20*S*)-Δ^25^-20-hydroxy-24-oxo-lanostane side chain (Figure 2, Figures S41 and S42). Thus, the structure of pacificusoside P (**5**) was identified as (20*S*)-3β-*O*-[3-*O*-methyl-β-d-xylopyranosyl-(1→3)-β-d-glucopyranosyl-(1→4)-β-d-quinovopyranosyl-(1→2)-β-d-xylopyranosyl]-16β,18,20-trihydroxy-5α-lanosta-8(9),25-diene-24-one.

The molecular formula of **6** was identified as C_55_H_84_O_22_ from the deprotonated molecule peak at *m*/*z* 1095.5381 [M − H]^−^ (calculated for [C_55_H_83_O_22_]^−^, 1095.5381) in the (−)HRESIMS spectrum (Figure S45). The chemical shifts of protons and carbons of four CH_3_ groups (*δ*_H_ 1.31 s, 1.15 s, 1.34 s, 1.07 s; *δ*_C_ 19.0, 16.3, 27.1, 26.5), the 8(9)-double bond (*δ*_C_ 134.2, 136.0), an OAc group (*δ*_H_ 2.06 s, *δ*_C_ 21.0, 170.5), a lactone carbonyl (*δ*_C_ 176.5), and one oxygenated group CH-3 [*δ*_H_ 3.32 dd (*J* = 11.5, 3.9), *δ*_C_ 88.8] were present in the ^1^H and ^13^C NMR spectra of the pentacyclic core of compound **6** (Table 1 and Table 2, Figures S46 and S47). The respective sequences of protons of polycyclic moiety of **6** shown in Figure 2 were determined by the ^1^H-^1^H COSY and HSQC experiments (Figures S48 and S49). The major HMBC cross-peaks: H-3/C-30; H-15/C-14, C-17; H-17/C-13, C-18, C-20; H_3_-19/C-1, C-5, C-9, C-10; H_3_-30/C-3, C-4, C-5, C-31; H_3_-31/C-3, C-4, C-5, C-30; and H_3_-32/C-8, C-13, C-14, C-15 confirmed the overall structure of the triterpene nucleus of glycoside **6** (Figure 2 and Figure S50). The common 5α/9β/10β/13β/14α stereochemistry of the polycyclic moiety and 3β-configuration of oxygenated substituent in **6** were defined the ROESY cross-peaks (Figure 3 and Figure S51).

Three CH_3_ groups (*δ*_H_ 1.56 s, 1.64 s, 1.72 s; *δ*_C_ 30.5, 18.2, 25.6) and the conjugated 22,24-diene system (*δ*_H_ 5.93 d (*J* = 15.8), 6.58 dd (*J* = 15.8, 11.3), 5.90 d (11.3); *δ*_C_ 134.2, 122.2, 125.3, 134.5) were observed in the ^1^H and ^13^C NMR spectra of aglycon side chain of compound **6** (Table 1 and Table 2, Figures S46 and S47). The sequences of protons in the aglycon side chain from C-22 to C-27 were determined by the ^1^H-^1^H COSY and HSQC experiments (Table 1 and Table 2, Figures S48 and S49). The overall structure of the Δ^22,24^-lanostane side chain was supported by major HMBC: H_3_-21/C-17, C-20, C-22; H-22/C-24; H_3_-26/C-24, C-25, C-27; H_3_-27/C-24, C-25, C-26 and ROESY: H_3_-21/H-17, H-22, H-23; H-23/H_3_-27; H-24/H_3_-26 correlations (Figure 1, Figures S50 and S51). Coupling constant value at 15.8 Hz confirmed the *trans* configuration of the 22(23)-double bond. The NMR spectroscopic data of the aglycon side chain of **6** were consistent with those of pacificusoside D with the same side chain, previously isolated from *S. pacificus* [6].

Therefore, the structure of pacificusoside Q (**6**) was identified as 3β-*O*-[3-*O*-methyl-β-d-xylopyranosyl-(1→3)-β-d-glucopyranosyl-(1→4)-β-d-quinovopyranosyl-(1→2)-β-d-xylopyranosyl]-16β-acetoxyholosta-8(9),22*E*,24-triene.

Isolation of a series of new triterpene glycosides from a starfish species is a rare case. In this study, we isolated a series of eight compounds: six previously unknown triterpene glycosides, pacificusosides L–Q (**1**–**6**), and two previously known triterpene glycosides, cucumariosides B_1_ (**7**) and A_5_ (**8**), from the sun star *S. pacificus.* Thus, the total number of triterpene glycosides isolated from this species reached 23. Earlier, we reported that the isolated compounds have a clear structural similarity with the triterpene glycosides isolated from the sea cucumber *E. fraudatrix* [5,6]. Indeed, pacificusoside L (**1**) has the same β-d-xylopyranosyl-(1→2)-β-d-quinovopyranosyl-(1→2)-β-d-xylopyranosyl carbohydrate chain as cucumariosides B_1_ and B_2_ from *E. fraudatrix*, but differs from these compounds by a rare type of non-holostane aglycon [1]. Pacificusosides M–Q (**2**–**6**) contain a 3-*O*-methyl-β-d-xylopyranosyl-(1→3)-β-d-glucopyranosyl-(1→4)-β-d-quinovopyranosyl-(1→2)-β-d-xylopyranosyl oligoglycoside chain and non-oxidized (Δ^24^-lanostane and Δ^22,24^-lanostane) and oxidized (24-hydroxy-Δ^25^-lanostane and 24-oxo-Δ^25^-lanostane) aglycon side chains that are similar to the A-series cucumariosides from the same sea cucumber species [1]. However, the triterpene glycosides from *S. pacificus* show a significant structural difference. It should be noted that all triterpene glycosides from *E. fraudatrix* belong to a series of Δ^7^ glycosides with a holostane or non-holostane type of aglycon [1], while pacificusosides M–Q (**2**–**6**) have a Δ^8(9)^-double bond in the triterpene aglycon. Triterpene glycosides with the Δ^8(9)^-double bond in the aglycon are extremely rare in sea cucumbers [1]. As far as we know, only 10 such compounds were found to date: psolusoside B_1_ from *Psolus fabricii* [2], synaptoside A_1_ from *Synapta maculata* [1], variegatusides B and D from *Stichopus variegatus* [16,17], having a 3β-hydroxyholost-8(9)-ene skeleton, and fallaxosides C_1_, C_2_, D_1_, D_2_, D_4_, and D_5_ from *Cucumaria fallax* [1], with the non-holostane type of triterpene aglycon. The five new pacificusosides M–Q (**2**–**6**) with the Δ^8(9)^-double bond in aglycon have significantly extended the list of these rare compounds.

We previously suggested that the unusual triterpene glycosides in *S. pacificus* could be produced by the biosynthetic enzyme systems from related dietary glycosides [5,6]. Such modifications can occur either through oxidation followed by degradation of the aglycon side chain [5] or through reduction in the number of monosaccharide units in the oligosaccharide chain of triterpene glycosides [6]. It is confirmed, in part, by a significant decrease in the toxicity of the modified compounds, and can be considered as an adaptation mechanism of starfish specialized in preying on sea cucumbers. The unusual pacificusosides M–Q (**2**–**6**) have much in common with the A-series cucumariosides from *E. fraudatrix* [1]. Thus, pacificusoside M is almost identical to cucumarioside A_8_, and pacificusoside O is similar to cucumarioside A_9_, except for the position of the double bond in the triterpene aglycon: Δ^8(9)^ in pacificusosides M and O and Δ^7^ in cucumariosides A_8_ and A_9_. This fact allows the assumption that pacificusosides M–Q (**2**–**6**) are also products of modification of the starfish biosynthetic enzyme systems and are derived by isomerization of the double bond 7(8) to 8(9). Thus, the isomerization of the double bond in the triterpene aglycon may be another way to utilize toxic triterpene glycosides taken up with food by the sun star, *S. pacificus*.

### 2.2. Investigation of Biological Activities

#### 2.2.1. Cytotoxicity of Compounds **1**–**3** and **6**–**8** against Normal and Cancer Cells

Cancer is known to be a complex process characterized by mutation and selection for cells with progressively increasing capacity for proliferation, survival, invasion, and metastasis [18,19,20]. In the present study, the effect of compounds **1**–**3** and **6**–**8** on important hallmarks of cancer such as viability, proliferation, and invasion of cancer cells was investigated.

We determined the cytotoxicity of compounds **1**–**3** and **6**–**8** against human embryonic kidney HEK 293, colorectal carcinoma HCT 116, and breast cancer MDA-MB-231 cells by the MTS assay, after 24 h of treatment with the compounds. We calculated the inhibiting concentration that cause death of 50% of cells (IC_50_) and the selectivity index (SI) of the compounds tested (Table 4).

Compounds **1**, **6**, **7**, and **8** proved to be non-selective against cancer cells and exhibited cytotoxic activity against HEK 293, HCT 116, and MDA-MB-231 cells, to a varying degree (Table 4). Moderate cytotoxic effect of compounds **2** and **3** was observed against normal HEK 293 cells and cancer HCT 116 or MDA-MB-231 cells. IC_50_ of compound **2** was estimated at 18.6 µM against HCT 116 cells (SI = 1.0) and 15.5 µM against MDA-MB-231 cells (SI = 1.2). Compound **3** possessed less cytotoxic activity than compound **2**, with its IC_50_ value of 42.2 µM against HCT 116 cells (SI = 1.25) and 36.7 µM against MDA-MB-231 cells (SI = 1.4) (Table 4). Since compound **2** exhibited high cytotoxic activities against the tested cell lines, it was chosen for the further study of its colony-inhibiting and anti-invasive effects at low non-toxic concentrations of 0.6, 1.25, 2.5, and 5 µM. The chemotherapeutic drug, cisplatin, was used as positive control. IC_50_ of cisplatin against HEK 293, HCT 116, and MDA-MB-231 cells was determined to be 64.6 µM, 40.2.9 µM, and 34.5 µM, respectively, after 48 h of cell incubation (Table 4).

#### 2.2.2. Colony-Inhibiting Activity of Compound **2** in Cancer Cells HCT 116 and MDA-MB-231

**Figure 6:**
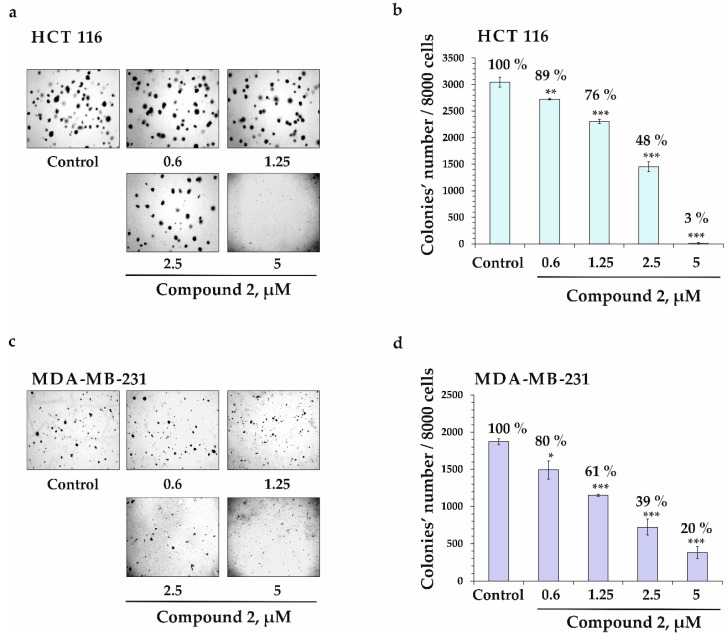
The inhibiting effect of compound **2** on the colony formation activity of human colorectal carcinoma (HCT 116) and breast cancer (MDA-MB-231) cells. (**a**,**b**) HCT 116 or (**c**,**d**) MDA-MB-231 cells were treated with **2** (at 0.6, 1.25, 2.5, and 5 µM) on soft agar. Number of colonies was counted under a microscope (at a total magnification of 40×) using the ImageJ v. 1.50i software, bundled with 64-bit Java 1.6.0_24. Results are presented as mean ± standard deviation (SD). The one-way ANOVA and Tukey’s HSD test for multiple comparisons showed statistical significance: * *p* < 0.05, ** *p* < 0.01, and *** *p* < 0.001.

The soft-agar clonogenic assay was applied in order to assess the effect of compound **2** on the colony formation of human colorectal carcinoma HCT 116 and breast cancer MDA-MB-231 cells. As a result, compound **2** at concentrations of 0.6, 1.25, 2.5, and 5 µM decreased the number of colonies of HCT 116 cells by 11, 24, 52, and 97%, respectively (Figure 6a,b) and MDA-MB-231 cells by 20, 39, 61, and 80%, respectively (Figure 6c,d).

#### 2.2.3. Anti-Invasive Activity of Compound **2** in Cancer Cells HCT 116 and MDA-MB-231

**Figure 7:**
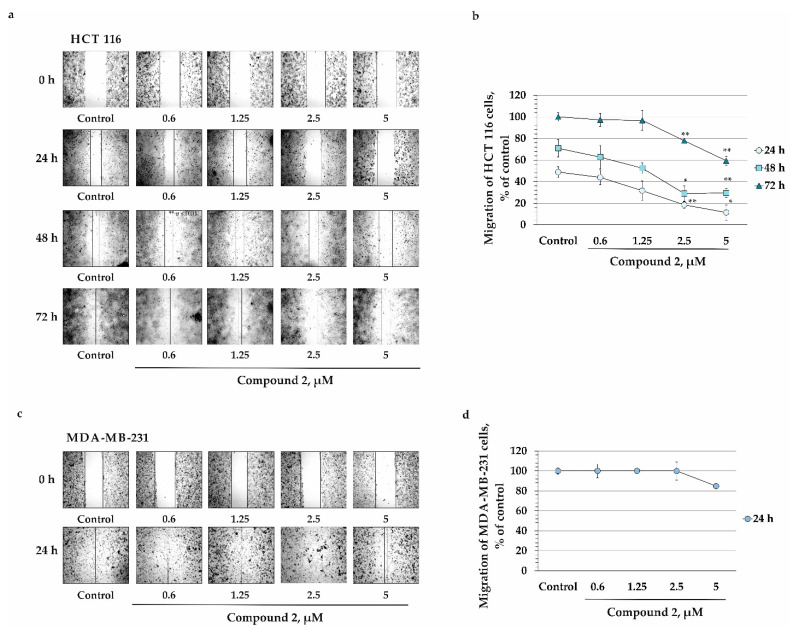
The effect of compound **2** on migration of human colorectal carcinoma HCT 116 cells and breast cancer MDA-MB-231 cells. (**a**,**b**) HCT 116 and (**c**,**d**) MDA-MB-231 cells were treated with **2** (0.6, 1.25, 2.5, 5 µM) for 24, 48, and 72 h. The cell migration distance was estimated by measuring the width of the wound, and was expressed as the percentage of each control in relation to the mean of the wound-closure area. All experiments were set up at least in triplicate (*n* = 9 for control and compound; *n* is the number of photographs). The magnification of the representative photos is ×10. The results are expressed as mean ± standard deviation (SD). The asterisks (* *p* < 0.05, ** *p* < 0.01) indicate a significant decrease in migration of cells treated with the compound, compared to control.

Metastasis is the leading cause of cancer mortality [21]. The metastatic cascade is a multistep process in which cancer cells are destroyed, from the primary tumor to distant parts and tissues [22]. Migration and plasticity of cancer cells, as well as the environment such as stromal and endothelial cells, are essential in metastasis [23]. In this study, the migration ability (invasive potential) of colorectal carcinoma HCT 116 cells and breast cancer MDA-MB-231 cells and the anti-invasive effect of compound **2** were determined by the “wound-healing”, or scratch, method. HCT 116 cells were found to migrate by 49, 71, and 100% slower after 24, 48, and 72 h of cell incubation, respectively, compared to control at the time point of 0 h (Figure 7a). MDA-MB-231 cells showed a higher migration speed than HCT 116 cells, and were able to completely heal the “experimental wound” after 24 h of incubation, compared to control at 0 h (Figure 7c).

We found that compound **2** at concentrations of 0.6, 1.25, 2.5, and 5 µM inhibited the migration of HCT 116 cells by 5, 17, 30, and 37% after 24 h of treatment, respectively, compared to non-treated cells (control, 24 h). A treatment of HCT 116 cells with compound **2** (0.6, 1.25, 2.5, and 5 µM) for 48 h led to the suppression of cells’ migration by 8, 18, 42, and 41%, respectively, compared to control at 48 h. Also, compound **2** reduced the migration of HCT 116 cells by 3, 3, 23, and 41% after 72 h of treatment, respectively, compared to control at 72 h (Figure 7a,b). On the other hand, compound **2** slightly influenced the migration of MDA-MB-231 cells, and at 5 µM inhibited cells’ migration only by 15% after 24 h of treatment, compared to control at 24 h (Figure 7c,d).

Thus, we assessed the cytotoxicity of compounds **1**–**3** and **6**–**8** against human embryonic kidney HEK 293, colorectal carcinoma HCT 116, and breast cancer MDA-MB-231 cells. All the compounds under study exhibited low or moderate cytotoxic activity against the cell lines tested. Unfortunately, we could not find any selectivity of the effect of these triterpene glycosides towards cancer cells. Nevertheless, compound **2** effectively suppressed the colony formation activity of HCT 116 and MDA-MB-231 cells at non-toxic concentrations in a dose-dependent manner, and inhibited migration of HCT 116 cells after 24, 48, and 72 h of treatment. This suggests that even highly toxic sea cucumber (or starfish) triterpene glycosides can be considered promising antitumor agents. Further investigations into the molecular mechanism of the anticancer effect of these compounds are expected to provide sufficient scientific evidence for research and applied purposes.

## 3. Materials and Methods

### 3.1. General Methods

Optical rotations were determined on a PerkinElmer 343 polarimeter (Waltham, MA, USA). IR spectra were recorded using an Equinox 55 spectrophotometer (Bruker, Bremen, Germany). ^1^H and ^13^C NMR spectra were recorded on a Bruker Avance III 700 spectrometer (Bruker BioSpin, Bremen, Germany) at 700.13 and 176.04 MHz, respectively, with chemical shifts referenced to the respective residual solvent signal (*δ*_H_ 7.21/*δ*_C_ 123.5 for C_5_D_5_N). The HRESIMS spectra were recorded on a Bruker Impact II Q-TOF mass spectrometer (Bruker, Bremen, Germany); the samples were dissolved in MeOH (at 0.001 mg/mL). HPLC separations were carried out on an Agilent 1100 Series chromatograph (Agilent Technologies, Santa Clara, CA, USA) equipped with a differential refractometer; the columns used were as follows: Diasfer-110-C18 (10 µm, 250 × 15 mm, Biochemmack, Moscow, Russia), Discovery C_18_ (5 µm, 250 × 4 mm, Supelco, North Harrison, PA, USA), and YMC-Pack Pro C18 (5 μm, 250 × 4.6 mm, YMC Co., Ltd., Kyoto, Japan). Low-pressure liquid column chromatography was carried out on Polychrome 1 (powdered Teflon, 0.25−0.50 mm; Biolar, Olaine, Latvia), Florisil (60–100 µm, Sigma-Aldrich Co., St. Louis, MO, USA), and Si gel KSK (50–160 µm, Sorbpolimer, Krasnodar, Russia) columns. Sorbfil Si gel plates (4.5 × 6.0 cm, 5–17 µm, Sorbpolimer, Krasnodar, Russia) were used for thin-layer chromatography (TLC).

### 3.2. Animal Material

Specimens of *S. pacificus* were collected as described previously [5,6], near Iturup Island in the Sea of Okhotsk, at a depth of 10–20 m during the 42nd scientific cruise of R/V *Akademik Oparin* in August 2012. A voucher specimen (no. 042-112) was deposited at the marine specimen collection of the G.B. Elyakov Pacific Institute of Bioorganic Chemistry FEB RAS, Vladivostok, Russia.

### 3.3. Extraction and Isolation

Extraction and low-pressure chromatography on Polychrome 1 and Si gel columns were completed as described previously [5,6]. Fraction 3 (1.36 g) obtained after Si gel column chromatography was additionally chromatographed on a Florisil column (4 × 10 cm) using CHCl_3_/EtOH (stepwise gradient, 8:1 to 4:1, *v*/*v*) to yield two fractions, 31 (542 mg) and 32 (629 mg). The HPLC separation of fractions 31 and 32, performed on a Diasfer-110-C18 column (10 μm, 250 × 15 mm, 2.5 mL/min) with EtOH/H_2_O (60:40) as an eluent system, followed by separation on a Discovery C18 column (5 μm, 250 × 10 mm, 2.5 mL/min) with EtOH/H_2_O (55:45, *v*/*v*) as an eluent system, yielded pure **1** (5.5 mg, *t*_R_ 29.2 min), **2** (5.0 mg, *t*_R_ 34.4 min), **3** (1.5 mg, *t*_R_ 48.3 min), a mixture of **4** and **5** (0.7 mg, *t*_R_ 12.7 min), **7** (0.5 mg, *t*_R_ 36.5 min), **8** (46.8 mg, *t*_R_ 45.6 min), and a subfraction 32-10-5 (3.5 mg). The HPLC separation of subfraction 32-10-5 on a YMC-Pack Pro C18 column (5 μm, 250 × 4.6 mm, 2.0 mL/min) with EtOH/H_2_O (55:45, *v*/*v*) as an eluent system yielded pure **6** (2.0 mg, *t*_R_ 39.6 min).

### 3.4. Spectral Data of New Compounds

*Pacificusoside L* (**1**), C_41_H_62_O_15_, amorphous powder; [α]d^25^ –54.3° (c 0.1, MeOH); IR (KBr): *ν*_max_ = 3440, 2929, 1772, 1632, 1455, 1247, 1069, 898 cm^−1^; ^1^H and ^13^C NMR data are listed in Table 1, Table 2 and Table 3; (+)ESIMS/MS of the ion [M + Na]^+^ at *m*/*z* 817: 685 [(M + Na)–C_5_H_8_O_4_]^+^; 539 [(M + Na)–C_5_H_8_O_4_–C_6_H_10_O_4_]^+^; 451 [carbohydrate chain + Na]^+^; 301 [(carbohydrate chain + Na)–C_5_H_8_O_4_–H_2_O]^+^; 169 [(carbohydrate chain + Na)–2×C_5_H_8_O_4_–H_2_O]^+^; (+)HRESIMS *m*/*z* 817.3978 [M + Na]^+^ (calculated for [C_41_H_62_O_15_Na]^+^, 817.3978); (−)ESIMS/MS of the ion [M–H]^−^ at *m*/*z* 793: 661 [(M − H)−C_5_H_8_O_4_]^−^; 515 [(M−H)−C_5_H_8_O_4_−C_6_H_10_O_4_]^−^; 383 [(M − H)–2×C_5_H_8_O_4_−C_6_H_10_O_5_]^−^; (−)HRESIMS *m*/*z* 793.4024 [M − H]^−^ (calculated for [C_41_H_61_O_15_]^−^, 793.4016).

*Pacificusoside M* (**2**), C_55_H_90_O_22_, amorphous powder; [α]d^25^ +4.0° (c 0.1, MeOH); IR (KBr): *ν*_max_ = 3426, 2925, 2854, 1716, 1632, 1421, 1274, 1114, 1065 cm^−1^; ^1^H and ^13^C NMR data are listed in Table 1, Table 2 and Table 3; (+)ESIMS/MS of the ion [M + Na]^+^ at *m*/*z* 1125: 1065 [(M + Na)–C_2_H_4_O_2_]^+^; 627 [carbohydrate chain + Na]^+^; 477 [(carbohydrate chain + Na)–C_5_H_8_O_4_–H_2_O]^+^; 331 [(carbohydrate chain + Na)–C_6_H_10_O_4_–C_5_H_8_O_4_–H_2_O]^+^; 185 [(carbohydrate chain + Na)–C_5_H_8_O_4_–H_2_O–2 × C_6_H_10_O_4_]^+^; (+)HRESIMS *m*/*z* 1125.5802 [M + Na]^+^ (calculated for [C_55_H_90_O_22_Na]^+^, 1125.5816); (−)ESIMS/MS of the ion [M − H]^−^ at *m*/*z* 1101: 1059 [(M − H)–C_2_H_2_O]^−^; 913 [(M − H)–C_2_H_2_O–C_6_H_10_O_4_]^−^; 751 [(M − H)–C_2_H_2_O–C_6_H_10_O_4_–C_6_H_10_O_5_]^−^; 605 [(M − H)–C_2_H_2_O–2 × C_6_H_10_O_4_–C_6_H_10_O_5_]^−^; 473 [(M − H)–C_2_H_2_O–2×C_6_H_10_O_4_–C_6_H_10_O_5_–C_5_H_8_O_4_]^−^; (−)HRESIMS *m*/*z* 1101.5857 [M − H]^−^ (calculated for [C_55_H_89_O_22_]^−^, 1101.5851).

*Pacificusoside N* (**3**), C_53_H_88_O_21_, amorphous powder; [α]d^25^ +6.2° (c 0.1, MeOH); ^1^H and ^13^C NMR data are listed in Table 1, Table 2 and Table 3; (+)ESIMS/MS of the ion [M + Na]^+^ at *m*/*z* 1083: 627 [carbohydrate chain + Na]^+^; 477 [(carbohydrate chain + Na)–C_5_H_8_O_4_–H_2_O]^+^; 331 [(carbohydrate chain + Na)–C_6_H_10_O_4_–C_5_H_8_O_4_–H_2_O]^+^; 185 [(carbohydrate chain + Na)–C_5_H_8_O_4_–H_2_O–2 × C_6_H_10_O_4_]^+^; (+)HRESIMS *m*/*z* 1083.5693 [M + Na]^+^ (calculated for [C_53_H_88_O_21_Na]^+^, 1083.5710); (−)ESIMS/MS of the ion [M − H]^−^ at *m*/*z* 1059: 913 [(M − H)–C_6_H_10_O_4_]^−^; 751 [(M − H)–C_6_H_10_O_4_−C_6_H_10_O_5_]^−^; 605 [(M − H)–2 × C_6_H_10_O_4_–C_6_H_10_O_5_]^−^; 473 [(M − H)–2 × C_6_H_10_O_4_–C_6_H_10_O_5_–C_5_H_8_O_4_]; (−)HRESIMS *m*/*z* 1059.5728 [M − H]^−^ (calculated for [C_53_H_87_O_21_]^−^, 1059.5745).

*Pacificusoside O* (**4**), C_53_H_88_O_22_, amorphous powder; [α]d^25^ +7.1° (c 0.07, MeOH); IR (KBr): *ν*_max_ = 3439, 2925, 2854, 1632, 1421, 1272, 1114, 1051 cm^−1^; ^1^H and ^13^C NMR data are listed in Table 1, Table 2 and Table 3; (+)ESIMS/MS of the ion [M + Na]^+^ at *m*/*z* 1099: 627 [carbohydrate chain + Na]^+^; 477 [(carbohydrate chain + Na)–C_5_H_8_O_4_–H_2_O]^+^; 331 [(carbohydrate chain + Na)–C_6_H_10_O_4_–C_5_H_8_O_4_–H_2_O]^+^; 185 [(carbohydrate chain + Na)–C_5_H_8_O_4_–H_2_O–2 × C_6_H_10_O_4_]^+^; (+)HRESIMS *m*/*z* 1099.5624 [M + Na]^+^ (calculated for [C_53_H_88_O_22_Na]^+^, 1099.5659); (−)ESIMS/MS of the ion [M − H]^−^ at *m*/*z* 1075: 929 [(M − H)–C_6_H_10_O_4_]^−^; 767 [(M − H)–C_6_H_10_O_4_–C_6_H_10_O_5_]^−^; 621 [(M − H)−2 × C_6_H_10_O_4_−C_6_H_10_O_5_]^−^; 489 [(M − H)−2 × C_6_H_10_O_4_–C_6_H_10_O_5_–C_5_H_8_O_4_]; (−)HRESIMS *m*/*z* 1075.5664 [M − H]^−^ (calculated for [C_53_H_87_O_22_]^−^, 1075.5694).

*Pacificusoside P* (**5**), C_53_H_86_O_22_, amorphous powder; [α]d^25^ +7.1° (c 0.07, MeOH); IR (KBr): *ν*_max_ = 3439, 2925, 2854, 1632, 1421, 1272, 1114, 1051cm^−1^; ^1^H and ^13^C NMR data are listed in Table 1, Table 2 and Table 3; (+)ESIMS/MS of the ion [M + Na]^+^ at *m*/*z* 1097: 627 [carbohydrate chain + Na]^+^; 477 [(carbohydrate chain + Na)–C_5_H_8_O_4_–H_2_O]^+^; 331 [(carbohydrate chain + Na)–C_6_H_10_O_4_–C_5_H_8_O_4_–H_2_O]^+^; 185 [(carbohydrate chain + Na)–C_5_H_8_O_4_–H_2_O–2 × C_6_H_10_O_4_]^+^; (+)HRESIMS *m*/*z* 1097.5501 [M + Na]^+^ (calculated for [C_53_H_86_O_22_Na]^+^, 1097.5503); (−)ESIMS/MS of the ion [M − Na]^−^ at *m*/*z* 1073: 927 [(M − H)–C_6_H_10_O_4_]^−^; 765 [(M − H)–C_6_H_10_O_4_–C_6_H_10_O_5_]^−^; 619 [(M − H)–2 × C_6_H_10_O_4_–C_6_H_10_O_5_]^−^; 487 [(M − H)–2 × C_6_H_10_O_4_–C_6_H_10_O_5_–C_5_H_8_O_4_]; (−)HRESIMS *m*/*z* 1073.5542 [M − H]^−^ (calculated for [C_53_H_85_O_22_]^−^, 1073.5538).

*Pacificusoside Q* (**6**), C_55_H_84_O_22_, amorphous powder; [α]d^25^ +8.5° (c 0.12, MeOH); ^1^H and ^13^C NMR data are listed in Table 1, Table 2 and Table 3; (+)ESIMS/MS of the ion [M + Na]^+^ at *m*/*z* 1119: 1059 [(M + Na)–C_2_H_4_O_2_]^+^; 627 [carbohydrate chain + Na]^+^; 477 [(carbohydrate chain + Na)–C_5_H_8_O_4_–H_2_O]^+^; 331 [(carbohydrate chain + Na)–C_6_H_10_O_4_–C_5_H_8_O_4_–H_2_O]^+^; 185 [(carbohydrate chain + Na)–C_5_H_8_O_4_–H_2_O–2 × C_6_H_10_O_4_]^+^; (+)HRESIMS *m*/*z* 1119.5345 [M + Na]^+^ (calculated for [C_55_H_84_O_22_Na]^+^, 1119.5346); (−)ESIMS/MS of the ion [M − H]^−^ at *m*/*z* 1095: 949 [(M − H)–C_6_H_10_O_4_]^−^; 787 [(M − H)–C_6_H_10_O_4_–C_6_H_10_O_5_]^−^; 641 [(M−H)−2 × C_6_H_10_O_4_–C_6_H_10_O_5_]^−^; 509 [(M − H)–2 × C_6_H_10_O_4_–C_6_H_10_O_5_–C_5_H_8_O_4_]; (−)HRESIMS *m*/*z* 1095.5381 [M − H]^−^ (calculated for [C_55_H_83_O_22_]^−^, 1095.5381).

### 3.5. Acid Hydrolysis and Determination of Absolute Configurations of Monosaccharides

Absolute configurations of monosaccharides of compounds **1** (1.5 mg) and **2** (2.0 mg) were determined by a method published earlier [5,6].

### 3.6. Reagents

The Basal Medium Eagle (BME), the Dulbecco’s Modified Eagle’s Medium (DMEM), the McCoy’s 5A Modified Medium (McCoy’s 5A), trypsin, and fetal bovine serum (FBS) were purchased from Thermo Fisher Scientific (Waltham, MA, USA). The phosphate buffered saline (PBS), L-glutamine, and penicillin-streptomycin solution (10,000 U/mL, 10 µg/mL) were purchased from Sigma-Aldrich (St. Louis, MO, USA). The MTS reagent 3-[4,5-dimethylthiazol-2-yl]-2,5-diphenyltetrazolium bromide was purchased from Promega (Madison, WI, USA).

### 3.7. Cell Lines and Culture Conditions

The human embryonic kidney HEK 293 cells (ATCC^®^ CRL-1573™), breast cancer MDA-MB-231 cells (ATCC^®^ HTB-26™), and colorectal carcinoma HCT 116 cells (ATCC^®^ CCL-247™) were purchased from the American Type Culture Collection (Manassas, VA, USA). The HEK 293 and MDA-MB-231 cells were cultured in DMEM; the HCT 116 cells were cultured in the McCoy’s 5A medium at 37 °C in a humidified atmosphere containing 5% CO_2_. The culture media were supplemented with 10% heat-inactivated FBS and a 1% penicillin/streptomycin solution. The number of passages was carefully controlled, and contamination by *Mycoplasma* was monitored on a regular basis.

### 3.8. Preparation of Compounds

Compounds **1**–**3**, and **6**–**8** were dissolved in sterile dimethyl sulfoxide (DMSO), to prepare stock concentrations of 20 mM. Cells were treated with serially diluted **1**–**3**, **6**–**8** (0.3–100 µM) (with the culture medium used as diluent) (the final concentration of DMSO was less than 0.5%). The vehicle control was the cells treated with an equivalent volume of DMSO (the final concentration was less than 0.5%) for all of the presented experiments.

### 3.9. MTS Assay

HEK 293 (1.0 × 10^4^/200 µL), HCT 116 (1.0 × 10^4^/200 µL), and MDA-MB-231 (0.8 × 10^4^/200 µL) cells were seeded in a 96-well plate and incubated for 24 h at 37 °C in a CO_2_ incubator. To determine the concentration at which the compounds exerted half of their maximal inhibitory effect on cell viability (IC_50_), the cells were treated with either DMSO (vehicle control) or cisplatin (positive control) at 1, 5, 10, 50, 100 µM for 24 and 48 h, or compounds **1**, **2**, **3**, **6**, **7**, and **8** at concentrations of 3.125, 6.25, 12.5, 25, 50, and 100 µM for 24 h. The cells were subsequently incubated with 15 µL of the MTS reagent for 3 h. The absorbance of each well was measured at 490/630 nm on a Power Wave XS microplate reader (BioTek, Winooski, VT, USA).

The IC_50_ concentration was calculated using the AAT-Bioquest^®^ online calculator [24]. The selectivity index (SI) was calculated as described previously [25], using the following formula: SI = IC_50_ of the compounds for normal cells (HEK 293) divided by IC_50_ of the same compounds for the human colorectal adenocarcinoma and breast cancer (HCT 116 and MDA-MB-231) cell lines. Both IC_50_ and SI values are provided in Table 4.

### 3.10. Soft Agar Assay

HCT 116 and MDA-MB-231 cells (2.4 × 10^4^/mL) were treated with compound **2** (0.6, 1.25, 2.5, and 5 µM). Then, the cells were placed onto 0.3% BME agar containing 10% FBS, 2 mM L-glutamine, and 25 µg/mL gentamicin. The cultures were maintained at 37 °C in a 5% CO_2_ incubator for 14 days. The number of colonies was counted under an AE 20 Motic microscope using the ImageJ v. 1.50i software, bundled with 64-bit Java 1.6.0_24 (NIH, Bethesda, MD, USA).

### 3.11. Scratch Assay

HCT 116 and MDA-MB-231 cells (3 × 10^5^ cells/mL) were seeded in 6-well plates and grown to 80% confluence for 24 h. After removing the culture medium, the cells’ monolayer was scraped with a 200 µL sterile pipette tip, to create a straight scratch. Then, the cells were treated with compound **2** at concentrations of 0.6, 1.25, 2.5, and 5 µM and incubated for 24, 48, and 72 h. All experiments were set up in triplicate for each group. For image analysis, the cell migration into the wound area was photographed at the stages of 0, 24, 48, and 72 h through a Motic AE 20 microscope and using the ImageJ v. 1.50i software, bundled with 64-bit Java 1.6.0_24 (NIH, Bethesda, MD, USA). The cell migration distance was estimated by measuring the width of the wound, and was expressed as the percentage of each control (0 h) in relation to the mean of the wound-closure area.

### 3.12. Statistical Analysis

All the assays were performed in at least triplicate. The results were expressed as mean ± standard deviation (SD). The obtained data were statistically processed by the one-way analysis of variance (ANOVA) and the Tukey’s HSD test with significance levels of * *p* < 0.05, ** *p* < 0.01, and *** *p* < 0.001.

## Figures and Tables

**Table 1:** ^1^H (700.13 MHz) NMR data of aglycons of compounds **1**–**6** (35 °C, C_5_D_5_N, *J* in Hz) ^a^.

Position	1	2	3	4	5	6
1	1.47 m	1.68 m 1.18 m	1.68 m 1.18 m	1.69 m 1.18 m	1.69 m 1.18 m	1.72 m 1.22 m
2	2.18 m 1.91 m	2.16 m 1.89 m	2.17 m 1.89 m	2.17 m 1.89 m	2.17 m 1.89 m	2.23 m 1.97 m
3	3.32 dd (12.0; 3.3)	3.27 dd (12.0, 4.3)	3.27 dd (12.0, 3.7)	3.27 m	3.27 m	3.32 dd (11.5, 3.9)
4						
5	1.00 dd (12.0; 3.3)	1.12 m	1.13 brd (12.6)	1.13 m	1.13 m	1.81 m
6	2.04 m 1.97 m	1.73 m 1.53 m	1.72 m 1.51 m	1.72 m 1.51 m	1.72 m 1.51 m	1.72 m 1.68 m
7	5.62 brd (7.0)	1.96 m 0.96 m	2.07 m 1.03 m	2.03 m 1.04 m	2.03 m 1.04 m	2.15 m 1.18 m
8						
9	3.00 brd (14.0)					
10						
11	1.99 m 1.46 m	2.28 m 2.13 m	2.28 m 2.13 m	2.28 m 2.15 m	2.28 m 2.15 m	2.30 m 2.08 m
12	2.37 m 1.88 m	2.77 m 1.70 m	2.77 m 1.70 m	2.77 m 1.70 m	2.77 m 1.70 m	2.23 m 1.75 m
13						
14						
15	2.15 dd (13.5, 2.3) 1.97 m	2.26 m 1.76 m	2.07 m	2.10 m 2.04 m	2.10 m 2.04 m	2.03 dd (11.8, 6.9) 1.82 m
16	4.74 s	5.85 m	5.02 m	5.03 m	5.03 m	5.81 m
17	2.93 s	2.32 brd (7.0)	2.15 m	2.17 m	2.10 m	2.75 d (9.3)
18		4.17 m 3.93 m	4.41 brd (11.2) 3.97 brd (11.2)	4.42 brd (11.2) 3.97 brd (11.2)	4.42 brd (11.2) 3.97 brd (11.2)	
19	1.03 s	1.05 s	1.04 s	1.04 s	1.04 s	1.31 s
20						
21	1.74 s	1.62 s	1.63 s	1.67 s	1.60 s	1.56 s
22	5.06 s 4.99 s	1.92 m 1.84 m	2.15 m	2.42 m 2.28 m	2.72 m 2.28 m	5.93 d (15.8)
23		2.32 m 2.22 m	2.35 m 2.25 m	2.18 m 2.03 m	3.17 m 2.97 m	6.58 dd (15.8, 11.3)
24		5.30 m	5.28 m	4.47 brd (9.2)		5.90 d (11.3)
25						
26		1.67 s	1.64 s	5.26 brs 4.94 brs	6.08 brs 5.67 brs	1.64 s
27		1.67 s	1.69 s	1.93 s	1.94 s	1.72 s
30	1.18 s	1.11 s	1.10 s	1.12 s	1.12 s	1.15 s
31	1.32 s	1.32 s	1.32 s	1.34 s	1.34 s	1.34 s
32	1.33 s	1.02 s	1.02 s	1.00 s	1.00 s	1.07 s
CH_3_-CO		2.17 s				2.06 s

^a^ Assignments from 700.13 MHz ^1^H-^1^H COSY, HSQC, HMBC (8 Hz), and ROESY (270 ms) data.

**Table 2:** ^13^C (176.04 MHz) NMR data of aglycons of compounds **1**–**6** (35 °C, C_5_D_5_N).

Position	1	2	3	4	5	6
1	35.9	35.6	35.8	35.6	35.6	35.8
2	27.0	27.1	27.3	27.1	27.1	27.0
3	89.0	88.7	88.9	88.7	88.7	88.8
4	39.4	39.6	39.7	39.6	39.6	39.6
5	47.4	50.7	51.1	50.9	50.9	50.9
6	23.3	18.1	18.1	18.1	18.1	17.9
7	122.7	26.3	26.3	26.3	26.3	27.7
8	147.3	132.7	133.5	133.5	133.5	134.2
9	46.4	136.5	136.4	136.4	136.4	136.0
10	35.5	36.9	37.0	36.9	36.9	37.0
11	21.7	20.9	21.0	20.8	20.8	21.2
12	20.0	25.4	25.5	25.3	25.3	28.3
13	56.7	51.2	51.3	51.1	51.1	45.0
14	46.0	46.5	47.0	46.9	46.9	59.3
15	43.8	41.5	44.1	44.0	44.0	40.8
16	80.4	76.6	73.5	73.2	73.2	73.6
17	59.0	54.0	54.1	54.0	55.2	53.4
18	180.7	62.2	62.8	62.6	62.6	176.5
19	23.9	19.2	19.2	19.1	19.1	19.0
20	139.9	74.6	76.6	76.6	76.1	82.4
21	23.0	27.6	27.9	26.2	25.5	30.5
22	113.9	42.9	43.2	35.9	36.8	134.2
23		23.7	23.8	31.1	32.8	122.2
24		125.0	125.5	75.8	201.9	125.3
25		131.1	131.0	148.7	144.4	134.5
26		17.4	17.5	110.0	124.2	18.2
27		25.6	25.7	17.5	17.7	25.6
30	17.3	16.3	16.3	16.3	16.3	16.3
31	28.6	27.7	27.7	27.7	27.7	27.1
32	33.9	26.4	26.2	26.2	26.2	26.5
CO		170.1				170.5
CH_3_-CO		21.4				21.0

**Table 3:** ^1^H (700.13 MHz), ^13^C (176.04 MHz), HMBC, and ROESY NMR data of the carbohydrate chains of **1**–**6** (35 °C, C_5_D_5_N, *J* in Hz) ^a^.

Position	1				2–6			
	*δ*_H_	*δ*_C_	HMBC	ROESY	*δ*_H_	*δ*_C_	HMBC	ROESY
	***Xyl-I***				***Xyl***			
1	4.88 d (7.1)	105.1	C3-agl; C5-Xyl-I	H3, H31-agl, H3, H5-Xyl-I	4.79 d (7.5)	105.5	C3-agl	H3, H31-agl, H3, H5-Xyl
2	4.01 dd (8.7, 7.4)	83.3	C1, C3-Xyl-I, C1-Qui	H1-Qui	4.05 dd (8.2, 7.2)	84.0	C1-Xyl, C1-Qui	H1-Qui
3	4.26 t (9.1)	77.9	C2, C4-Xyl-I	H1-Xyl-I	4.19 t (9.0)	78.0	C1, C2-Xyl	H1-Xyl
4	4.18 m	70.2			4.16 m	70.7		
5	4.35 dd (11.2, 5.1) 3.74 m	66.5	C1, C3-Xyl-I C1, C3-Xyl-I	H1-Xyl-I	4.30 dd (11.6, 5.1) 3.69 t (10.0)	66.6	C3, C4-Xyl	H1-Xyl
	***Qui***				***Qui***			
1	5.31 d (7.5)	103.4	C2-Xyl-I	H3, H5-Qui, H2-Xyl-I	5.16 d (7.5)	105.5	C2-Xyl	H3, H5-Qui, H2-Xyl
2	4.14 m	84.8	C1, C3-Qui, C1-Xyl-II	H4-Qui, H1-Xyl-II	4.06 m	76.3	C1, C3-Qui	
3	4.19 m	77.5	C2,C4-Qui	H1, H5-Qui	4.11 t (9.1)	75.8	C2, C4-Qui	H1-Qui
4	3.70 m	76.3	C2, C3, C5-Qui	H2, H6-Qui	3.67 t (9.1)	87.3	C3-Qui, C1-Glc	H1-Glc, H6-Qui
5	3.70 m	72.7		H1, H3-Qui	3.80 m	71.6		H1-Qui
6	1.64 d (5.0)	18.3	C4, C5-Qui	H4-Qui	1.76 d (6.0)	18.1	C4, C5-Qui	H4-Qui
	***Xyl-II***				***Glc***			
1	5.35 d (7.2)	106.4	C2-Qui	H3, H5-Xyl-II; H2-Qui	4.97 d (8.0)	104.9	C4-Qui	H4-Qui, H3, H5-Glc
2	4.08 m	75.8	C1, C3-Xyl-II		4.02 m	73.9	C1, C3-Glc	
3	4.10 m	77.6	C4-Xyl-II	H1-Xyl-II	4.20 m	87.2	C4-Glc	H1-3-OMe-Xyl, H1, H5-Glc
4	4.11 m	70.5			4.02 m	69.5	C3, C5-Glc	
5	4.31 dd (11.6, 5.0) 3.67 m	67.3	C1, C3, C4-Xyl-II C1, C3, C4-Xyl-II	H1-Xyl-II	4.01 m	77.9		H1, H3-Glc
6					4.50 brd (12.2) 4.20 m	62.1		
					***3-OMe-Xyl***			
1					5.20 d (7.7)	106.0	C3-Glc	H3, H5-3-OMe-Xyl; H3-Glc
2					3.93 t (8.1)	74.6	C1, C3-3-OMe-Xyl	H4-3-OMe-Xyl
3					3.59 t (8.9)	87.7	C2, C4-3-OMe-Xyl, OMe	H1-3-OMe-Xyl; OMe
4					4.06 m	70.0		H2-3-OMe-Xyl
5					4.20 dd (10.6, 5.0) 3.63 m	66.9	C3, C4-3-OMe-Xyl C1, C3-3-OMe-Xyl	H1-3-OMe-Xyl
3-OMe					3.85 s	60.5	C3-3-OMe-Xyl	H3-3-OMe-Xyl

^a^ Assignments from 700.13 MHz ^1^H-^1^H COSY, HSQC, HMBC (8 Hz), and ROESY (270 ms) data.

**Table 4:** Cytotoxicity and selectivity index of compounds **1**–**3** and **6**–**8** from the sun star *S. pacificus* and cisplatin.

Compound	HEK 293	HCT 116		MDA-MB-231	
	IC_50_, µM	IC_50_, µM	SI	IC_50_, µM	SI
**Cisplatin**	64.6 ± 1.5 *	11.4 ± 1.3 *	1.6	34.5 ± 0.5 *	1.9
**1**	27 ± 0.3	81 ± 3.2	ns	51.2 ± 0.8	ns
**2**	18.5 ± 2.5	18.6 ± 1.4	1	15.5 ± 1.6	1.2
**3**	52.8 ± 0.8	42.2 ± 0.4	1.25	36.7 ± 3.3	1.4
**6**	15.3 ± 1.2	25.5 ± 1.4	ns	29.8 ± 2.1	ns
**7**	28.5 ± 1.5	35.6 ± 0.6	ns	34 ± 2.9	ns
**8**	6.2 ± 0.7	11.4 ± 2.7	ns	13.1 ± 4.2	ns

IC_50_, the concentration of compounds that caused a 50% reduction in cell viability of tested normal and cancer cells during 24 h of cell incubation. Values are indicated as mean ± standard deviation. * IC_50_ of cisplatin during 48 h of cell incubation. SI, the selectivity index was calculated using the following equation: SI = mean IC_50_ against normal cells/mean IC_50_ against cancer cells. ns, non-selective.

## Data Availability

The data presented in this study are available on request from the corresponding authors.

## Supplementary Materials

The following supporting information can be downloaded at: https://www.mdpi.com/article/10.3390/md22010019/s1, Figure S1: HRESIMS spectrum of pacificusoside L (**1**); Figure S2: IR spectrum of pacificusoside L (**1**) in KBr; Figure S3: ^1^H NMR spectrum of pacificusoside L (**1**) in C_5_D_5_N; Figure S4: ^13^C NMR spectrum of pacificusoside L (**1**) in C_5_D_5_N; Figure S5: ^1^H-^1^H COSY spectrum of pacificusoside L (**1**) in C_5_D_5_N; Figure S6: HSQC spectrum of pacificusoside L (**1**) in C_5_D_5_N; Figure S7: HMBC spectrum of pacificusoside L (**1**) in C_5_D_5_N; Figure S8: ROESY spectrum of pacificusoside L (**1**) in C_5_D_5_N; Figure S9: (−)ESIMS/MS spectrum of pacificusoside L (**1**); Figure S10: (+)ESIMS/MS spectrum of pacificusoside L (**1**); Figure S11: GC chromatogram of acetylated 2-octylglycosides of the hydrolysate of pacificusoside L (**1**); Figure S12: GC chromatogram of acetylated 2-octylglycosides of D-xylose; Figure S13: GC chromatogram of acetylated 2-octylglycosides of D-quinovose; Figure S14: GC chromatogram of acetylated 2-octylglycosides of L-xylose; Figure S15: GC chromatogram of acetylated 2-octylglycosides of L-quinovose; Figure S16: HRESIMS spectrum of pacificusoside M (**2**); Figure S17: IR spectrum of pacificusoside M (**2**) in KBr; Figure S18: ^1^H NMR spectrum of pacificusoside M (**2**) in C_5_D_5_N; Figure S19: ^13^C NMR spectrum of pacificusoside M (**2**) in C_5_D_5_N; Figure S20: ^1^H-^1^H COSY spectrum of pacificusoside M (**2**) in C_5_D_5_N; Figure S21: HSQC spectrum of pacificusoside M (**2**) in C_5_D_5_N; Figure S22: HMBC spectrum of pacificusoside M (**2**) in C_5_D_5_N; Figure S23: ROESY spectrum of pacificusoside M (**2**) in C_5_D_5_N; Figure S24: (−)ESIMS/MS spectrum of pacificusoside M (**2**); Figure S25: (+)ESIMS/MS spectrum of pacificusoside M (**2**); Figure S26: HRESIMS spectrum of pacificusoside N (**3**); Figure S27: ^1^H NMR spectrum of pacificusoside N (**3**) in C_5_D_5_N; Figure S28: ^13^C NMR spectrum of pacificusoside N (**3**) in C_5_D_5_N; Figure S29: ^1^H-^1^H COSY spectrum of pacificusoside N (**3**) in C_5_D_5_N; Figure S30: HSQC spectrum of pacificusoside N (**3**) in C_5_D_5_N; Figure S31: HMBC spectrum of pacificusoside N (**3**) in C_5_D_5_N; Figure S32: ROESY spectrum of pacificusoside N (**3**) in C_5_D_5_N; Figure S33: (−)ESIMS/MS spectrum of pacificusoside N (**3**); Figure S34: (+)ESIMS/MS spectrum of pacificusoside N (**3**); Figure S35: HRESIMS spectrum of pacificusosides O and P (**4** and **5**); Figure S36: IR spectrum of pacificusosides O and P (**4** and **5**) in KBr; Figure S37: ^1^H NMR spectrum pacificusosides O and P (**4** and **5**) in C_5_D_5_N; Figure S38: ^13^C NMR spectrum of pacificusosides O and P (**4** and **5**) in C_5_D_5_N; Figure S39: ^1^H-^1^H COSY spectrum of pacificusosides O and P (**4** and **5**) in C_5_D_5_N; Figure S40: HSQC spectrum of pacificusosides O and P (**4** and **5**) in C_5_D_5_N; Figure S41: HMBC spectrum of pacificusosides O and P (**4** and **5**) in C_5_D_5_N: Figure S42: ROESY spectrum of pacificusosides O and P (**4** and **5**) in C_5_D_5_N; Figure S43: (−)ESIMS/MS spectrum of pacificusoside O and P (**4** and **5**); Figure S44: (+)ESIMS/MS spectrum of pacificusoside O and P (**4** and **5**); Figure S45: HRESIMS spectrum of pacificusoside Q (**6**); Figure S46: ^1^H NMR spectrum of pacificusoside Q (**6**) in C_5_D_5_N; Figure S47: ^13^C NMR spectrum of pacificusoside Q (**6**) in C_5_D_5_N; Figure S48: ^1^H-^1^H COSY spectrum of pacificusoside Q (**6**) in C_5_D_5_N; Figure S49: HSQC spectrum of pacificusoside Q (**6**) in C_5_D_5_N; Figure S50: HMBC spectrum of pacificusoside Q (**6**) in C_5_D_5_N; Figure S51: ROESY spectrum of pacificusoside Q (**6**) in C_5_D_5_N; Figure S52: (−)ESIMS/MS spectrum of pacificusoside Q (**6**); Figure S53: (+)ESIMS/MS spectrum of pacificusoside Q (**6**).
