# Supplementary material for: New Rare Triterpene Glycosides from Pacific Sun Star, Solaster pacificus, and Their Anticancer Activity
Source: Mar Drugs. 2023 Dec 28;22(1):19. doi: 10.3390/md22010019 (PMC10820528; doi:10.3390/md22010019)

Supplementary Materials

New Rare Triterpene Glycosides from Pacific Sun Star, *Solaster pacificus,* and Their Anticancer Activity

Timofey V. Malyarenko[1,2*], Alla A. Kicha[1], Alexandra S. Kuzmich[1], Olesya S. Malyarenko[1], Anatoly I. Kalinovsky[1], Roman S. Popov[1], Pavel S. Dmitrenok[1], Valentin A. Stonik[1*], and Natalia V. Ivanchina[1]

[1] G.B. Elyakov Pacific Institute of Bioorganic Chemistry, Far Eastern Branch, Russian Academy of Sciences, Prospect 100-let Vladivostoku 159, 690022 Vladivostok, Russia; kicha@piboc.dvo.ru (A.A.K.); assavina@mail.ru (A.S.K.); malyarenko.os@gmail.com (O.S.M.); kaaniw@piboc.dvo.ru (A.I.K.); prs_90@mail.ru (R.S.P.); paveldmt@piboc.dvo.ru (P.S.D.); ivanchina@piboc.dvo.ru (N.V.I.)

[2] Department of Bioorganic Chemistry and Biotechnology, School of Natural Sciences, Far Eastern Federal University, Russky Island, Ajax Bay 10, 690922, Vladivostok, Russia

* Correspondence: malyarenko-tv@mail.ru (T.V.M.); stonik@piboc.dvo.ru (V.A.S.). Tel.: +7-423-2312-360; Fax: +7-423-2314-050

List

Figure S1. HRESIMS spectrum of pacificusoside L (**1**).

Figure S2. IR spectrum of pacificusoside L (**1**) in KBr (transmittance vs wave number).

Figure S3. ^{1}H-NMR spectrum of pacificusoside L (**1**) in C_5D_5N.

Figure S4. ^{13}C-NMR spectrum of pacificusoside L (**1**) in C_5D_5N.

Figure S5. ^{1}H-^{1}H COSY spectrum of pacificusoside L (**1**) in C_5D_5N.

Figure S6. HSQC spectrum of pacificusoside L (**1**) in C_5D_5N.

Figure S7. HMBC spectrum of pacificusoside L (**1**) in C_5D_5N.

Figure S8. ROESY spectrum of pacificusoside L (**1**) in C5D5N.

Figure S9. (–)ESIMS/MS spectrum of pacificusoside L (**1**).

Figure S10. (+)ESIMS/MS spectrum of pacificusoside L (**1**).

Figure S11. GC chromatogram of acetylated 2-octylglycosides of the hydrolysate of **1**.

Figure S12. GC chromatogram of acetylated 2-octylglycosides of D-xylose (t_R 19.26, 19.42, and 19.73 min).

Figure S13. GC chromatogram of acetylated 2-octylglycosides of D-quinovose (t_R 18.89, 19.12, 19.47, and 19.75 min).

Figure S14. GC chromatogram of acetylated 2-octylglycosides of L-xylose (t_R 19.14, 19.67, and 19.95 min).

Figure S15. GC chromatogram of acetylated 2-octylglycosides of L-quinovose (t_R 18.73, 19.20, and 19.77 min).

Figure S16. HRESIMS spectrum of pacificusoside M (**2**).

Figure S17. IR spectrum of pacificusoside M (**2**) in KBr (transmittance vs wave number).

Figure S18. ^{1}H-NMR spectrum of pacificusoside M (**2**) in C_5D_5N.

Figure S19. ^{13}C-NMR spectrum of pacificusoside M (**2**) in C_5D_5N.

Figure S20. ^{1}H-^{1}H COSY spectrum of pacificusoside M (**2**) in C_5D_5N.

Figure S21. HSQC spectrum of pacificusoside M (**2**) in C_5D_5N.

Figure S22. HMBC spectrum of pacificusoside M (**2**) in C_5D_5N.

Figure S23. ROESY spectrum of pacificusoside M (**2**) in C_5D_5N.

Figure S24. (–)ESIMS/MS spectrum of pacificusoside M (**2**).

Figure S25. (+)ESIMS/MS spectrum of pacificusoside M (**2**).

Figure S26. HRESIMS spectrum of pacificusoside N (**3**).

Figure S27. ^{1}H-NMR spectrum of pacificusoside N (**3**) in C_5D_5N.

Figure S28. ^{13}C-NMR spectrum of pacificusoside N (**3**) in C_5D_5N.

Figure S29. ^{1}H-^{1}H COSY spectrum of pacificusoside N (**3**) in C_5D_5N.

Figure S30. HSQC spectrum of pacificusoside N (**3**) in C_5D_5N.

Figure S31. HMBC spectrum of pacificusoside N (**3**) in C_5D_5N.

Figure S32. ROESY spectrum of pacificusoside N (**3**) in C_5D_5N.

Figure S33. (–)ESIMS/MS spectrum of pacificusoside N (**3**).

Figure S34. (+)ESIMS/MS spectrum of pacificusoside N (**3**).

Figure S35. HRESIMS spectrum of pacificusosides O and P (**4** and **5**).

Figure S36. IR spectrum of pacificusosides O and P (**4** and **5**) in KBr (transmittance vs wave number).

Figure S37. ^{1}H-NMR spectrum of pacificusosides O and P (**4** and **5**) in C_5D_5N.

Figure S38. ^{13}C-NMR spectrum of pacificusosides O and P (**4** and **5**) in C_5D_5N.

Figure S39. ^1H-^1H COSY spectrum of pacificusosides O and P (**4** and **5**) in C_5D_5N.

Figure S40. HSQC spectrum of pacificusosides O and P (**4** and **5**) in C_5D_5N.

Figure S41. HMBC spectrum of pacificusosides O and P (**4** and **5**) in C_5D_5N.

Figure S42. ROESY spectrum of pacificusosides O and P (**4** and **5**) in C_5D_5N.

Figure S43. (−)ESIMS/MS spectrum of pacificusoside O and P (**4** and **5**).

Figure S44. (+)ESIMS/MS spectrum of pacificusoside O and P (**4** and **5**).

Figure S45. HRESIMS spectrum of pacificusoside Q (**6**).

Figure S46. ^1H-NMR spectrum of pacificusoside Q (**6**) in C_5D_5N.

Figure S47. ^{13}C-NMR spectrum of pacificusoside Q (**6**) in C_5D_5N.

Figure S48. ^1H-^1H COSY spectrum of pacificusoside Q (**6**) in C_5D_5N.

Figure S49. HSQC spectrum of pacificusoside Q (**6**) in C_5D_5N.

Figure S50. HMBC spectrum of pacificusoside Q (**6**) in C_5D_5N.

Figure S51. ROESY spectrum of pacificusoside Q (**6**) in C_5D_5N.

Figure S52. (−)ESIMS/MS spectrum of pacificusoside Q (**6**).

Figure S53. (+)ESIMS/MS spectrum of pacificusoside Q (**6**).

Figure S1. HRESIMS spectrum of pacificusoside L (**1**).

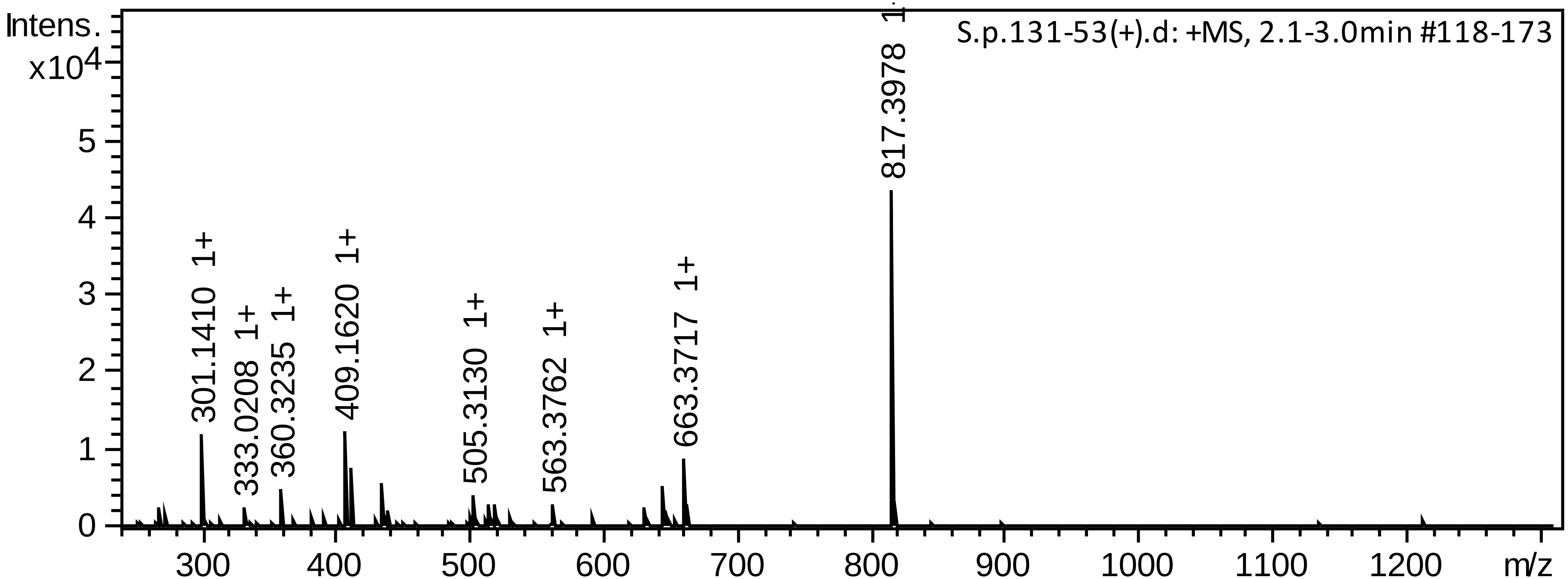

Figure S2. IR spectrum of pacificusoside L (**1**) in KBr (transmittance vs wave number).

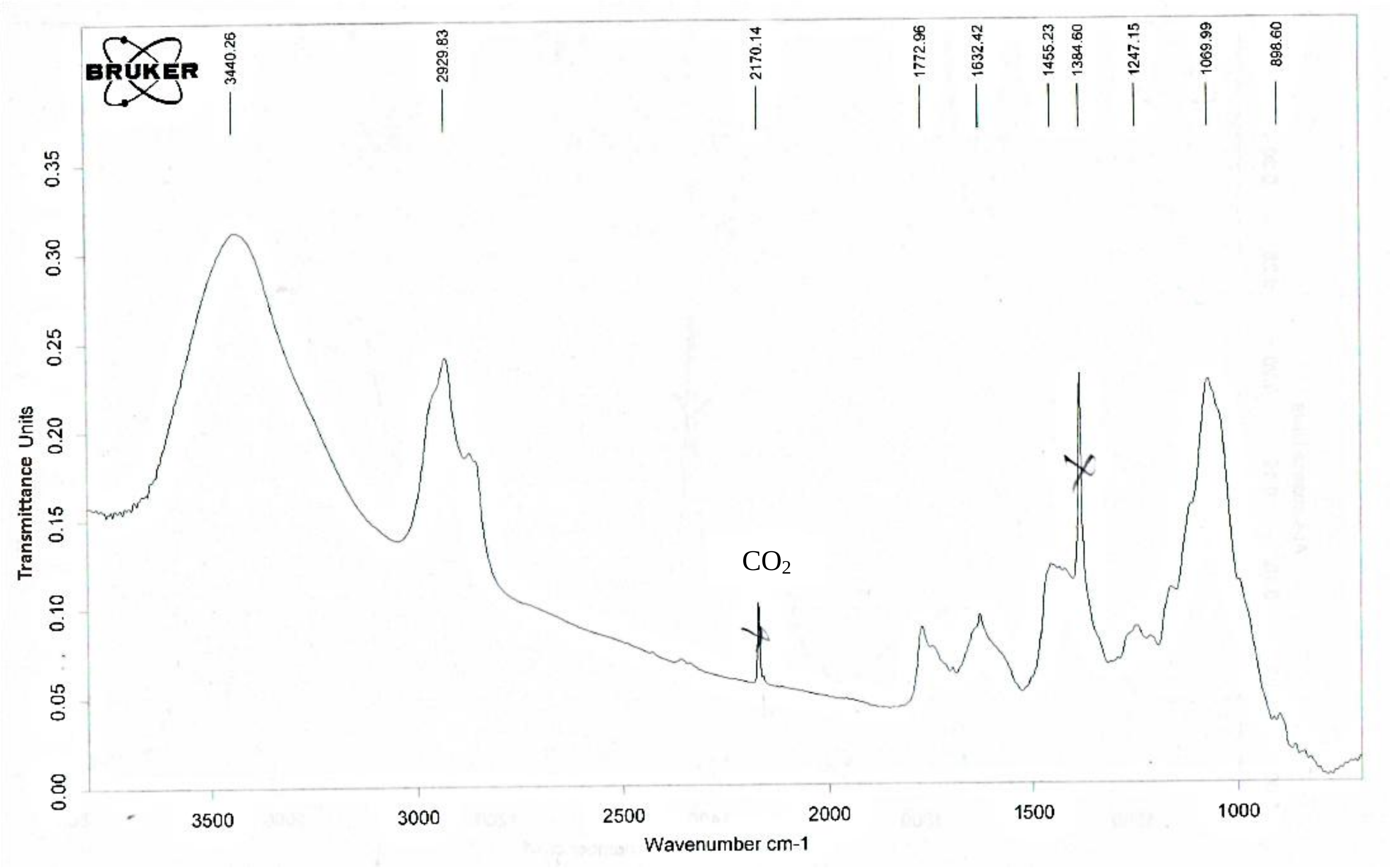

Figure S3. ^{1}H-NMR spectrum of pacificusoside L (**1**) in C_5D_5N.

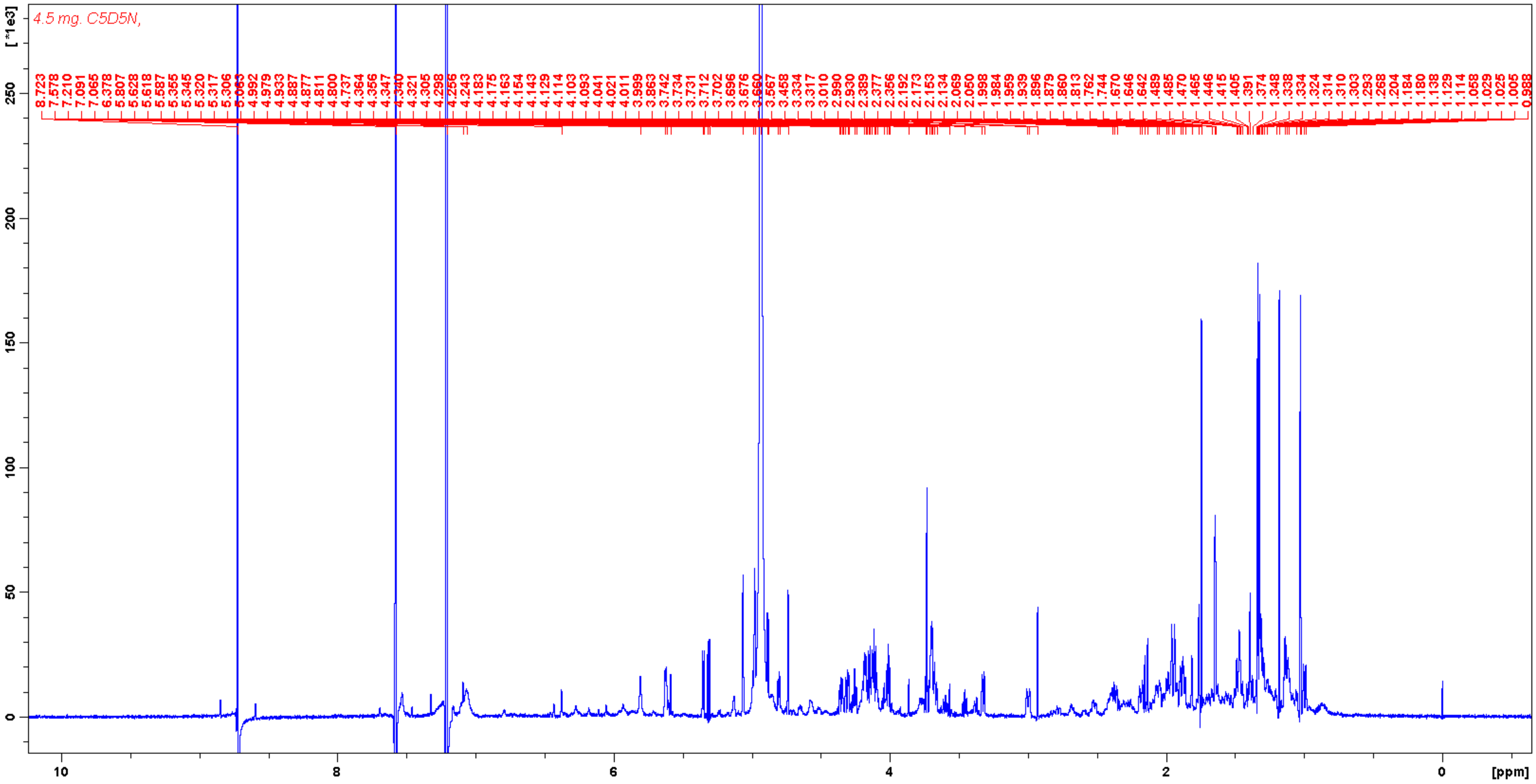

Figure S4. ^{13}C-NMR spectrum of pacificusoside L (**1**) in C_5D_5N.

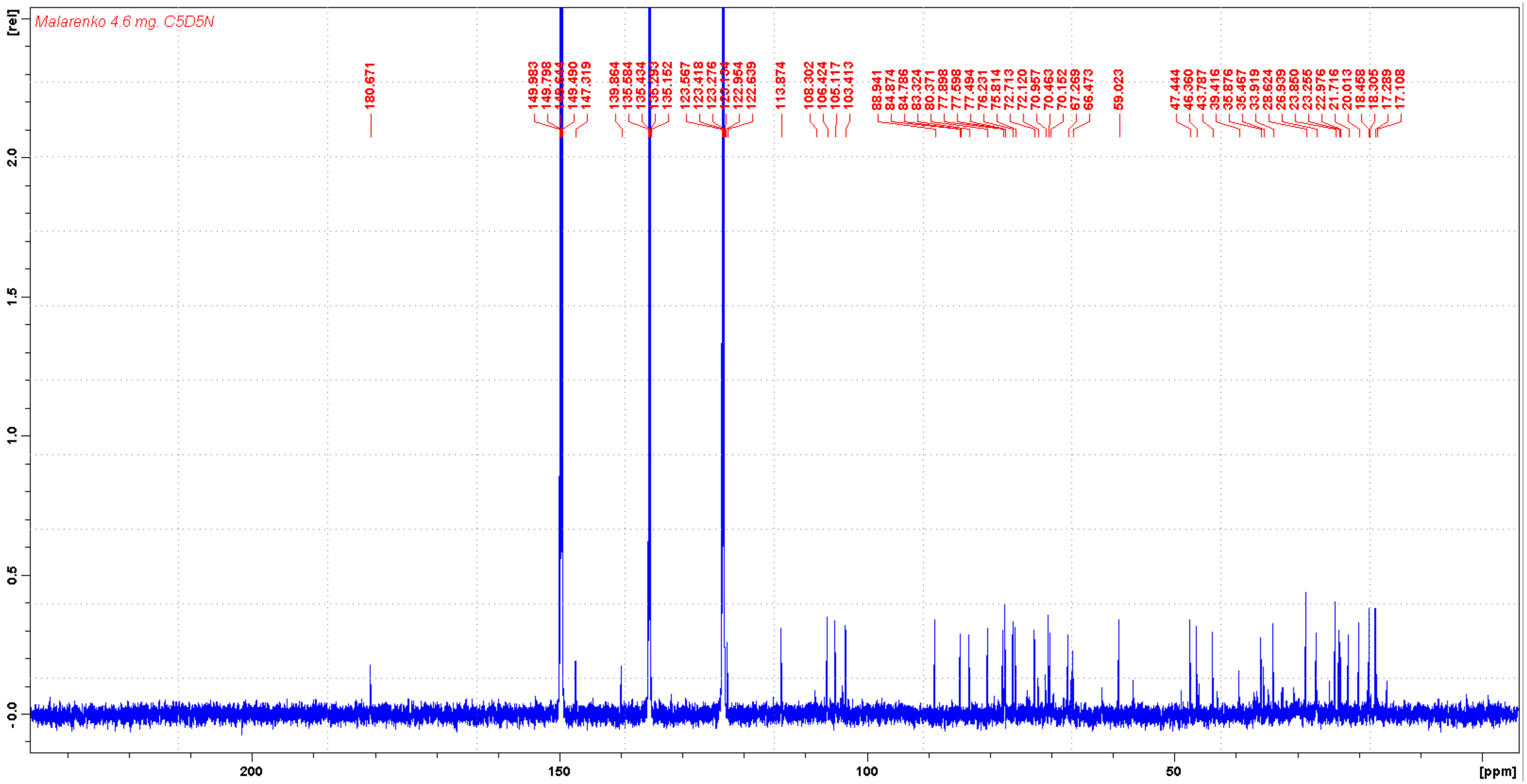

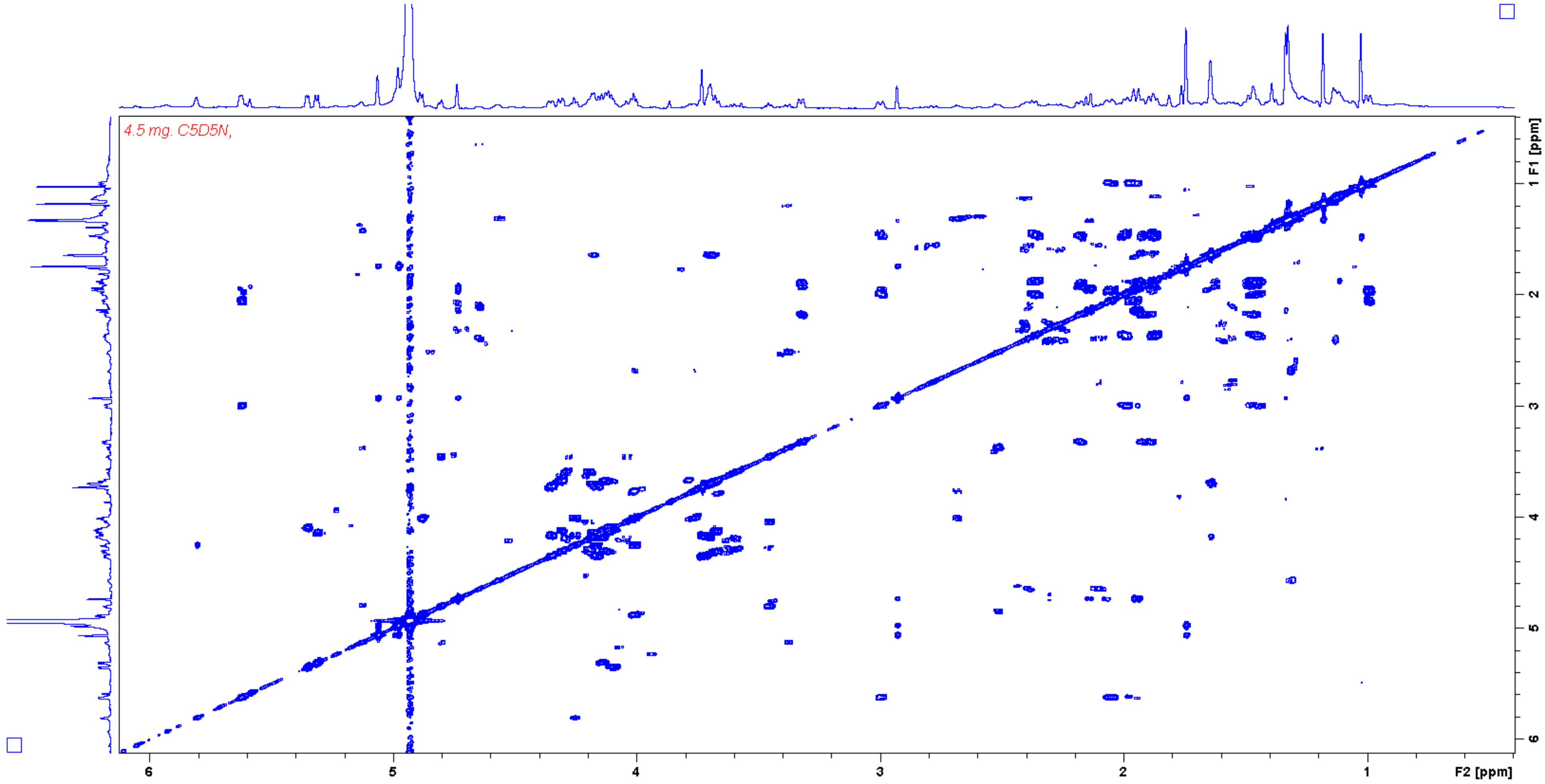

Figure S5. ^{1}H-^{1}H COSY spectrum of pacificusoside L (**1**) in C_5D_5N.

Figure S6. HSQC spectrum of pacificusoside L (**1**) in C_5D_5N.

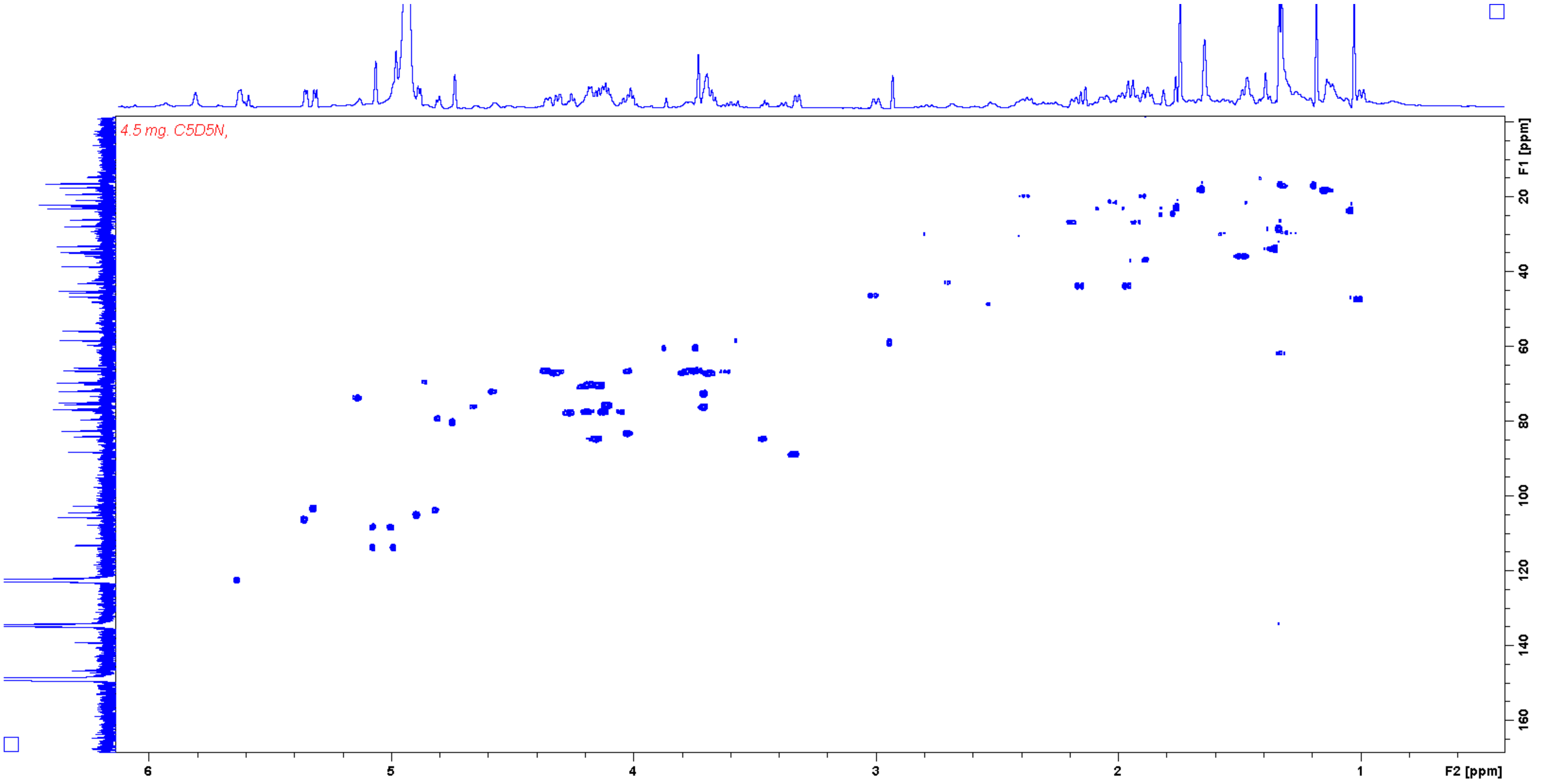

Figure S7. HMBC spectrum of pacificusoside L (**1**) in C_5D_5N.

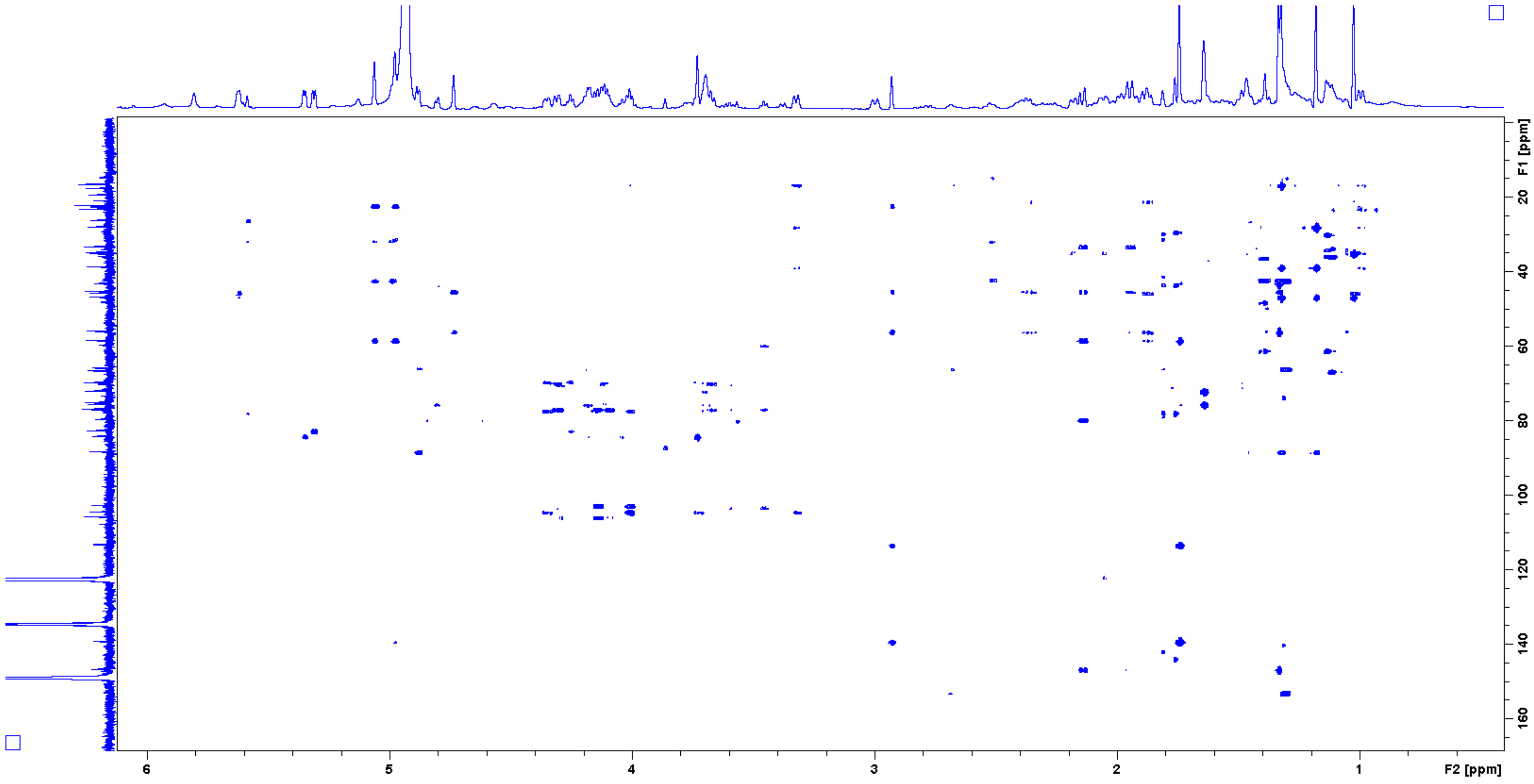

Figure S8. ROESY spectrum of pacificusoside L (**1**) in C_5D_5N.

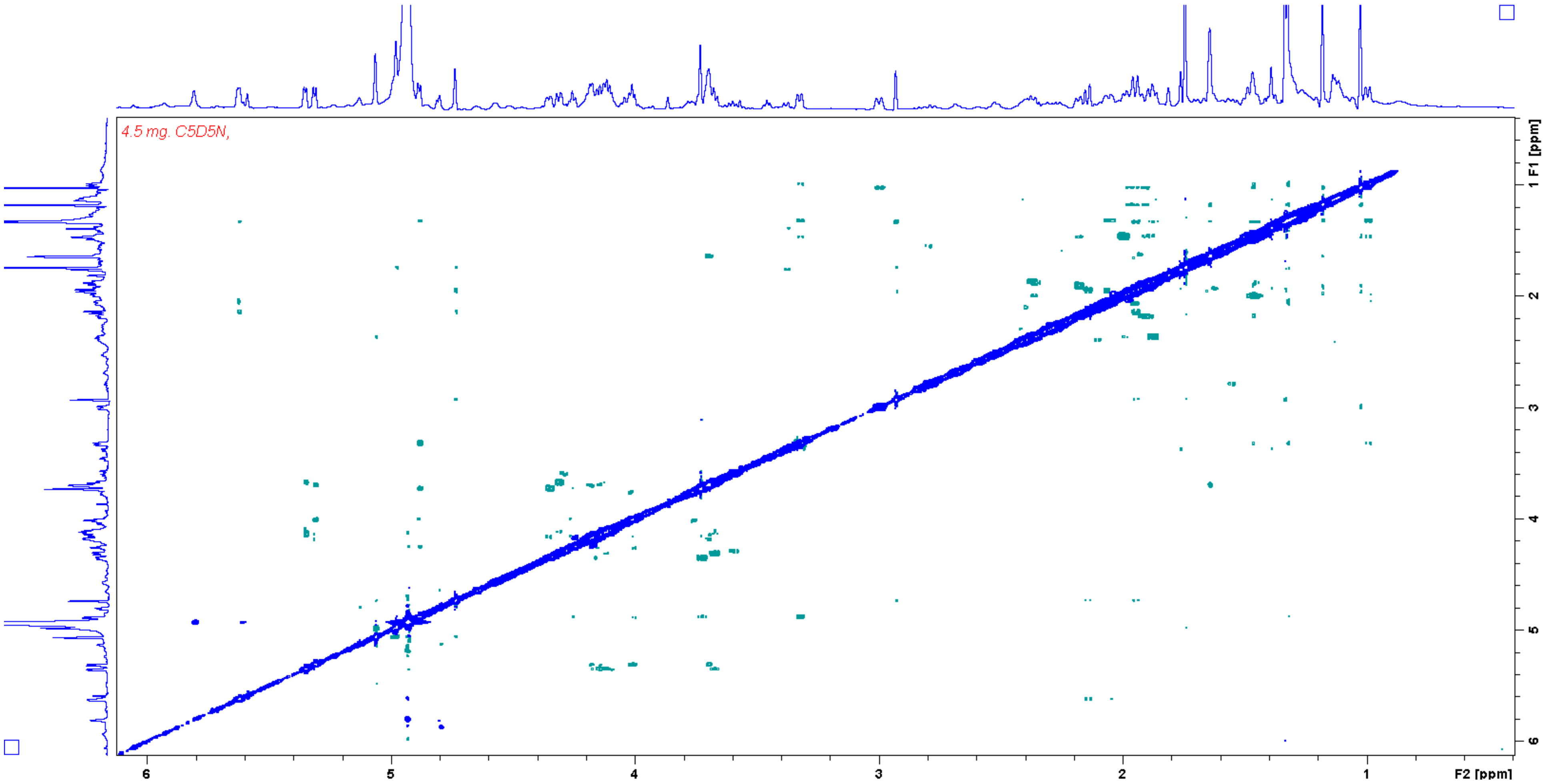

Figure S9. (–)ESIMS/MS spectrum of pacificusoside L (**1**).

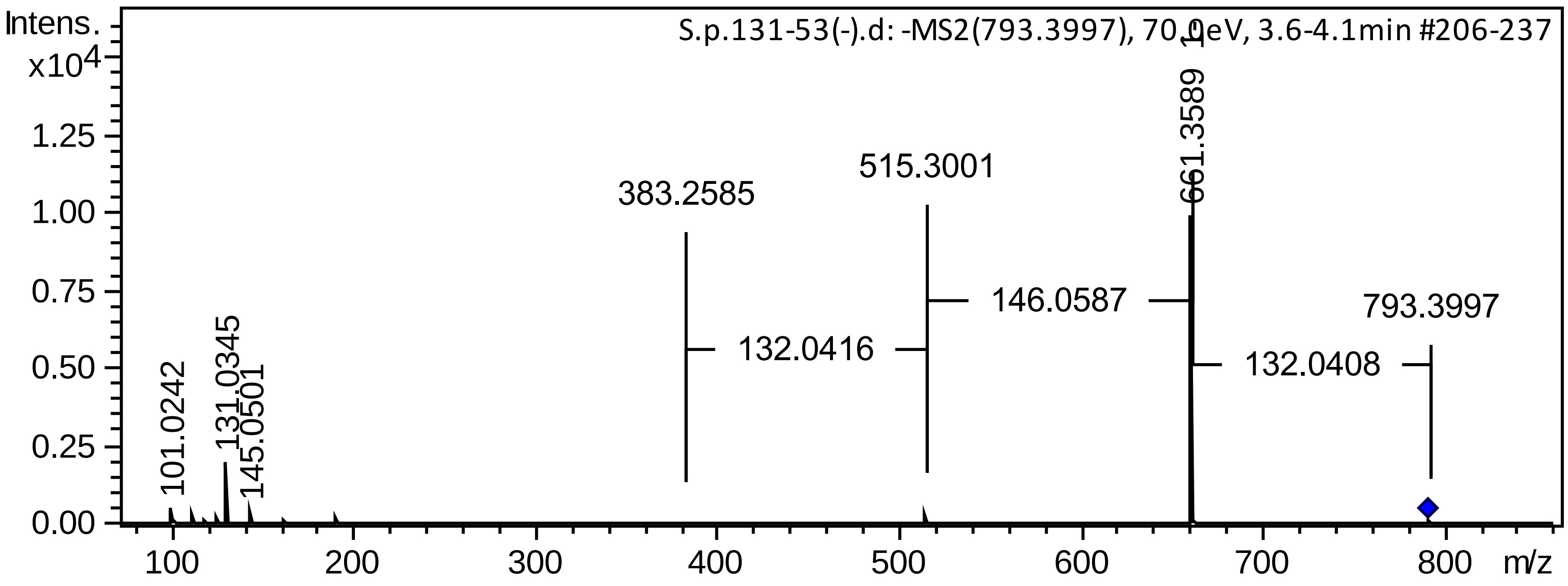

Figure S10. (+)ESIMS/MS spectrum of pacificusoside L (**1**).

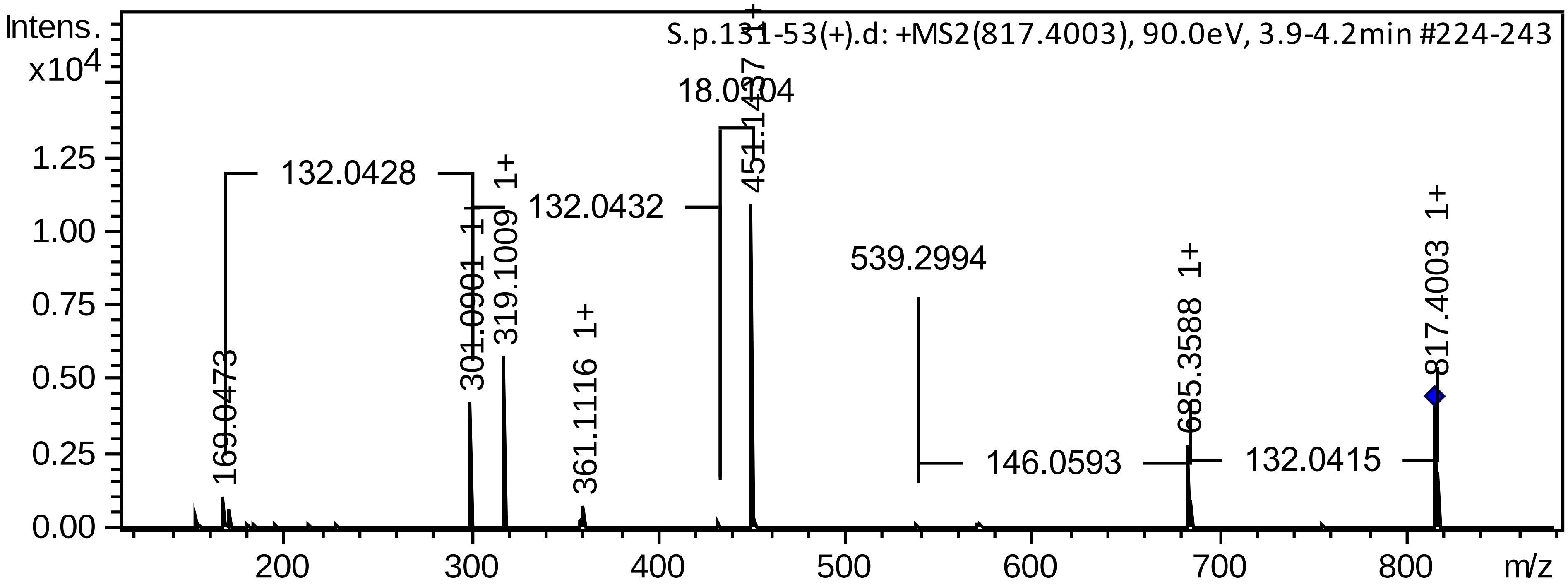

Figure S11. GC chromatogram of acetylated 2-octylglycosides of the hydrolysate of **1** (the following peaks were detected: D-xylose (t_R 19.27, 19.44, and 19.72 min) and D-quinovose (t_R 18.89, 19.09, 19.44, and 19.72 min)).

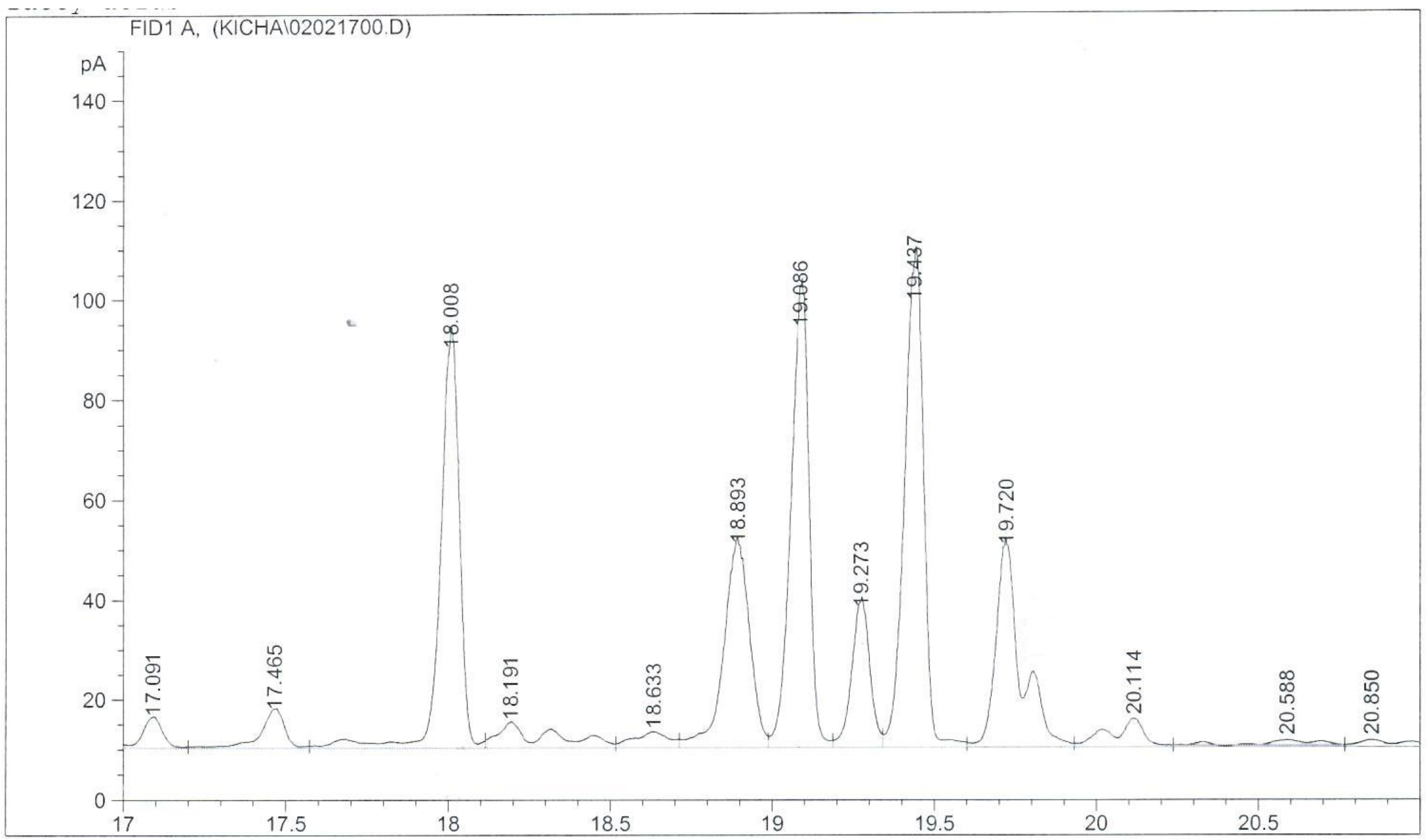

Figure S12. GC chromatogram of acetylated 2-octylglycosides of D-xylose (t_R 19.26, 19.42, and 19.73 min).

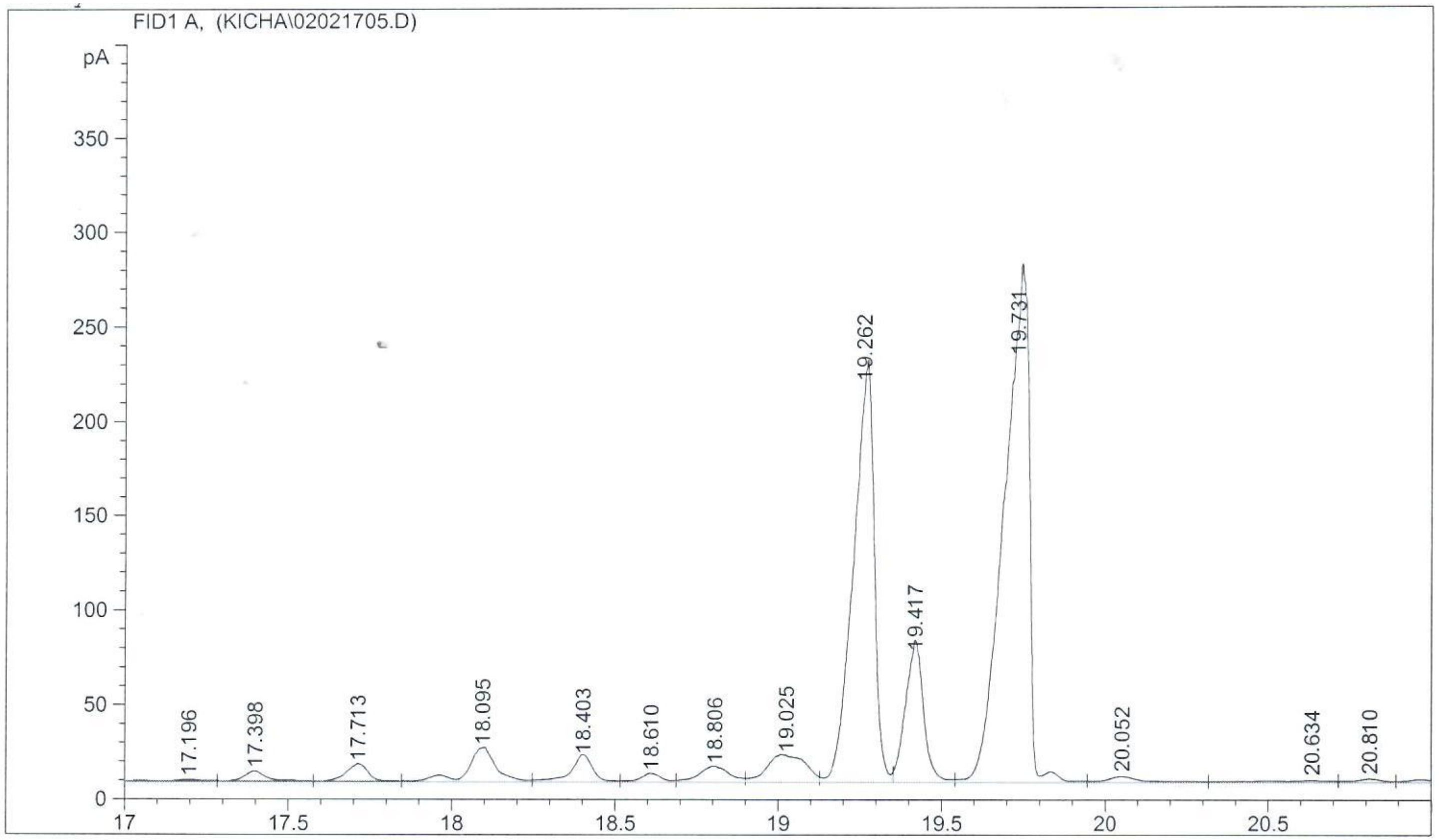

Figure S13. GC chromatogram of acetylated 2-octylglycosides of D-quinovose (t_R 18.89, 19.12, 19.47, and 19.75 min).

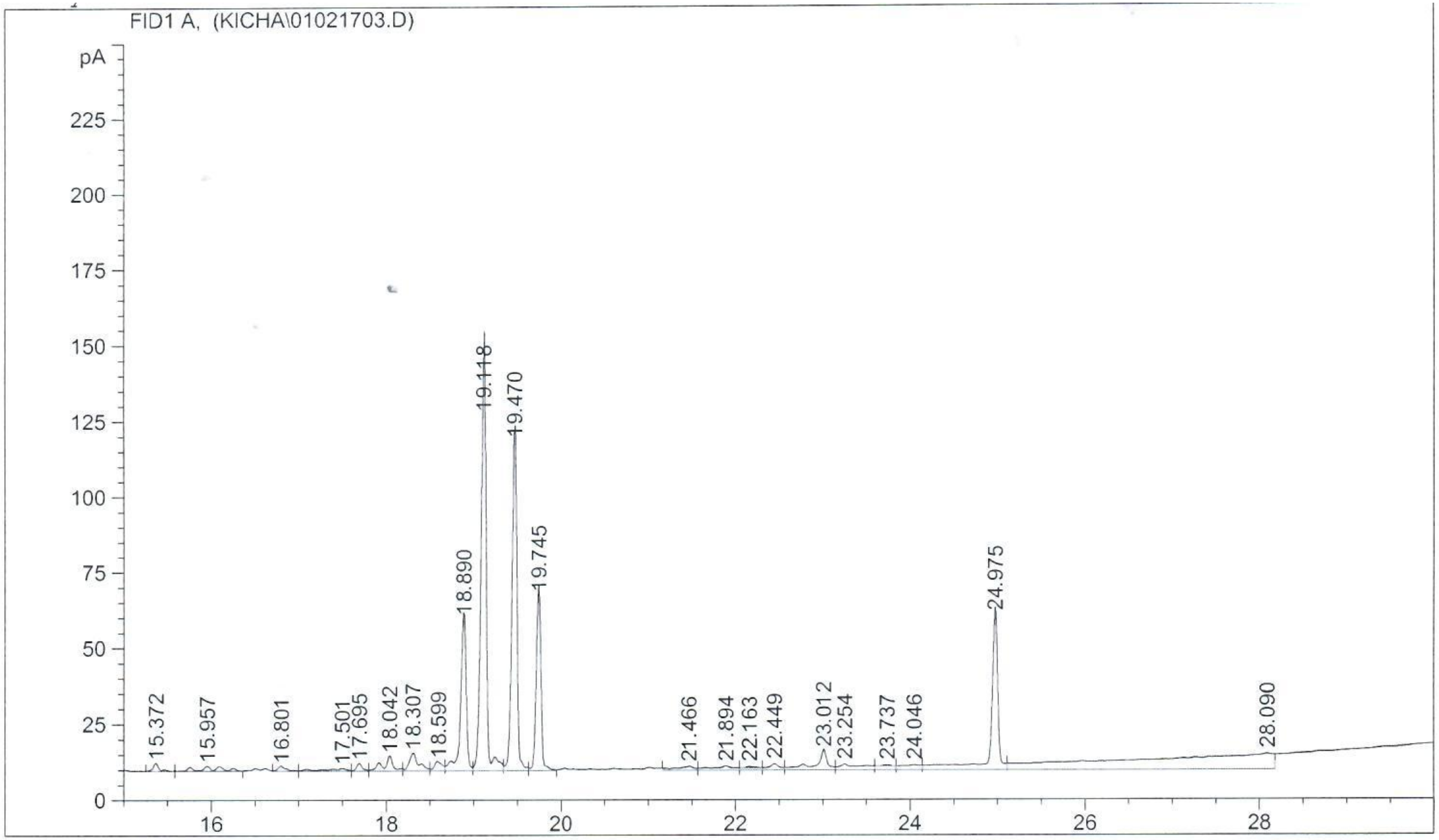

Figure S14. GC chromatogram of acetylated 2-octylglycosides of L-xylose (t_R 19.14, 19.67, and 19.95 min).

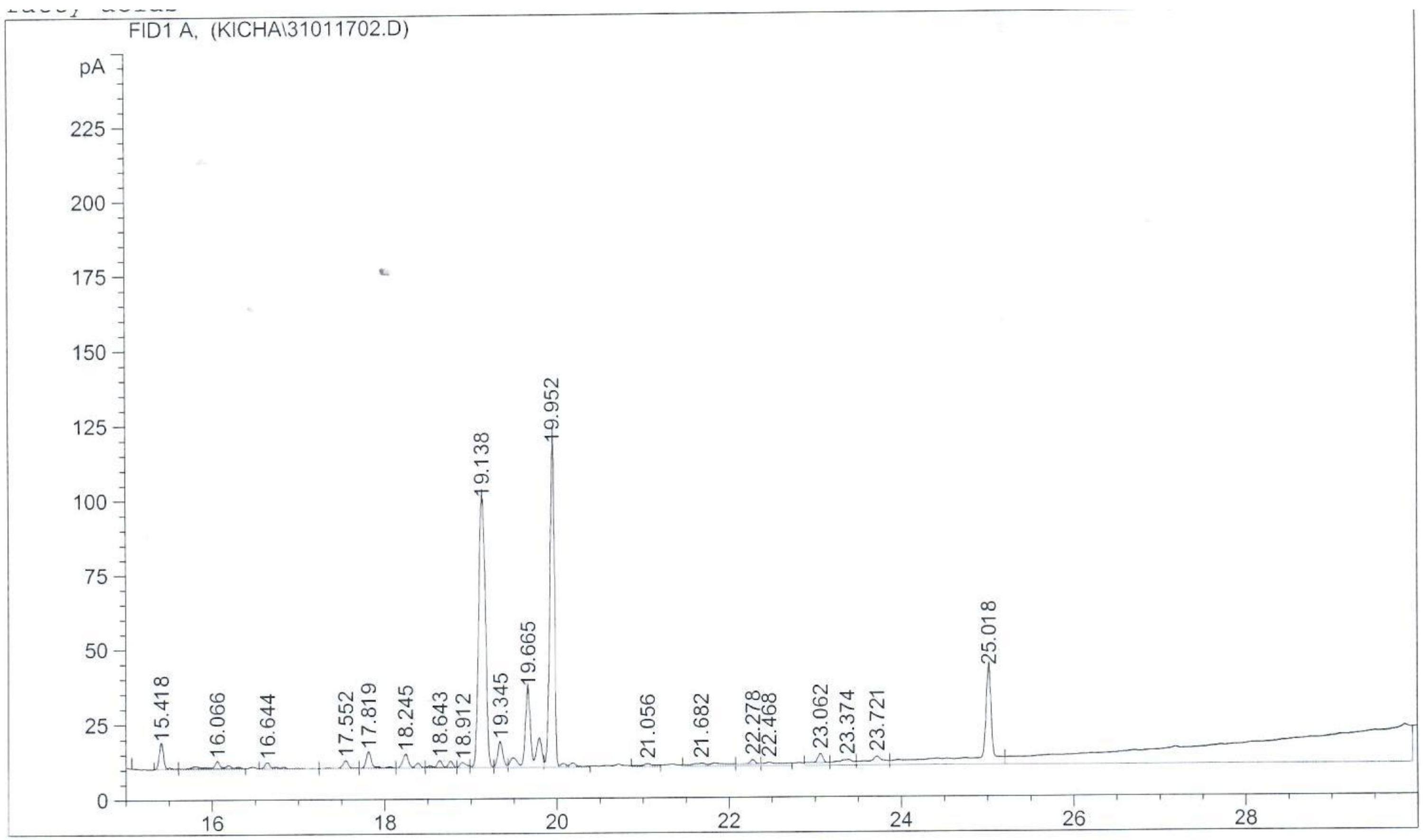

Figure S15. GC chromatogram of acetylated 2-octylglycosides of L-quinovose (t_R 18.73, 19.20, and 19.77 min).

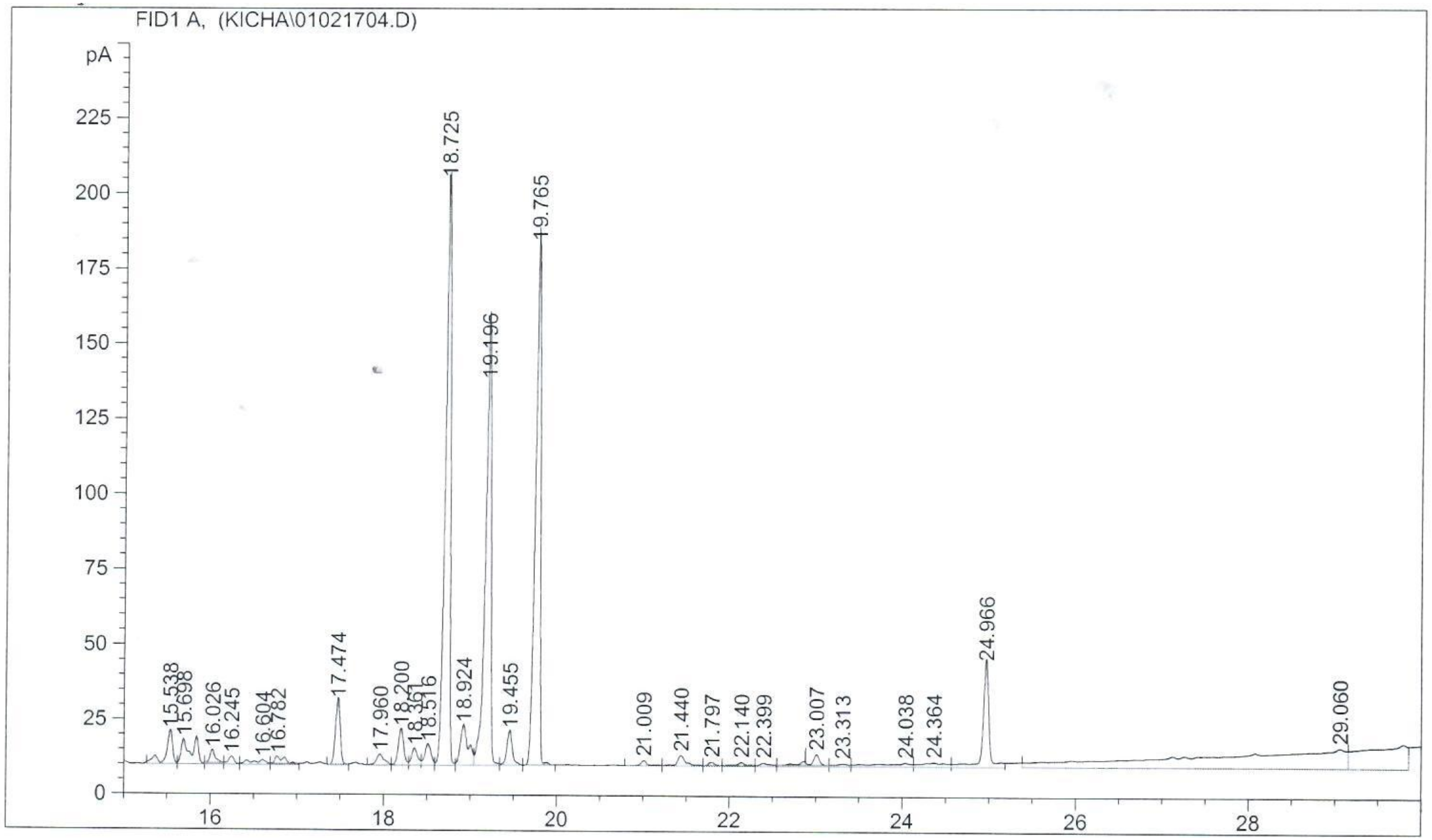

Figure S16. HRESIMS spectrum of pacificusoside M (**2**).

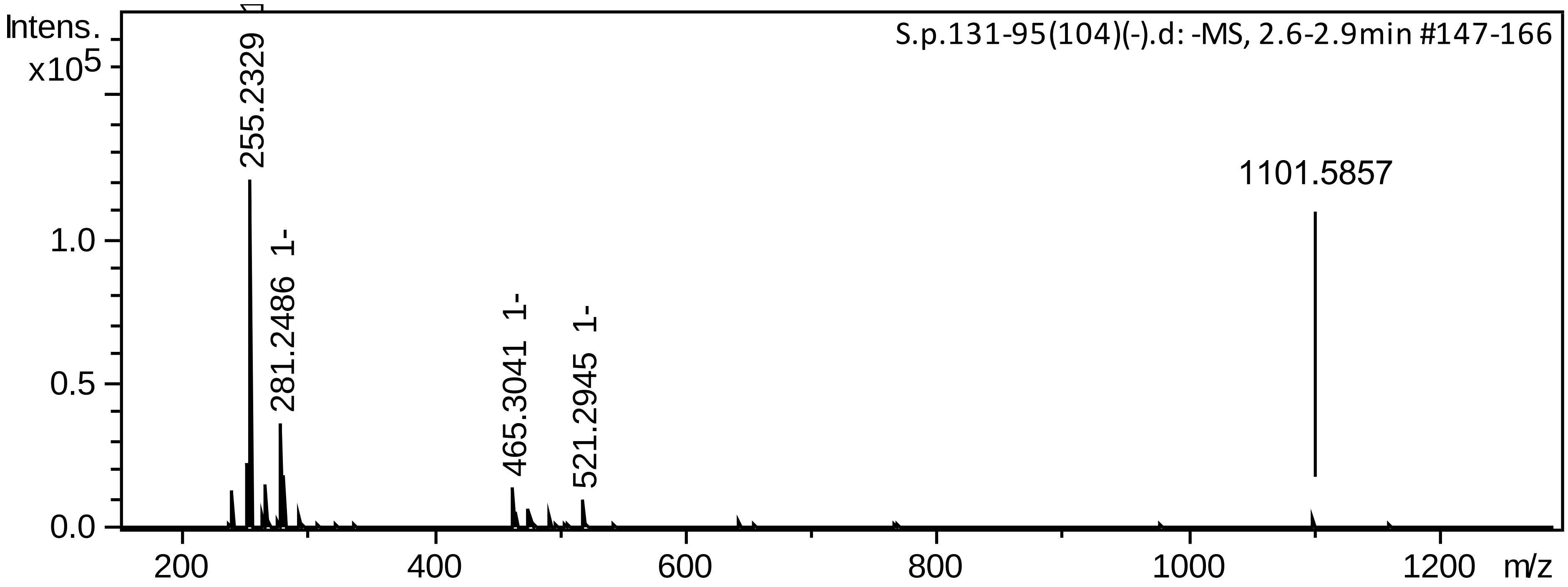

Figure S17. IR spectrum of pacificusoside M (**2**) in KBr (transmittance vs wave number).

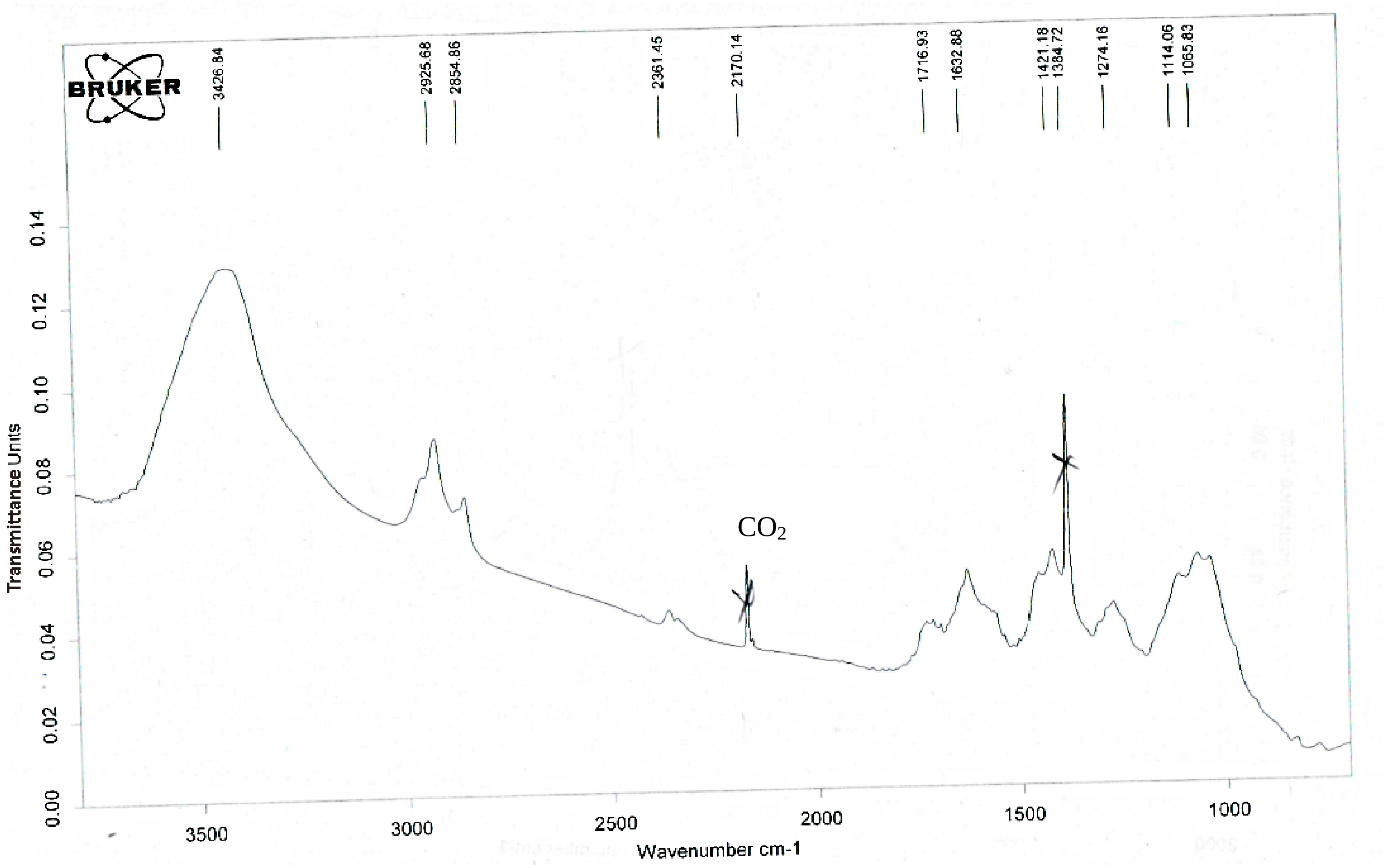

Figure S18. ^{1}H-NMR spectrum of pacificusoside M (**2**) in C_5D_5N.

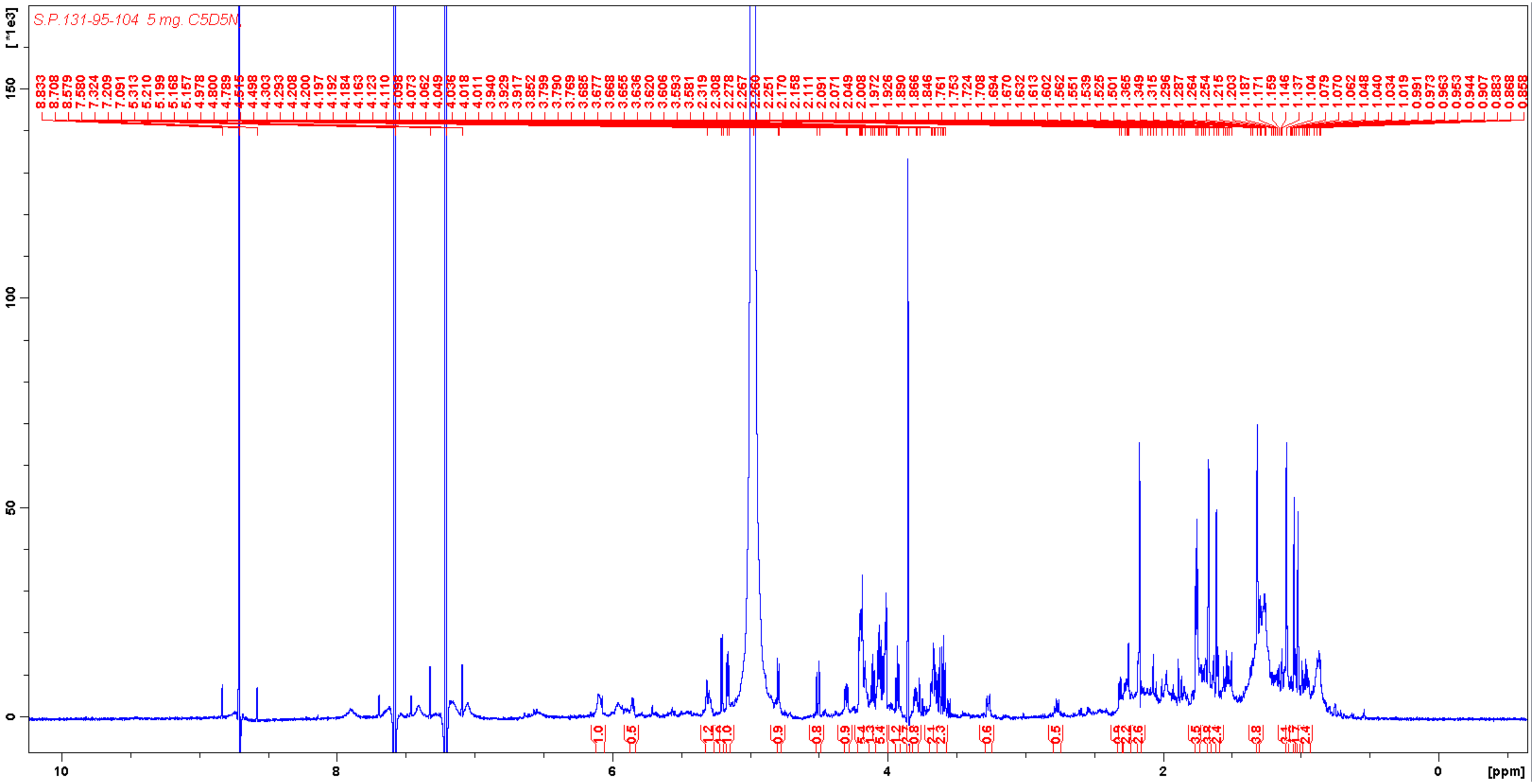

Figure S19. ^{13}C-NMR spectrum of pacificusoside M (**2**) in C_5D_5N.

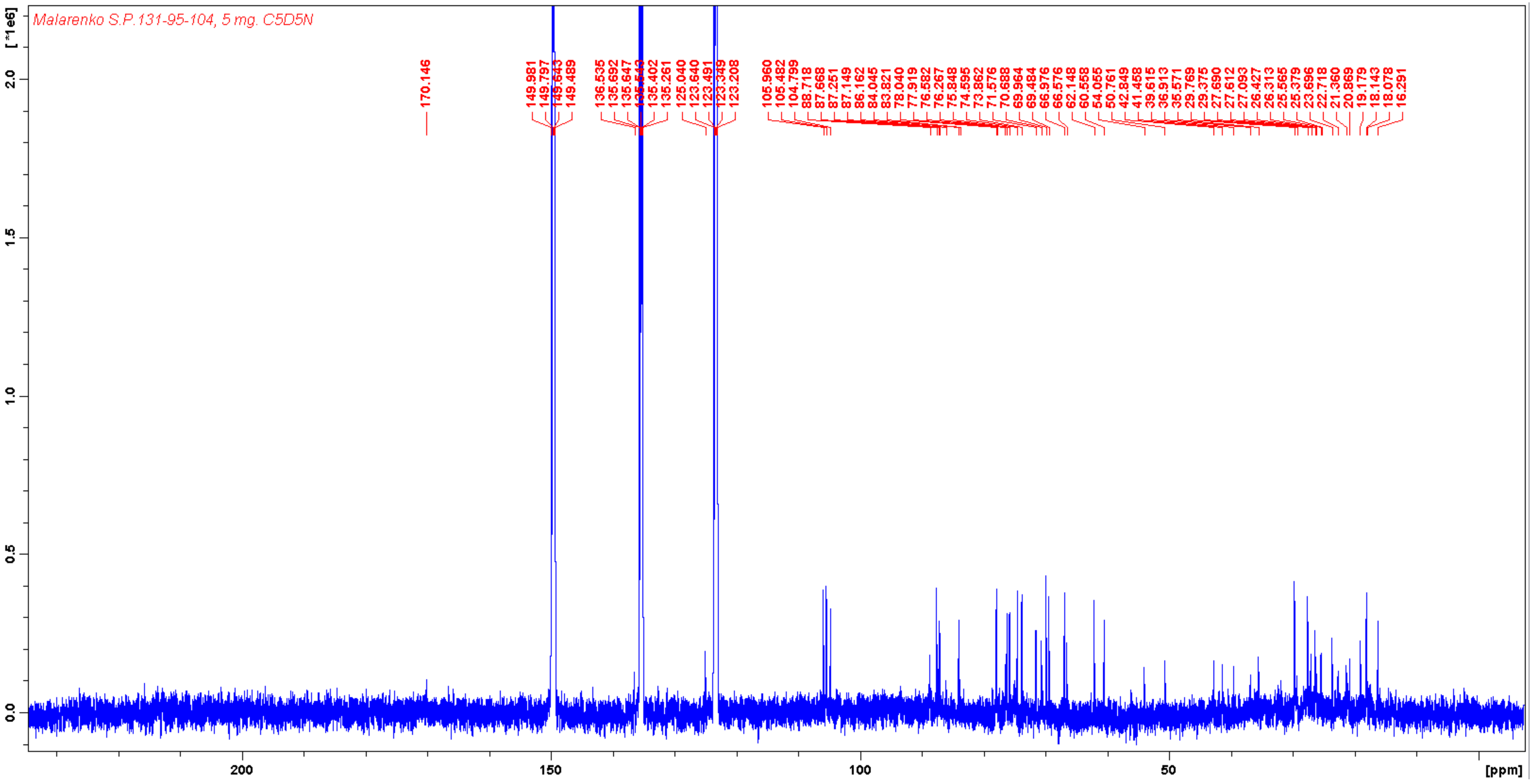

Figure S20. ^1H-^1H COSY spectrum of pacificusoside M (**2**) in C_5D_5N.

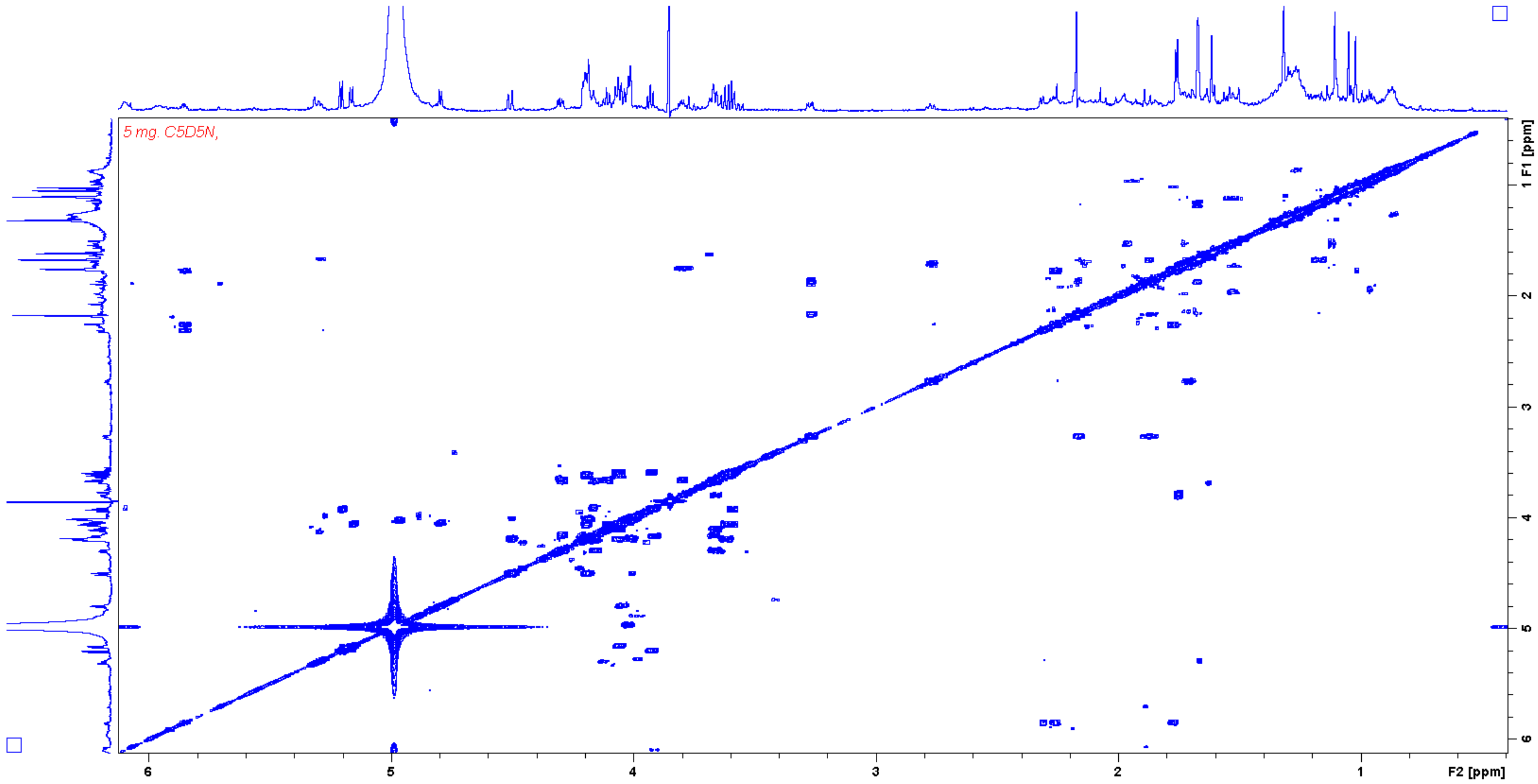

Figure S21. HSQC spectrum of pacificusoside M (**2**) in C_5D_5N.

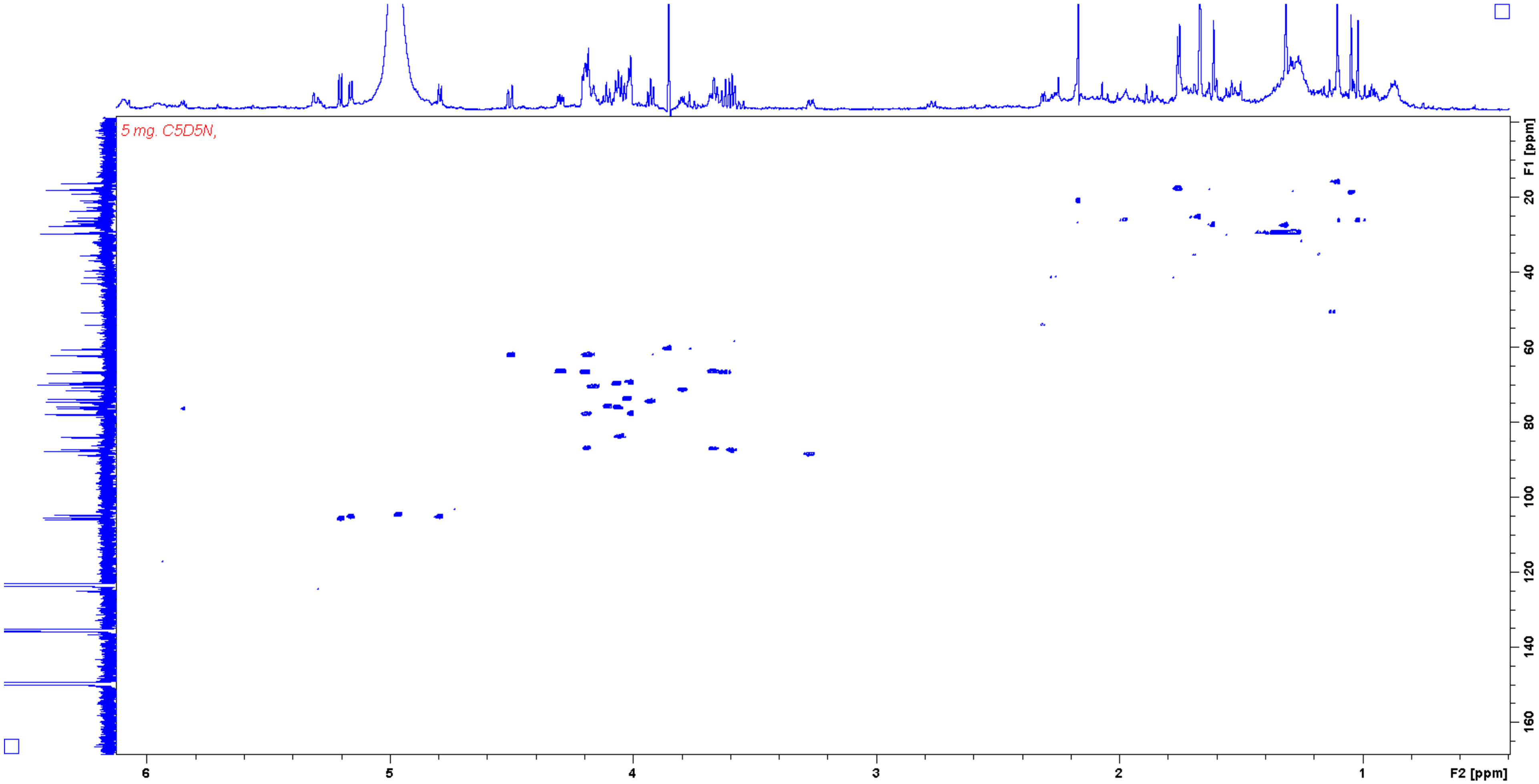

Figure S22. HMBC spectrum of pacificusoside M (**2**) in C_5D_5N.

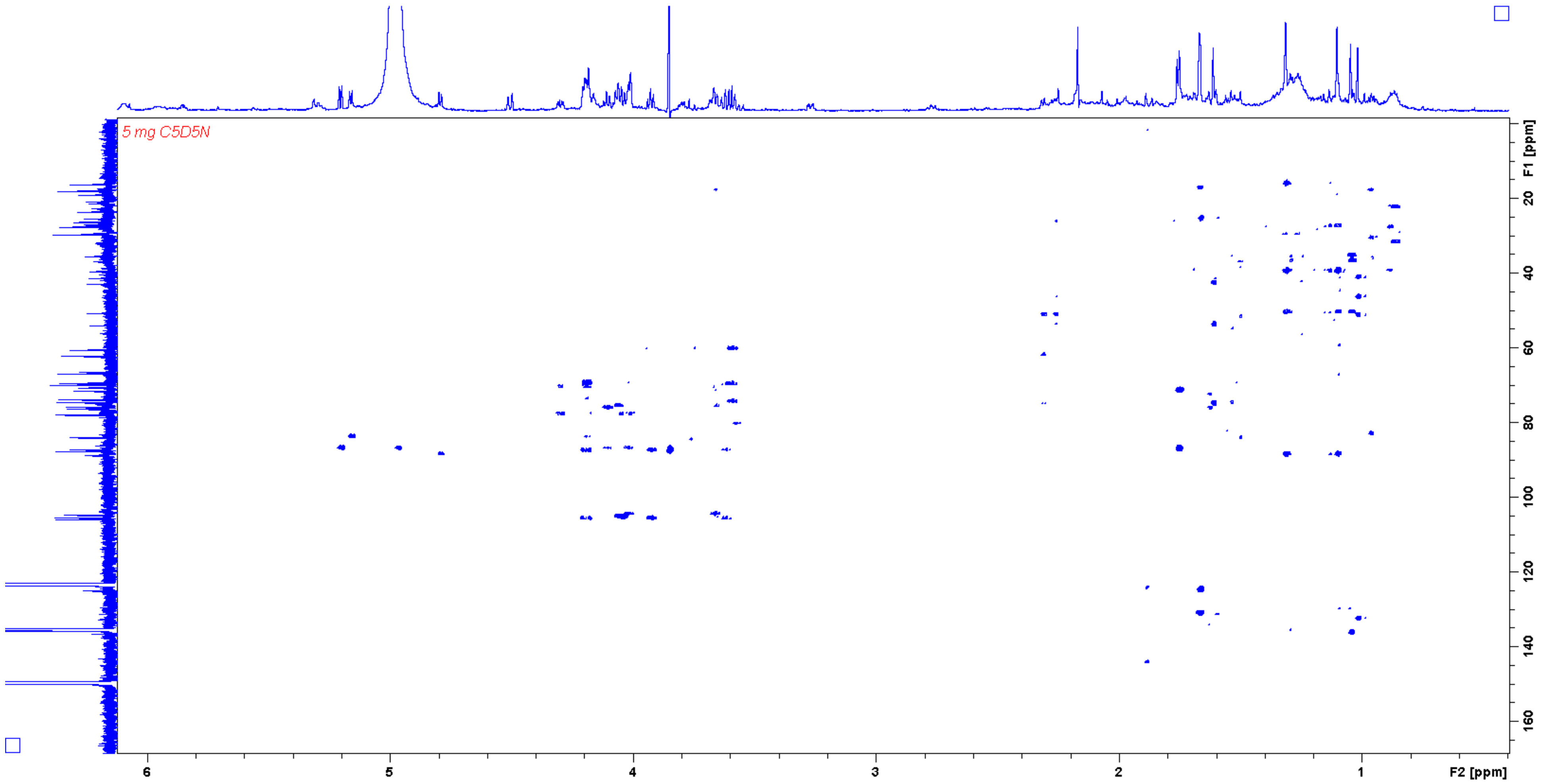

Figure S23. ROESY spectrum of pacificusoside M (**2**) in C_5D_5N.

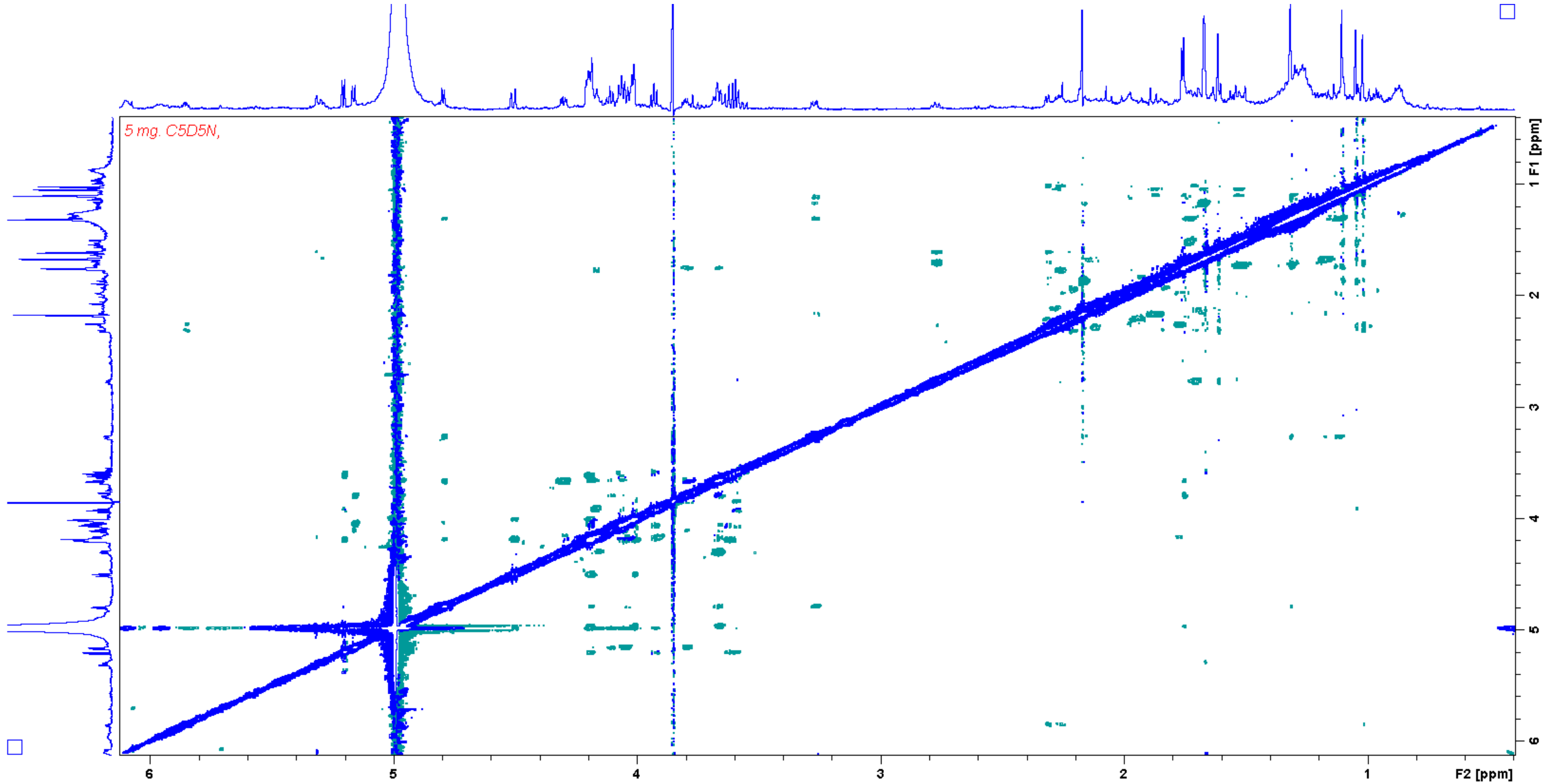

Figure S24. (–)ESIMS/MS spectrum of pacificusoside M (**2**).

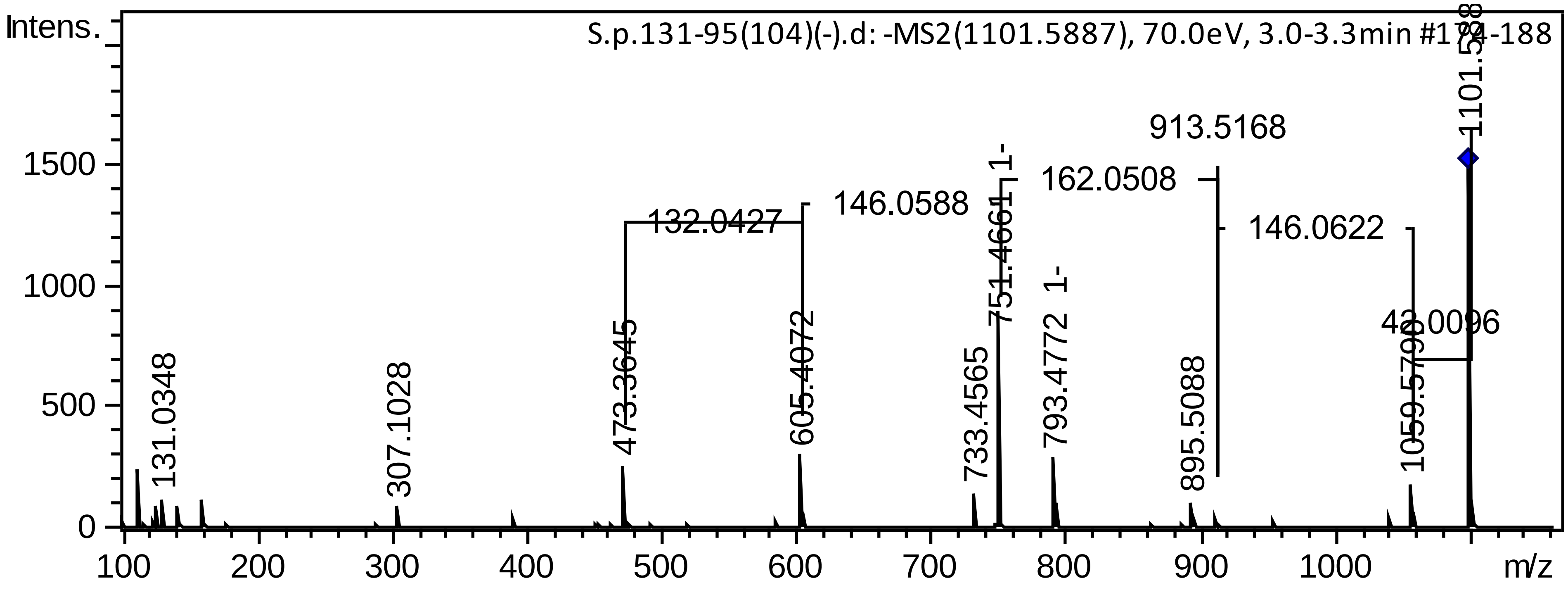

Figure S25. (+)ESIMS/MS spectrum of pacificusoside M (**2**).

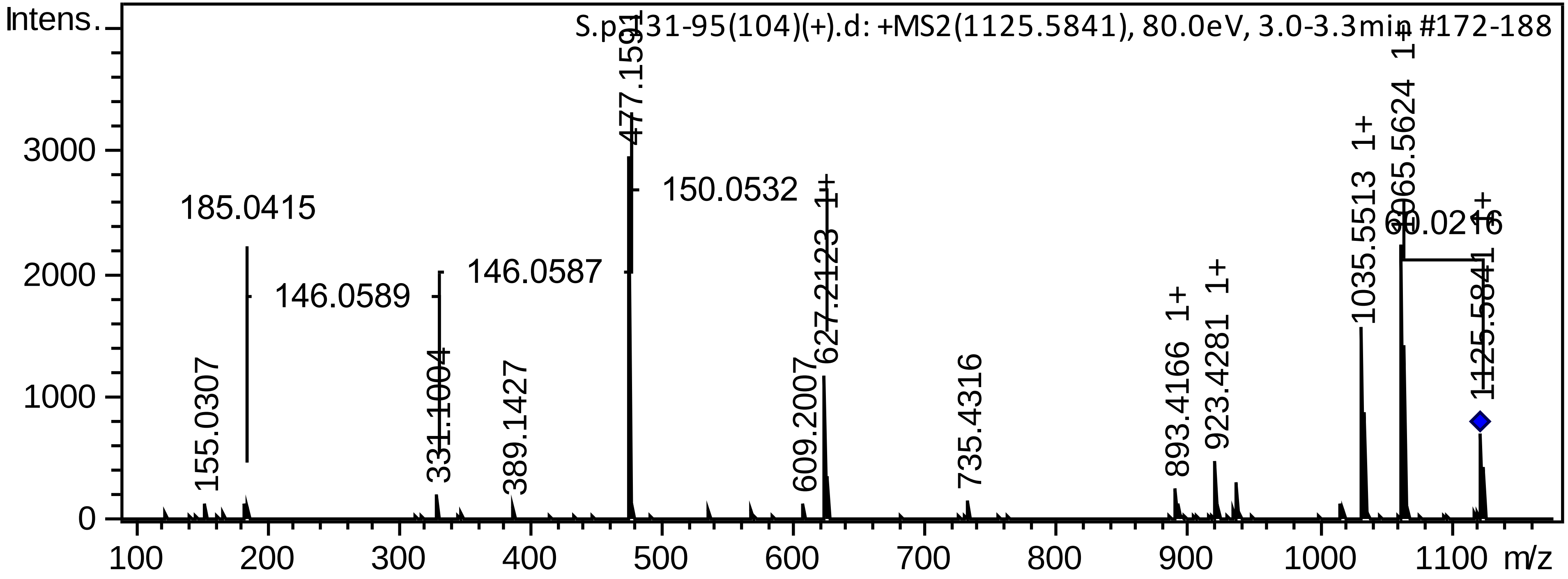

Figure S26. HRESIMS spectrum of pacificusoside N (**3**).

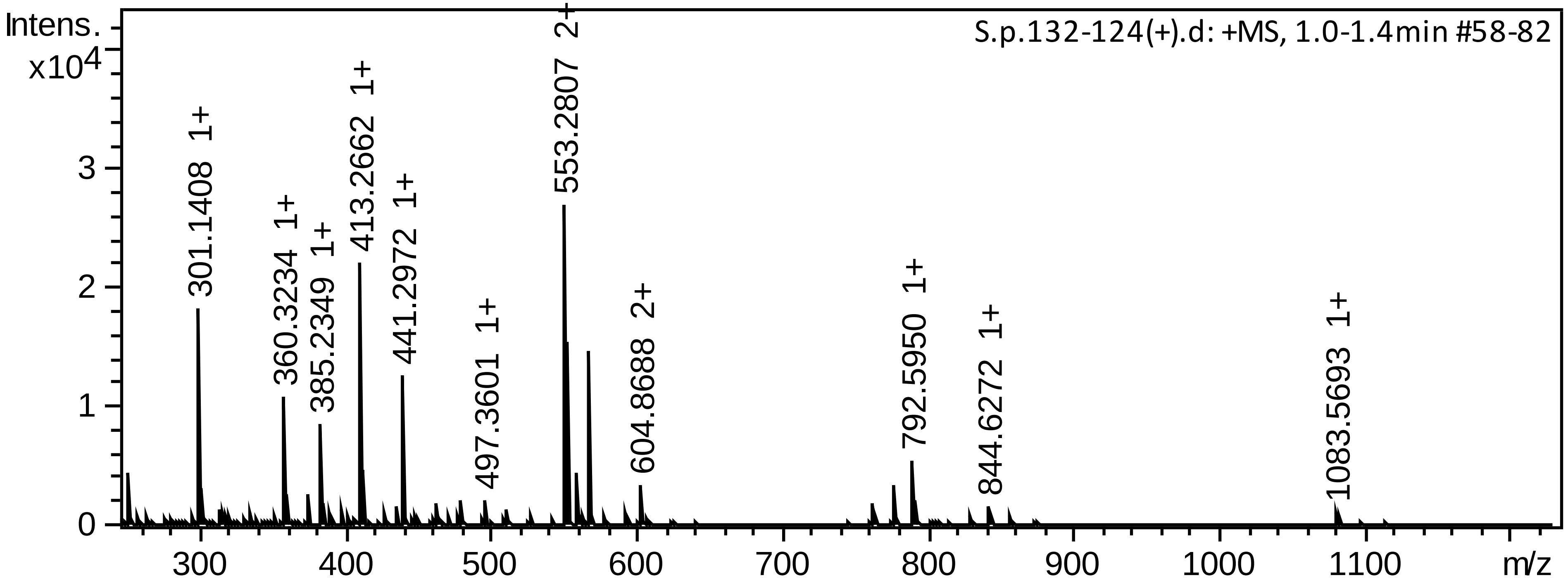

Figure S27. ^{1}H-NMR spectrum of pacificusoside N (**3**) in C_5D_5N.

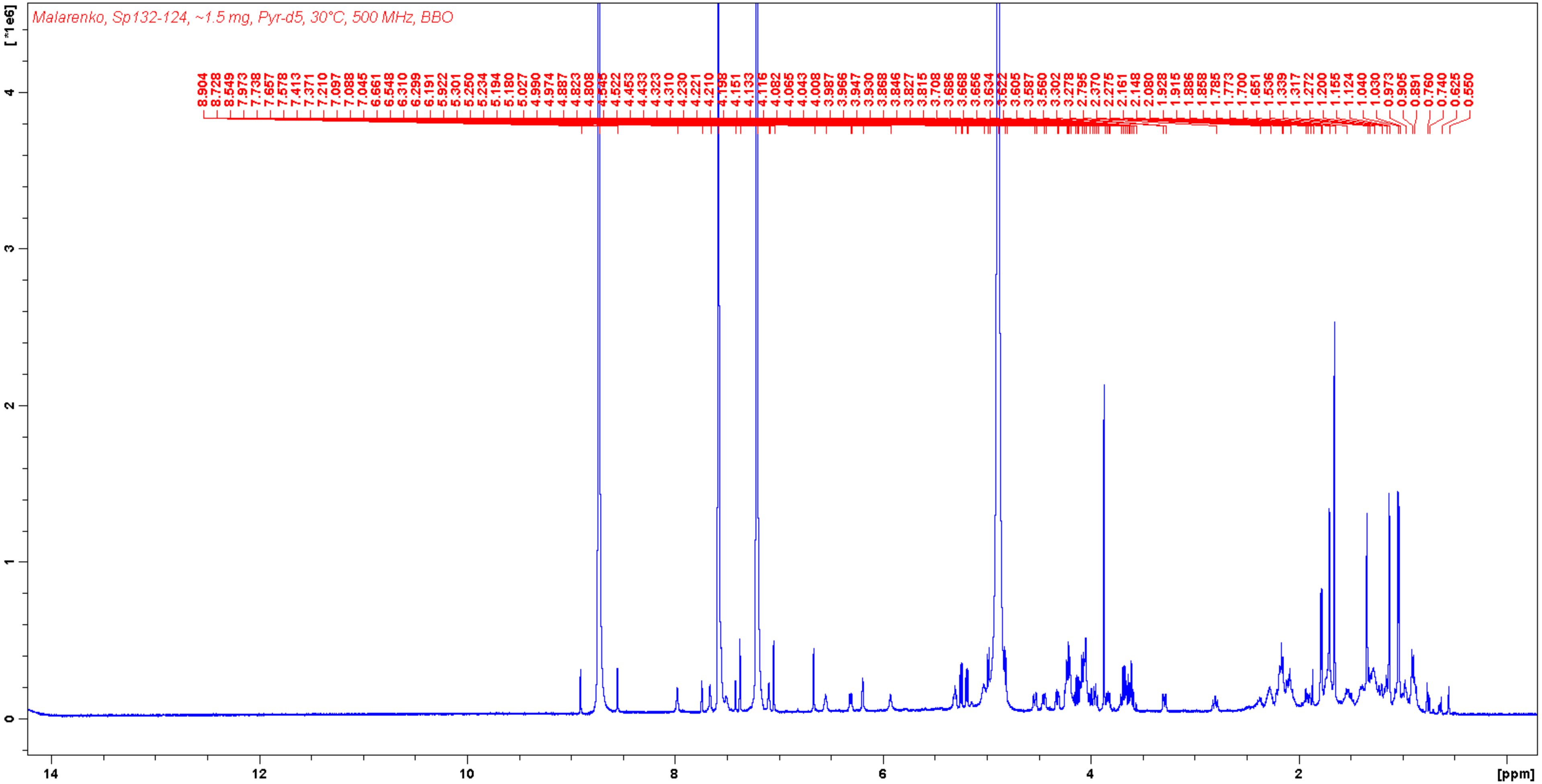

Figure S28. ^{13}C-NMR spectrum of pacificusoside N (**3**) in C_5D_5N.

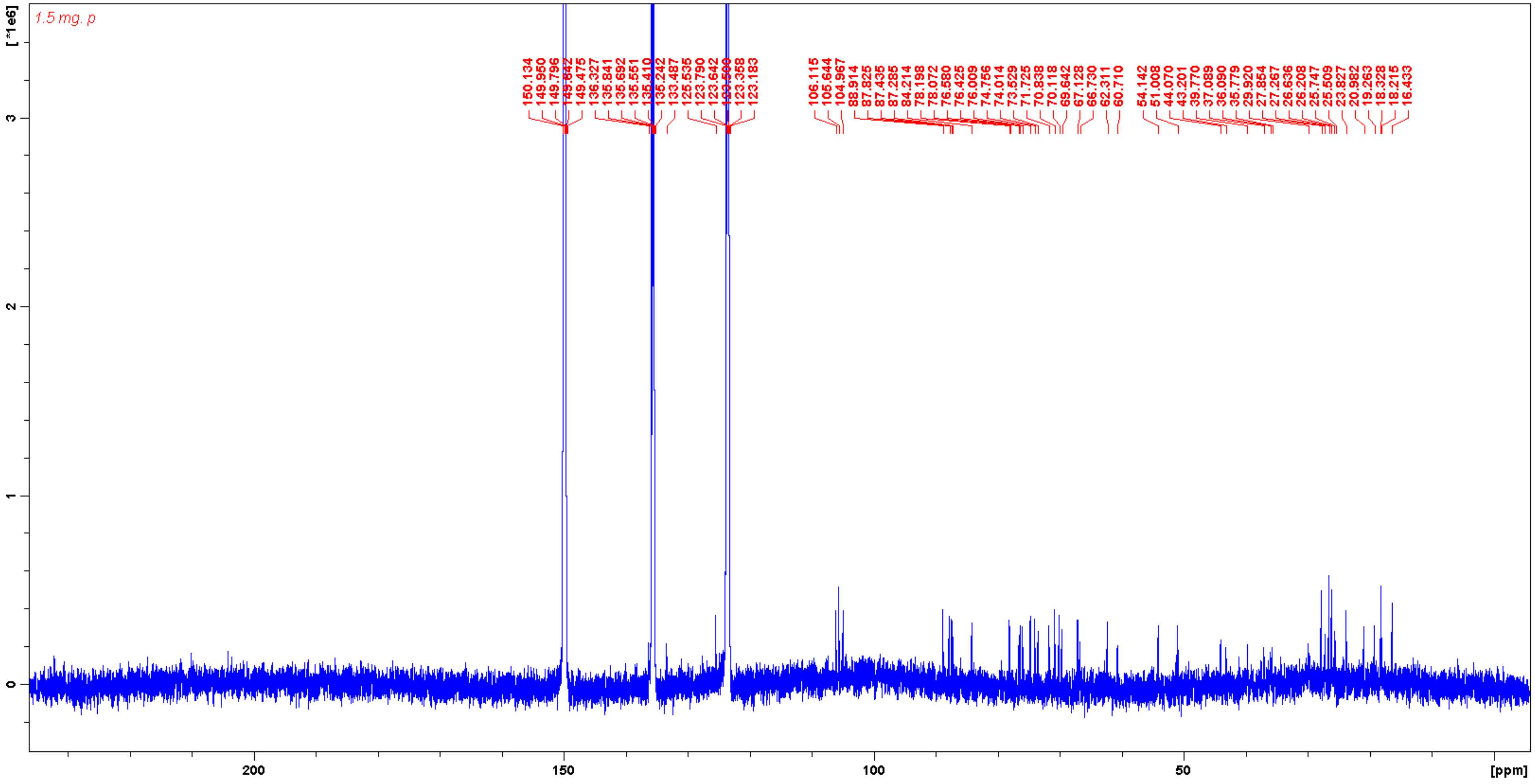

Figure S29. ^{1}H-^{1}H COSY spectrum of pacificusoside N (**3**) in C_5D_5N.

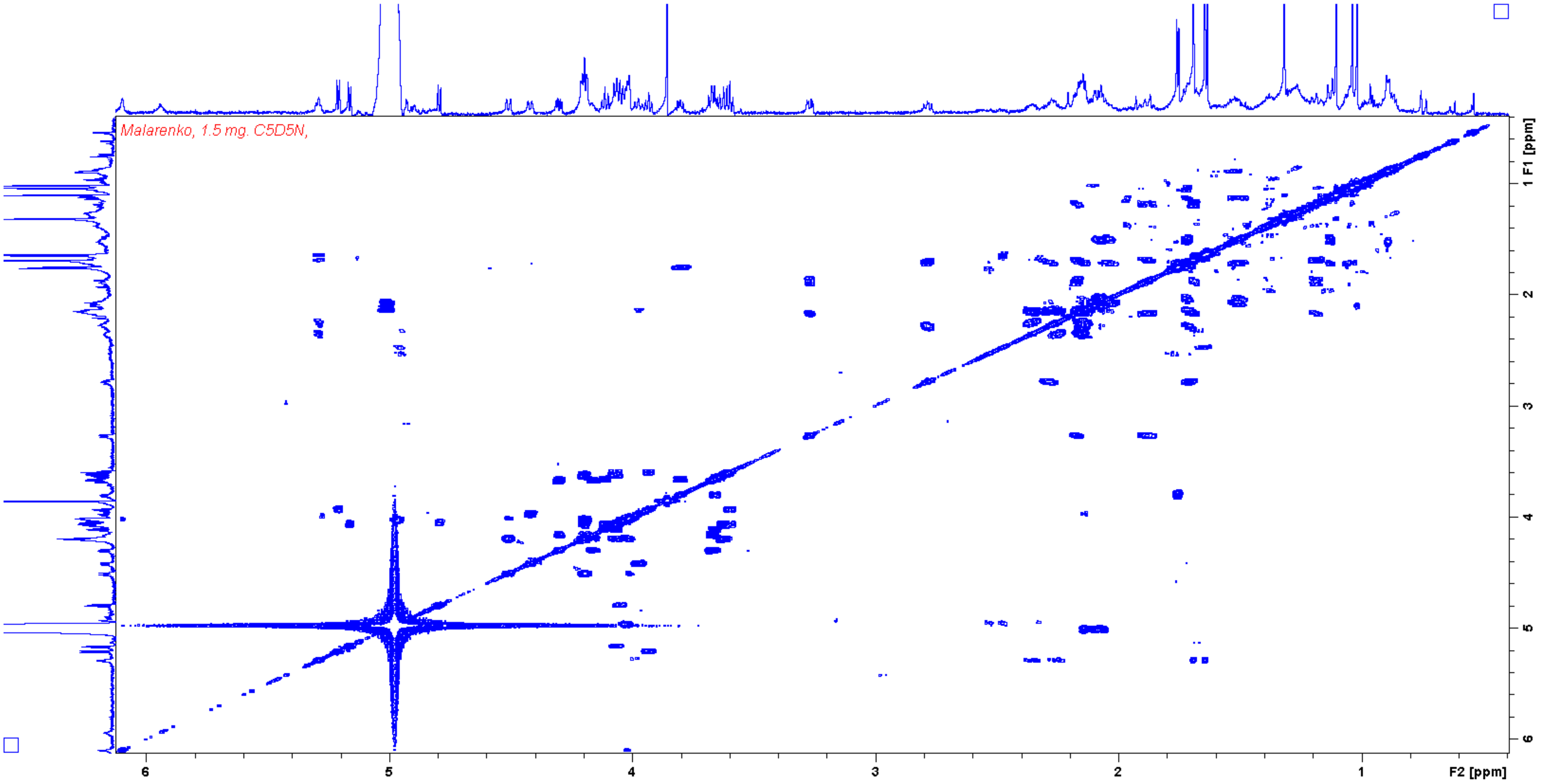

Figure S30. HSQC spectrum of pacificusoside N (**3**) in C_5D_5N.

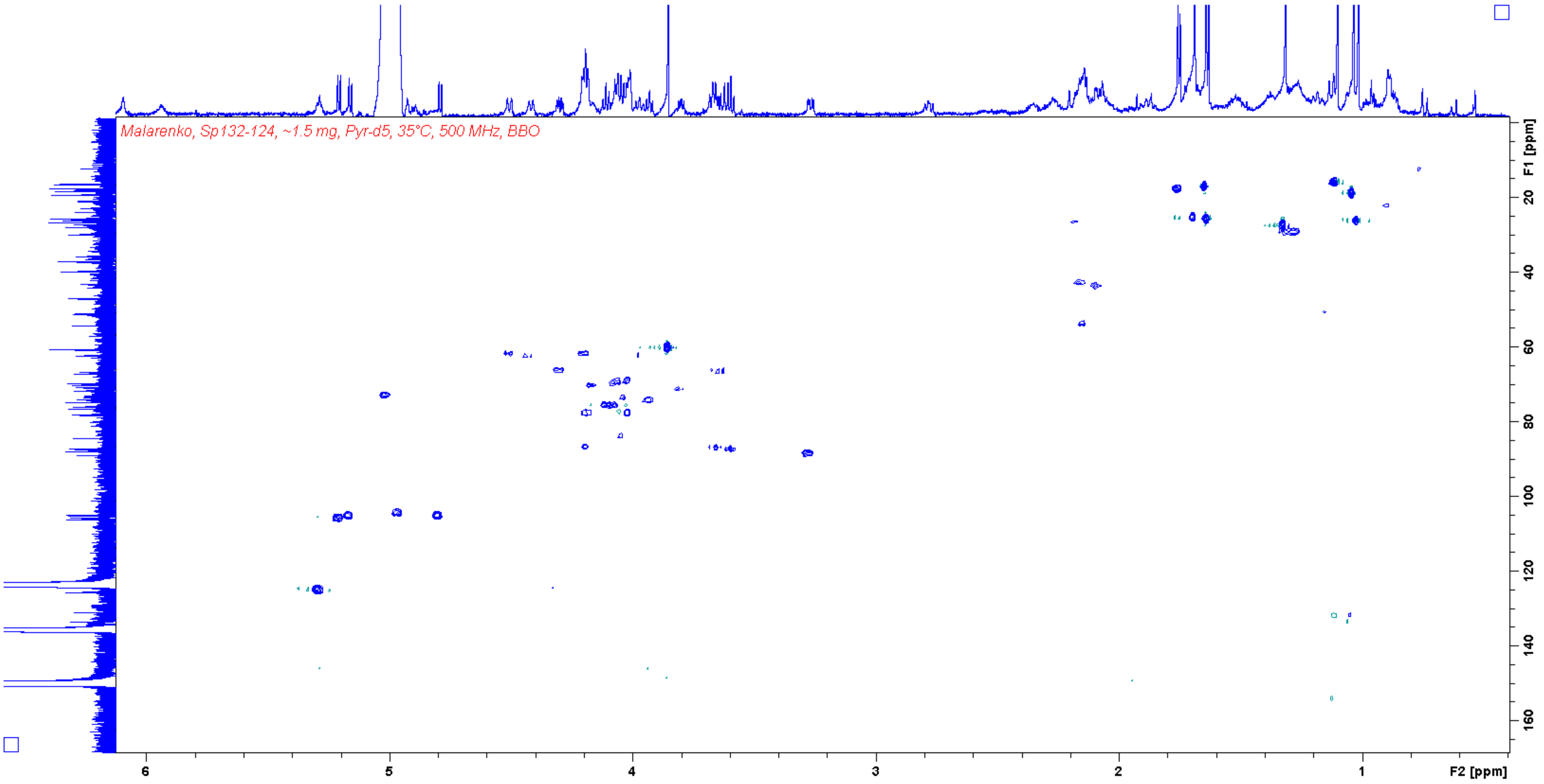

Figure S31. HMBC spectrum of pacificusoside N (**3**) in C_5D_5N.

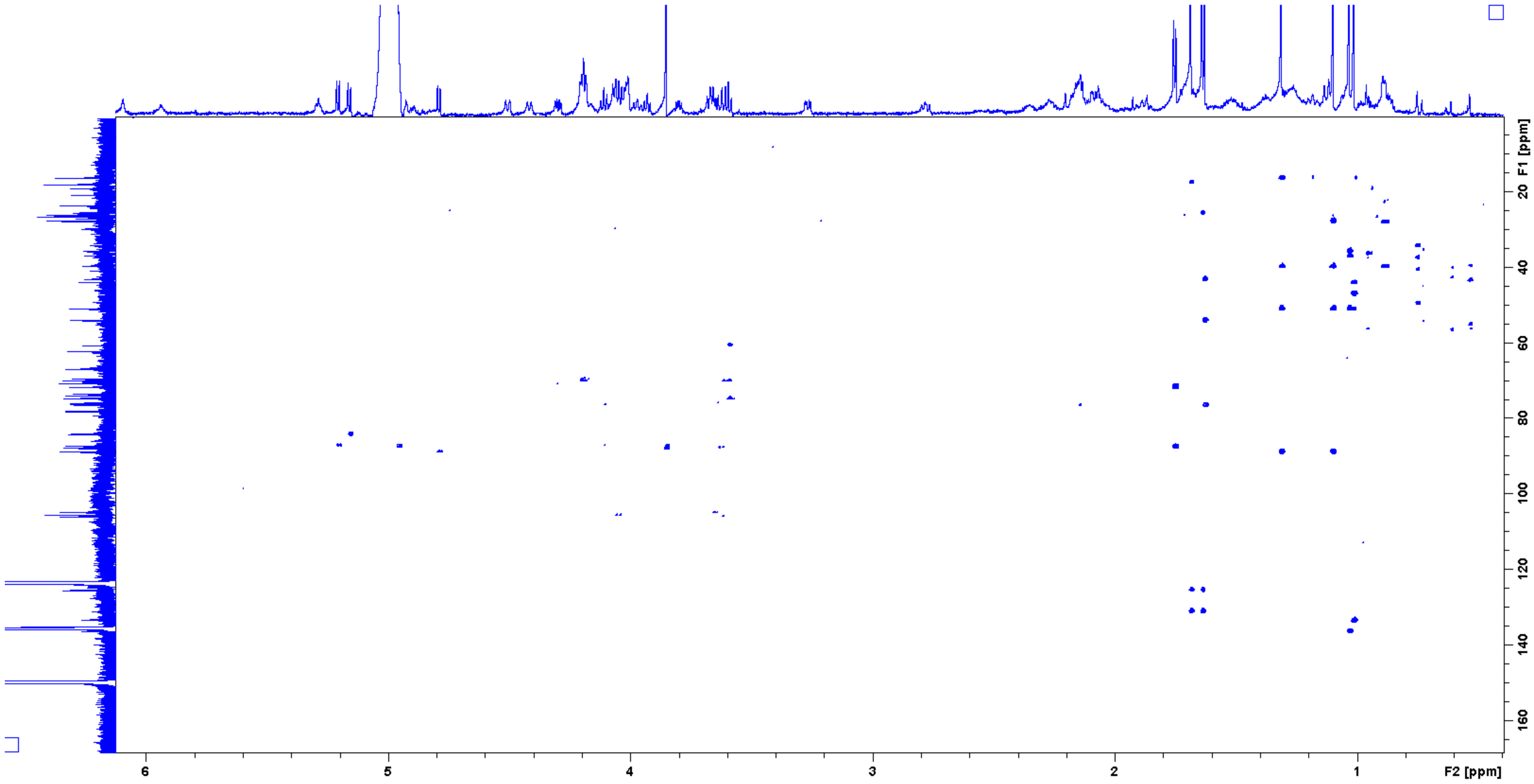

Figure S32. ROESY spectrum of pacificusoside N (**3**) in C_5D_5N.

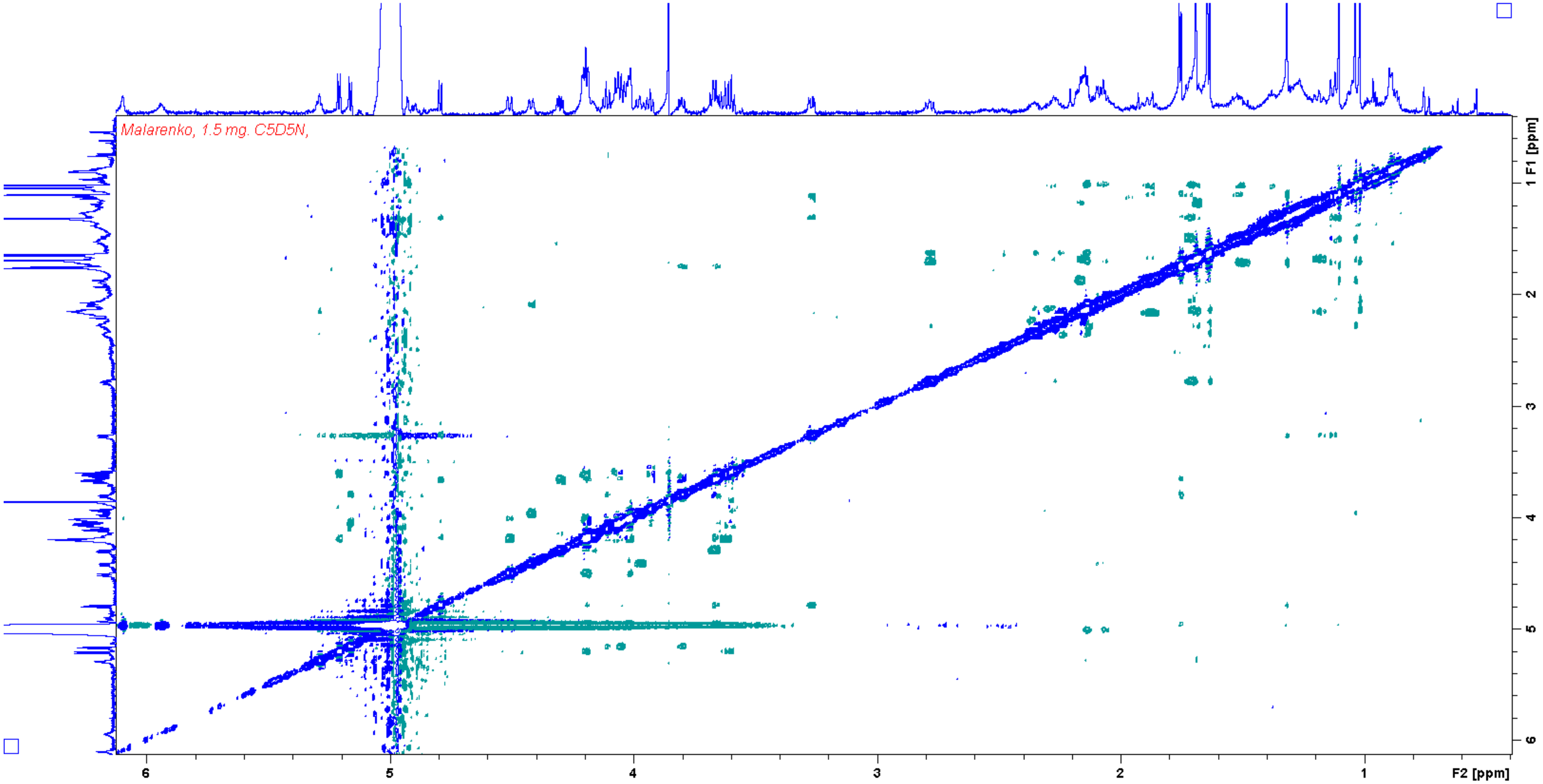

Figure S33. (−)ESIMS/MS spectrum of pacificusoside N (**3**).

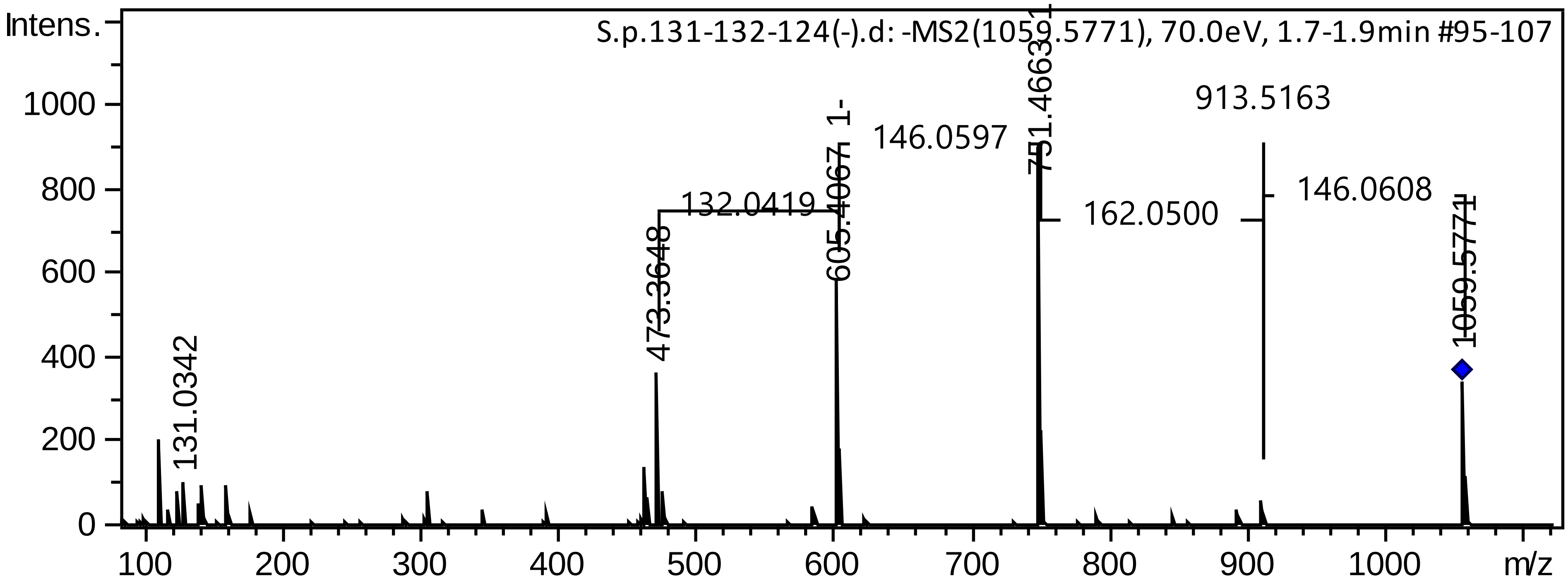

Figure S34. (+)ESIMS/MS spectrum of pacificusoside N (**3**).

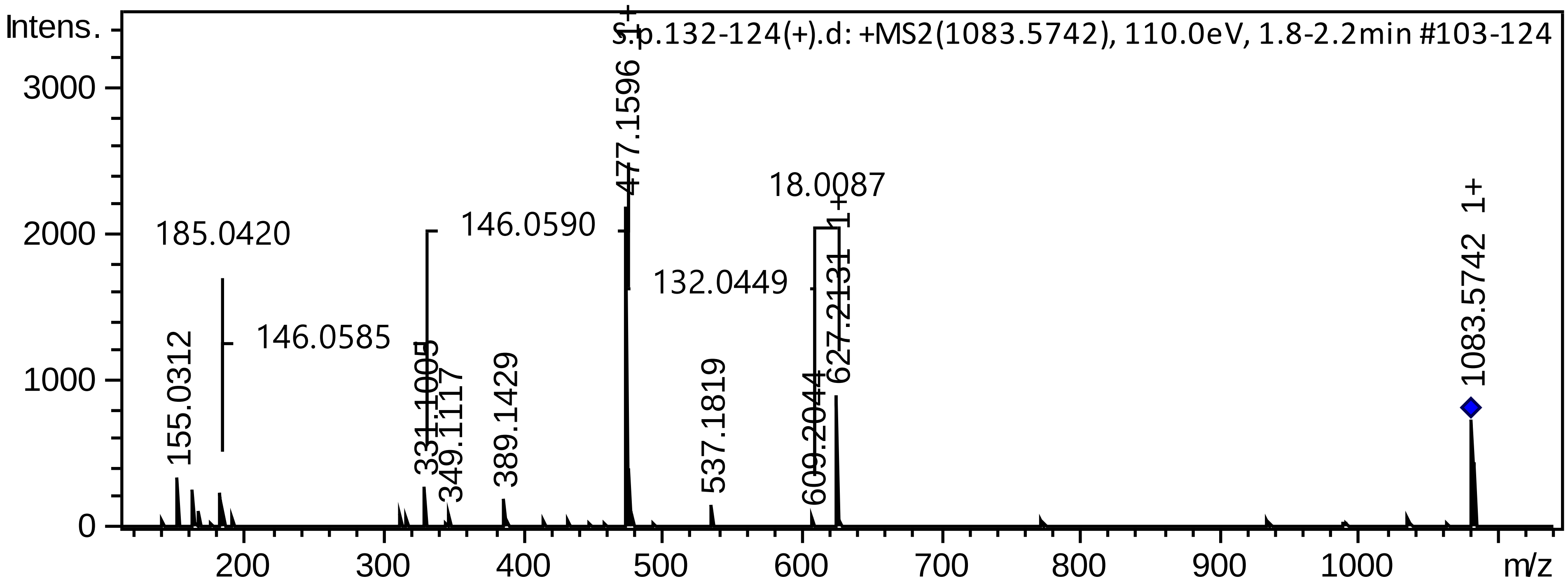

Figure S35. HRESIMS spectrum of pacificusosides O and P (**4** and **5**).

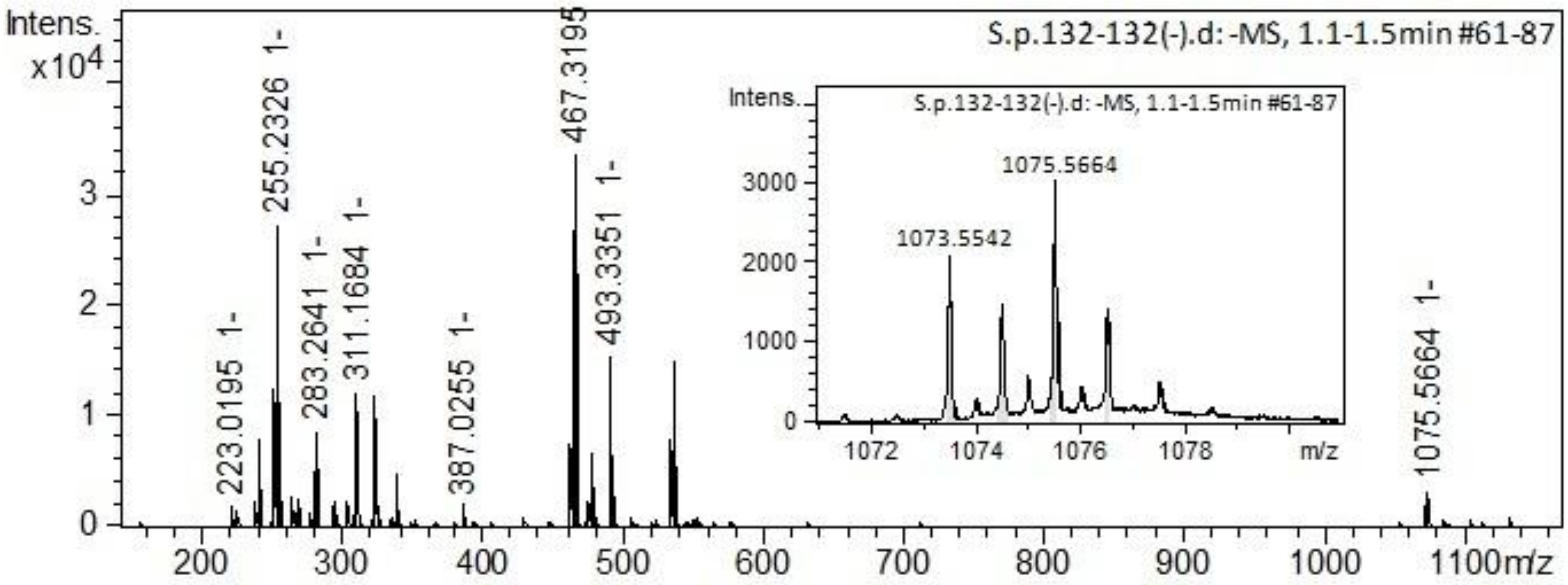

Figure S36. IR spectrum of pacificusosides O and P (**4** and **5**) in KBr (transmittance vs wave number).

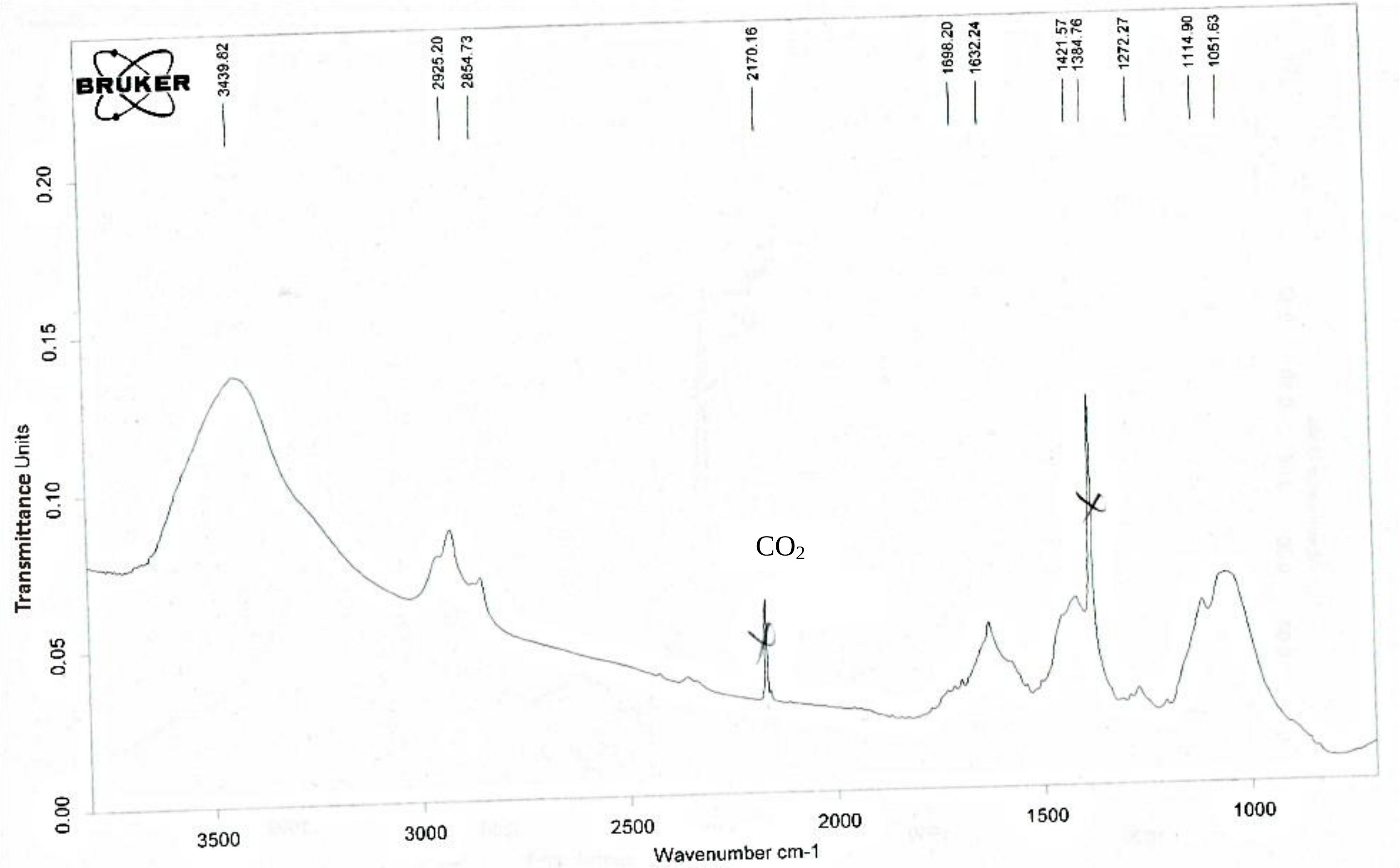

Figure S37. ^{1}H-NMR spectrum of pacificusosides O and P (**4** and **5**) in C_5D_5N.

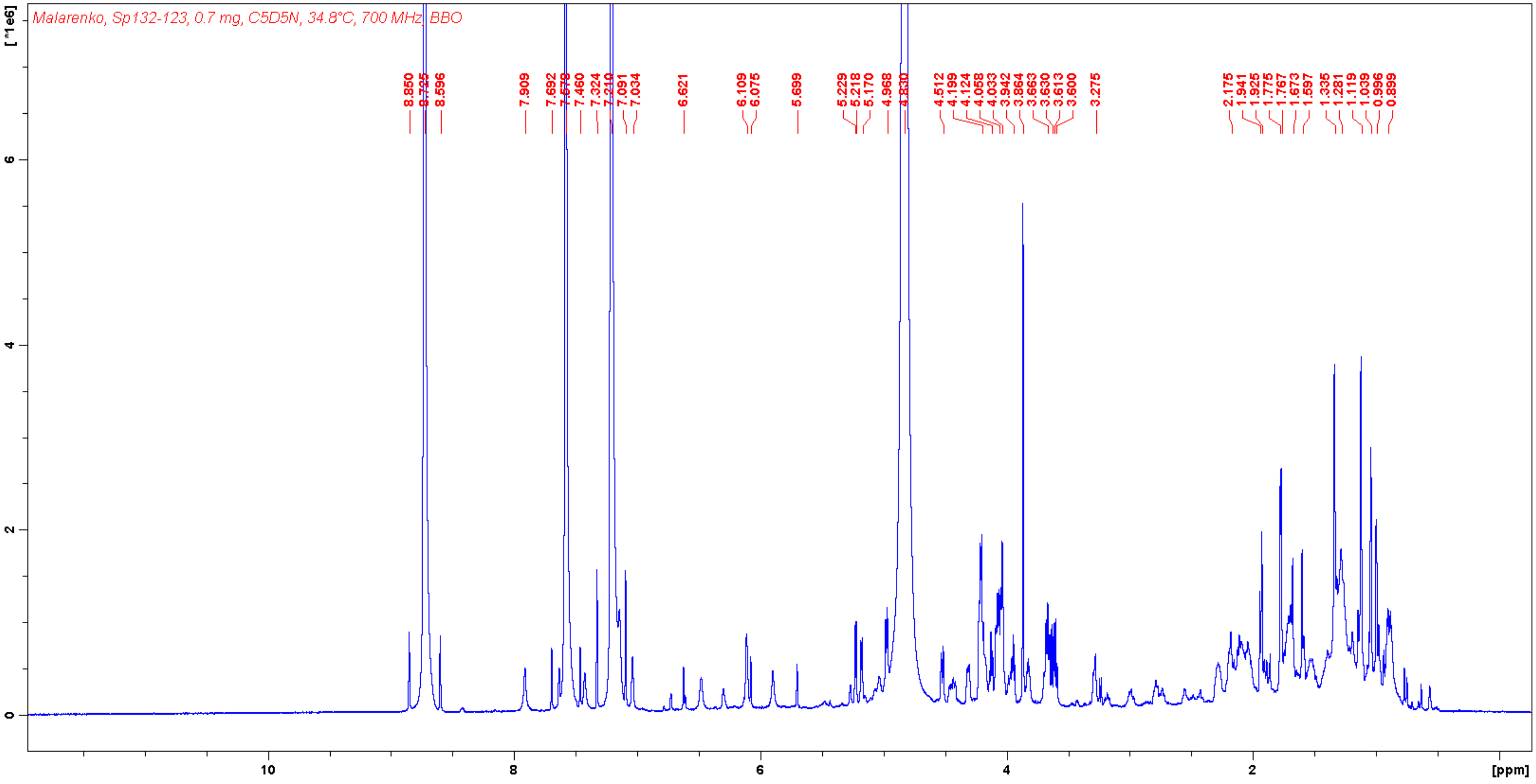

Figure S38. ^{13}C-NMR spectrum of pacificusosides O and P (**4** and **5**) in C_5D_5N.

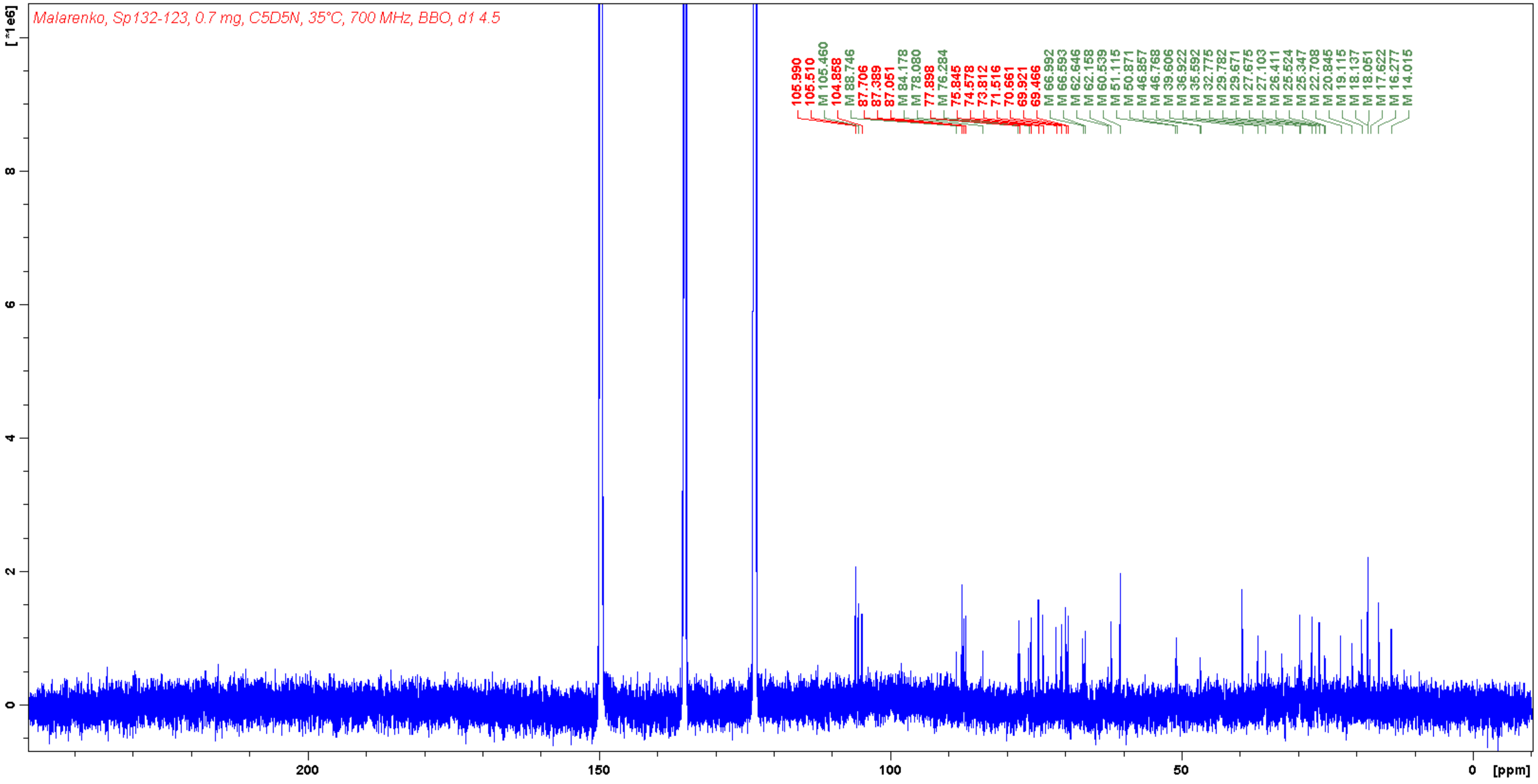

Figure S39. ^{1}H-^{1}H COSY spectrum of pacificusosides O and P (**4** and **5**) in C_5D_5N.

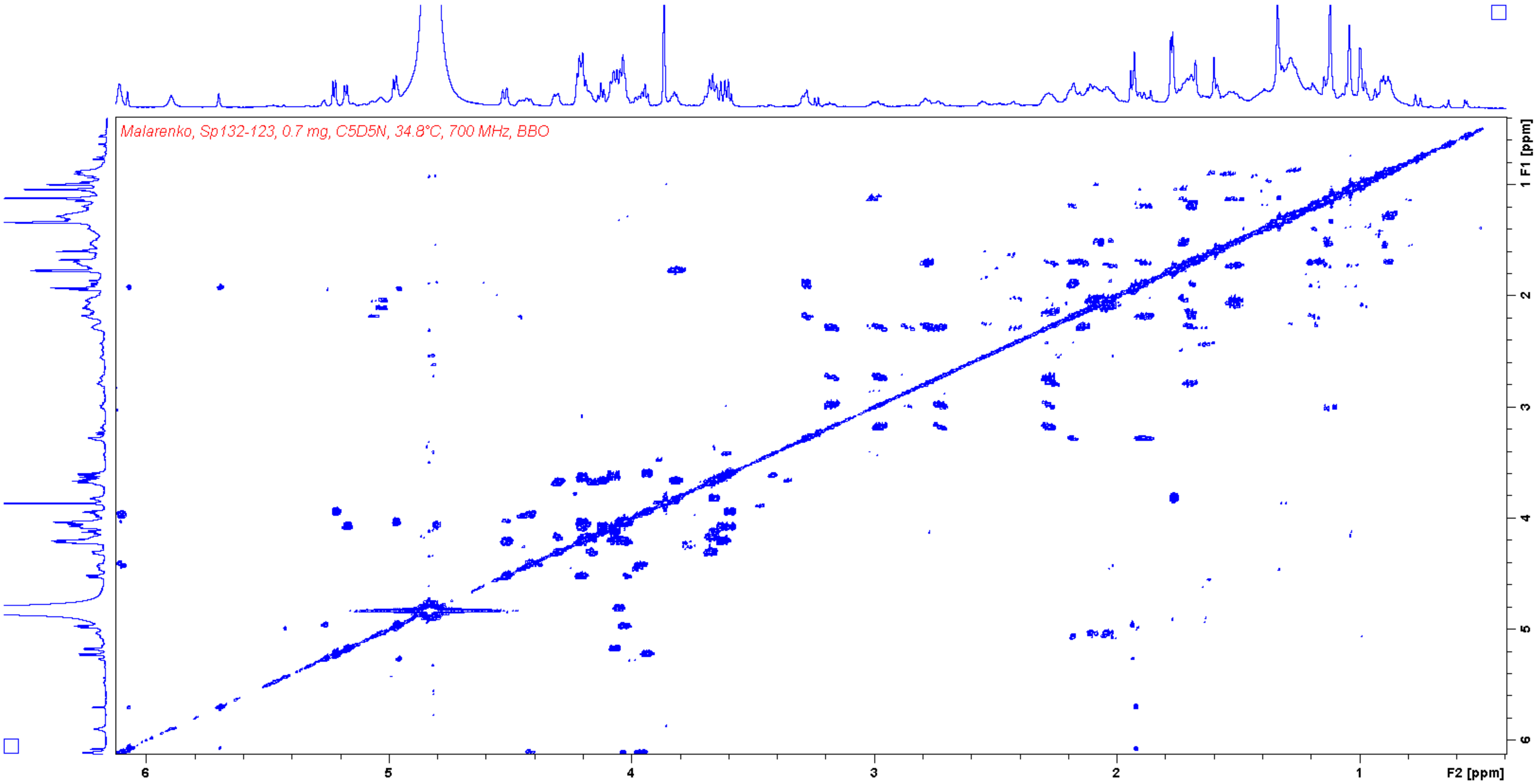

Figure S40. HSQC spectrum of pacificusosides O and P (**4** and **5**) in C_5D_5N.

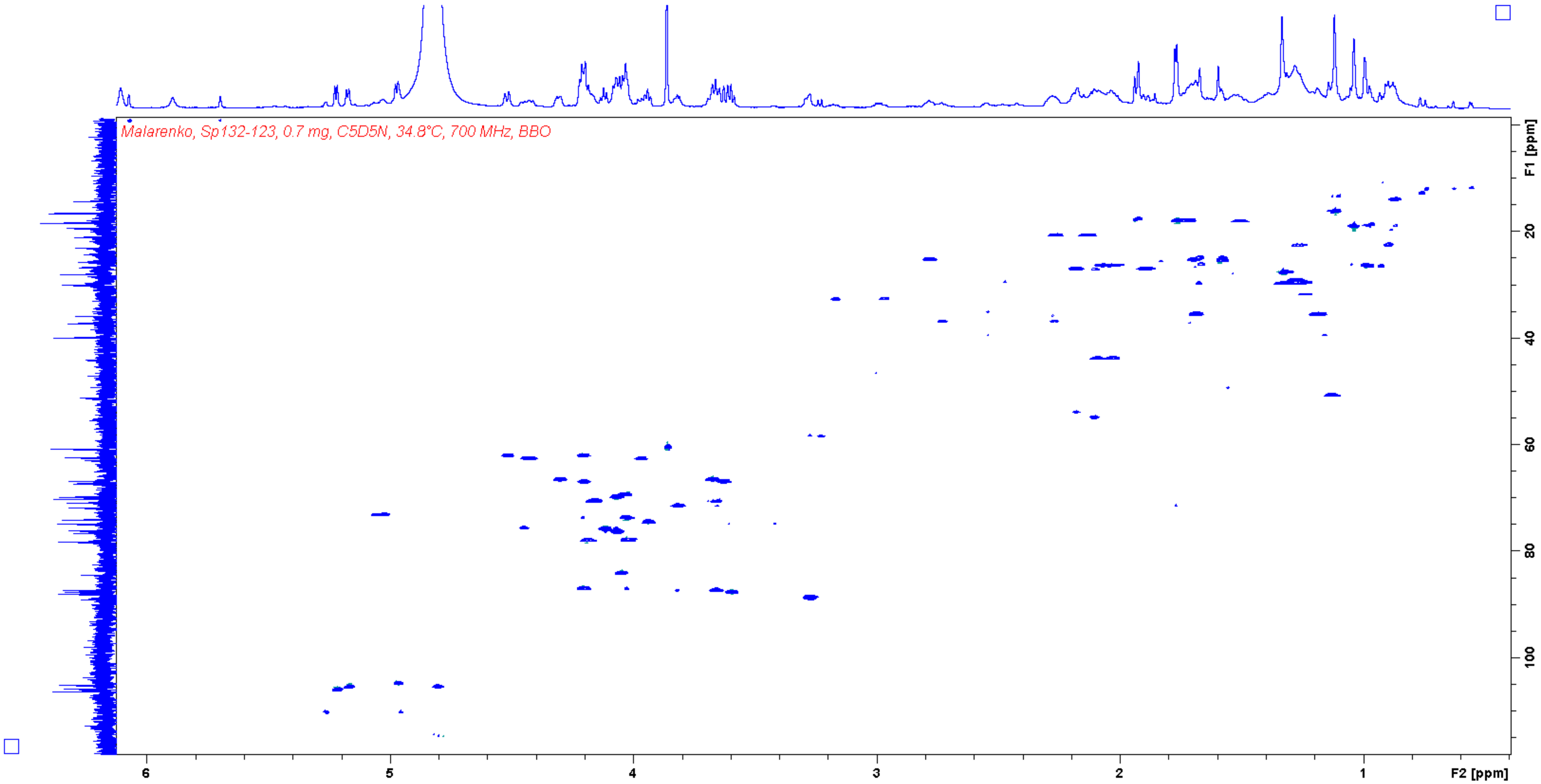

Figure S41. HMBC spectrum of pacificusosides O and P (**4** and **5**) in C_5D_5N.

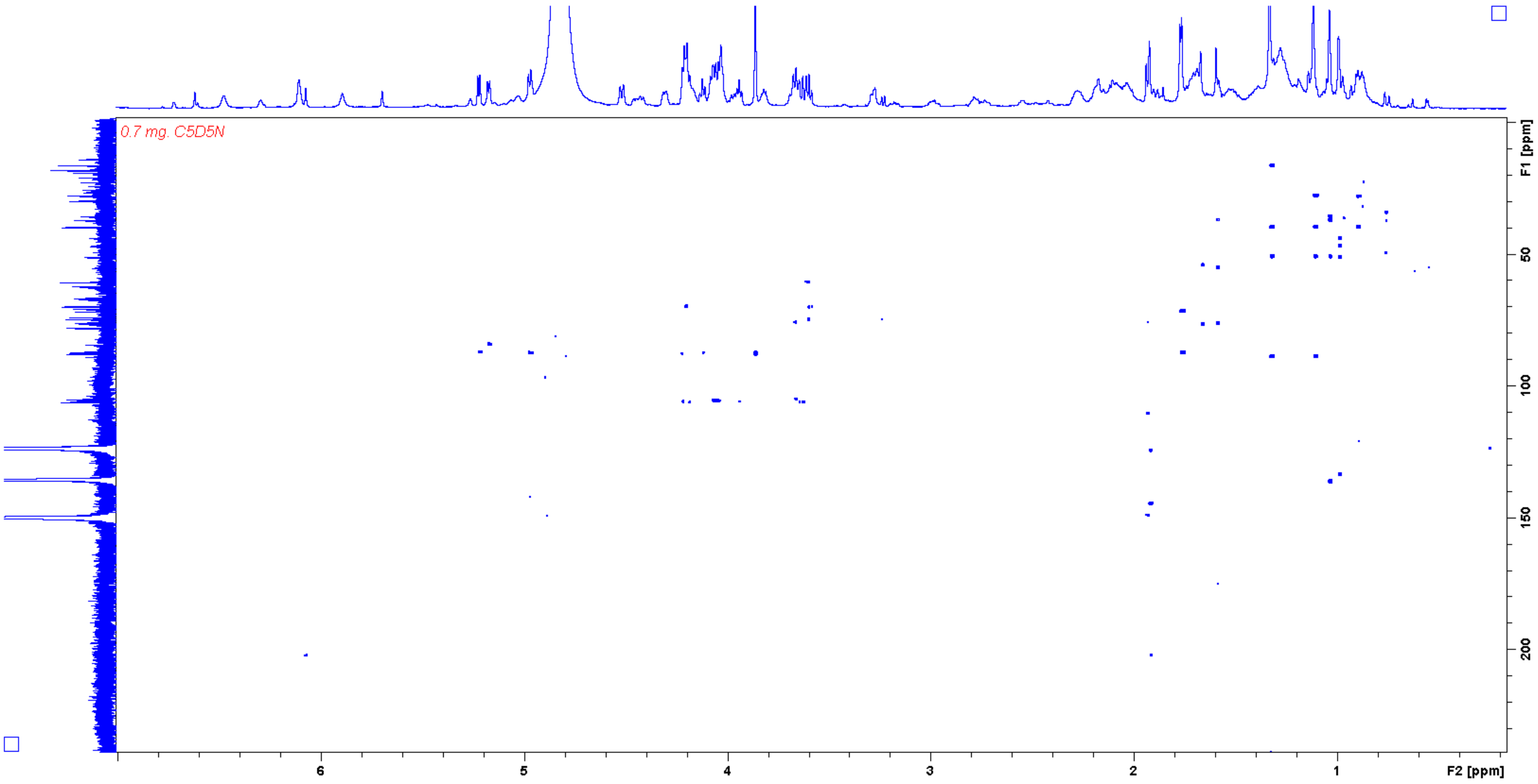

Figure S42. ROESY spectrum of pacificusosides O and P (**4** and **5**) in C_5D_5N.

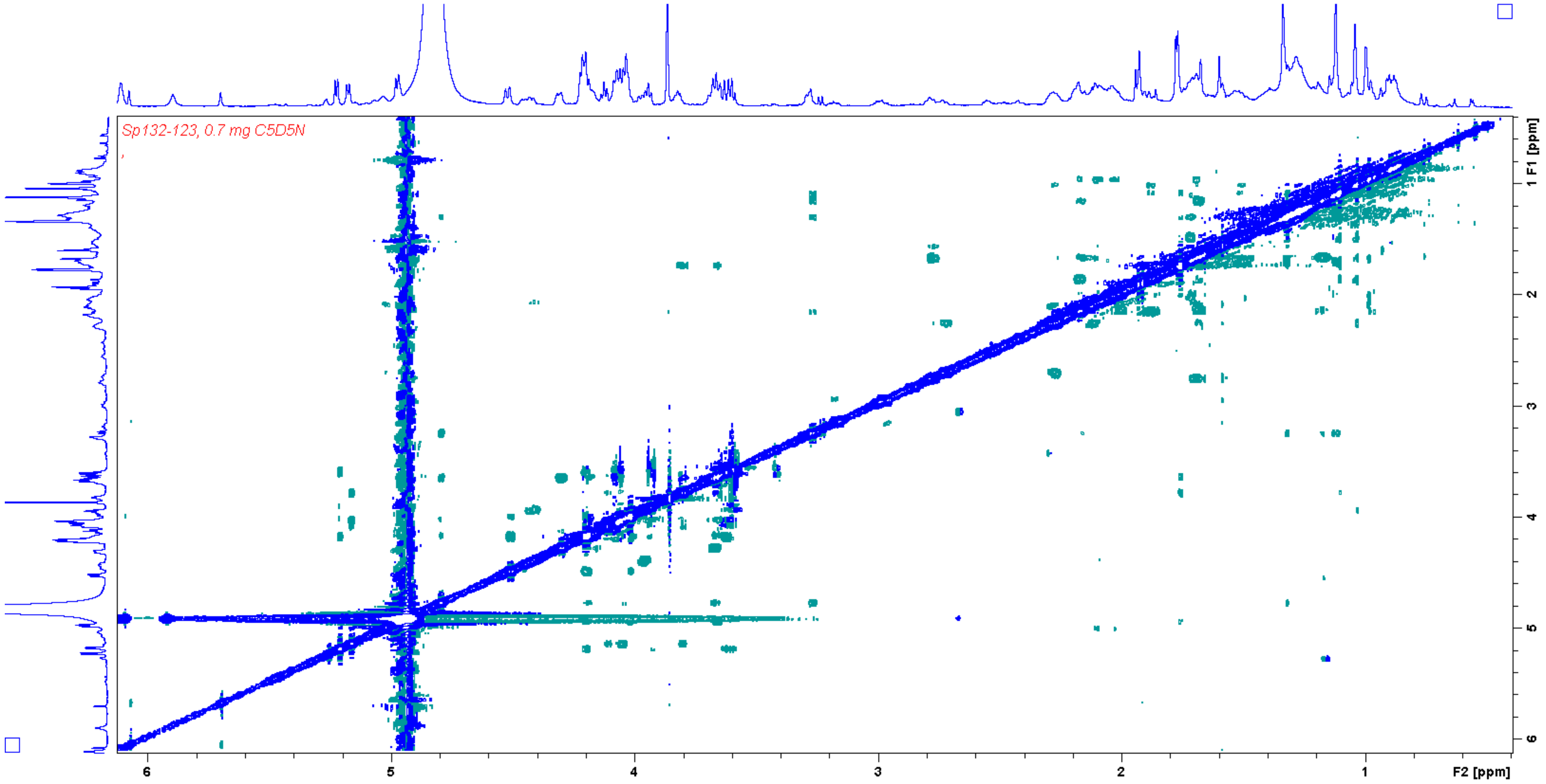

Figure S43. (–)ESIMS/MS spectrum of pacificusoside O and P (**4** and **5**).

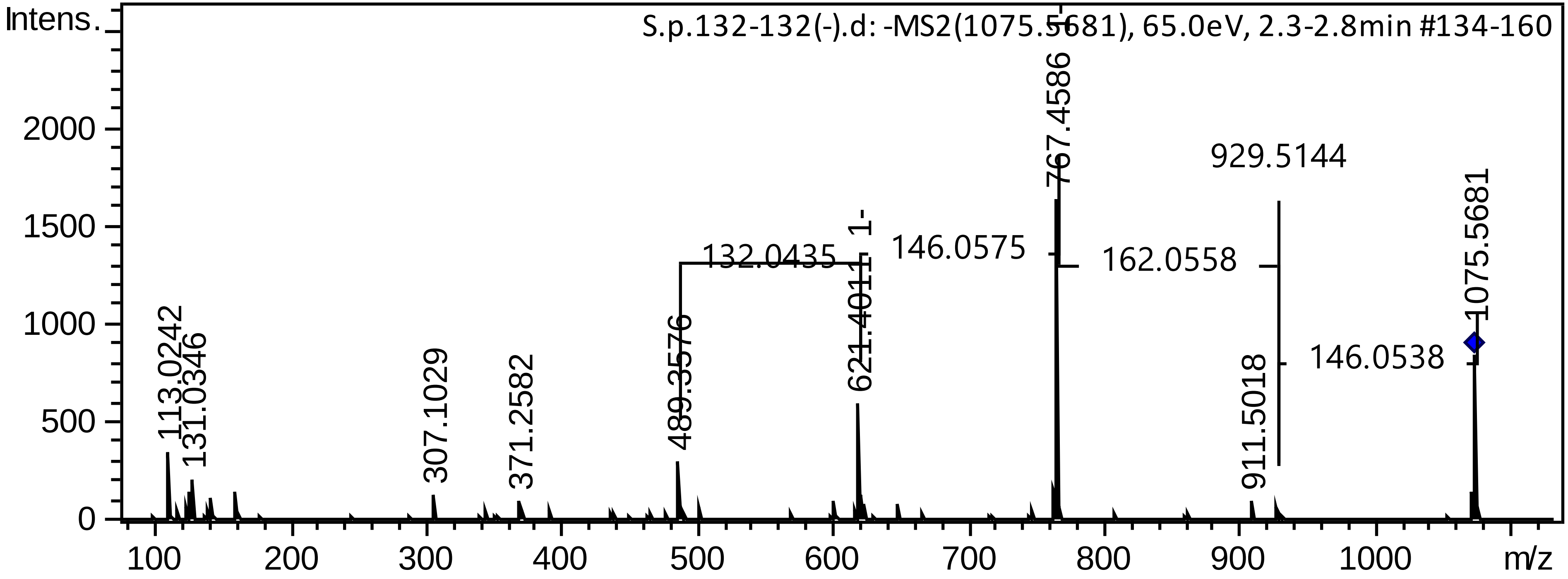

Figure S44. (+)ESIMS/MS spectrum of pacificusoside O and P (**4** and **5**).

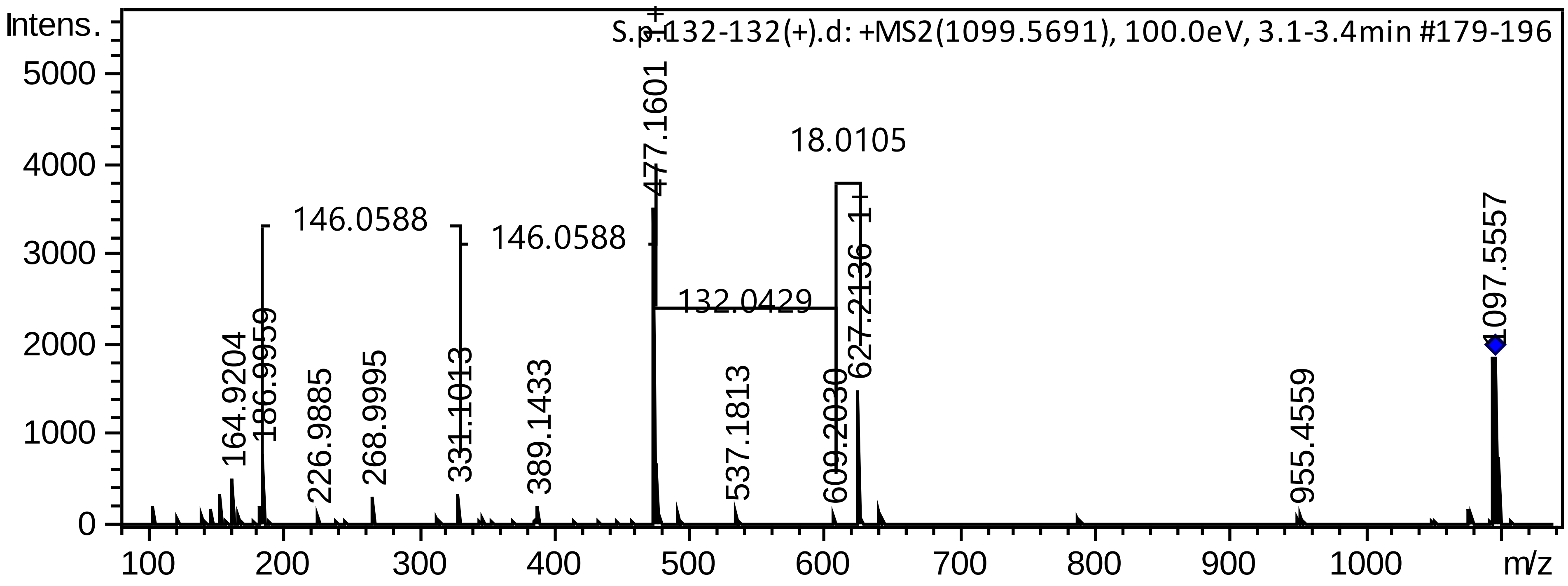

Figure S45. HRESIMS spectrum of pacificusoside Q (**6**).

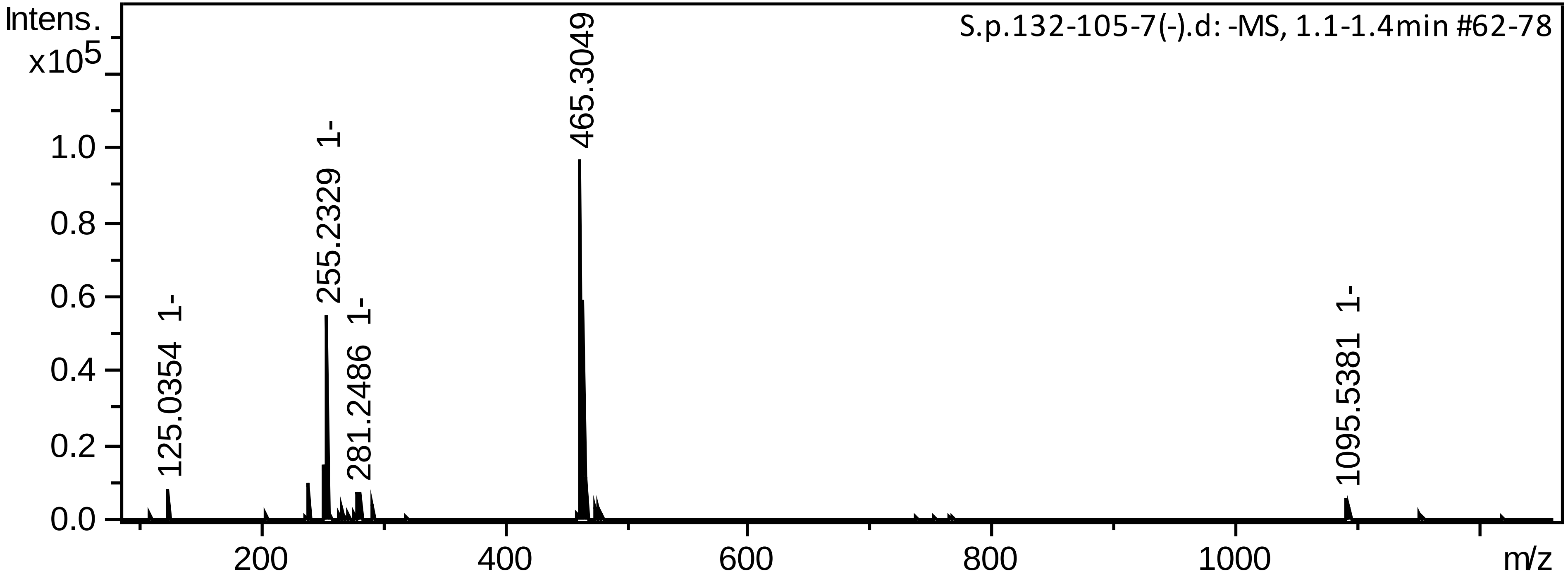

Figure S46. ^{1}H-NMR spectrum of pacificusoside Q (**6**) in C_5D_5N.

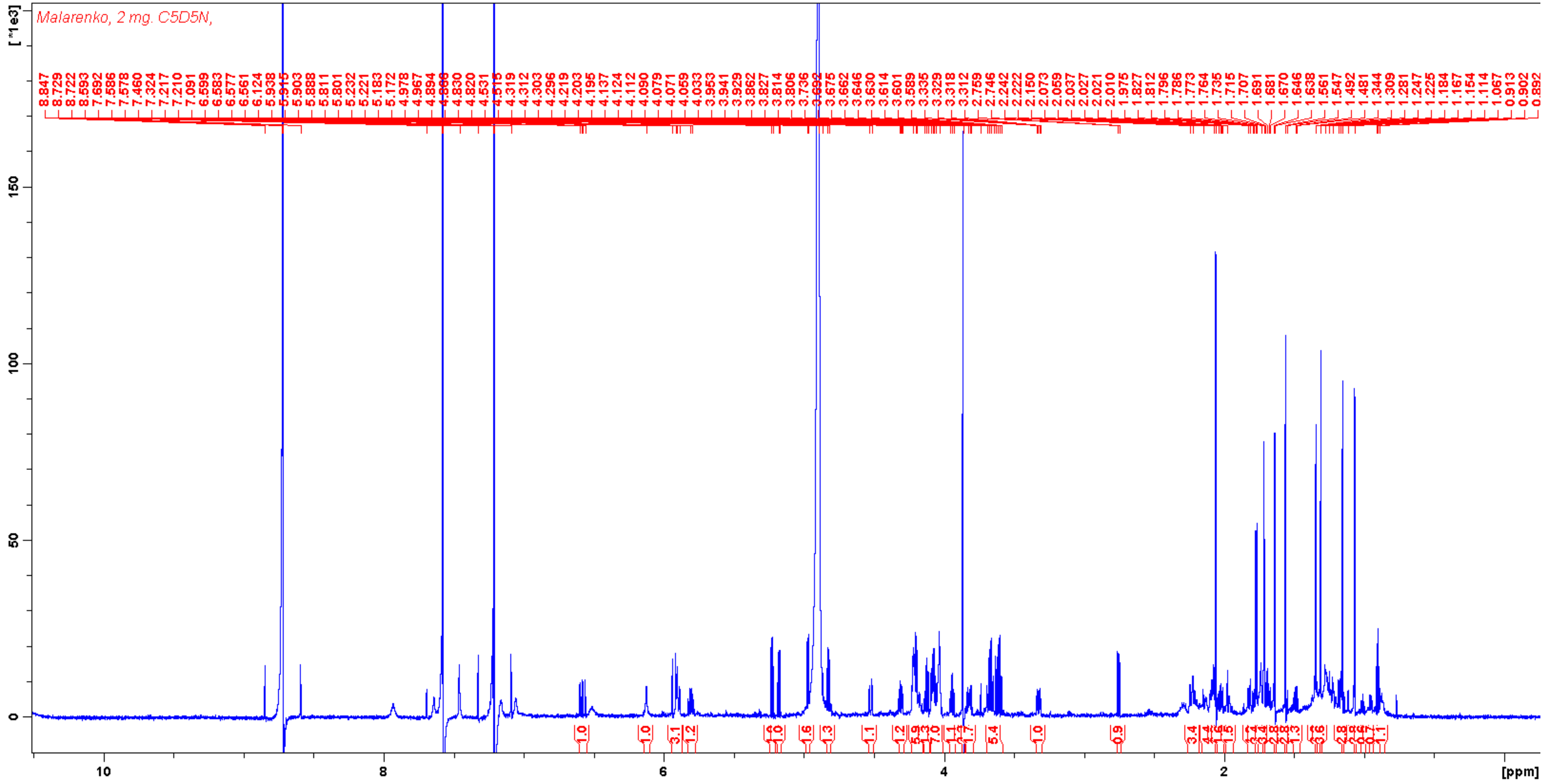

Figure S47. ^{13}C-NMR spectrum of pacificusoside Q (**6**) in C_5D_5N.

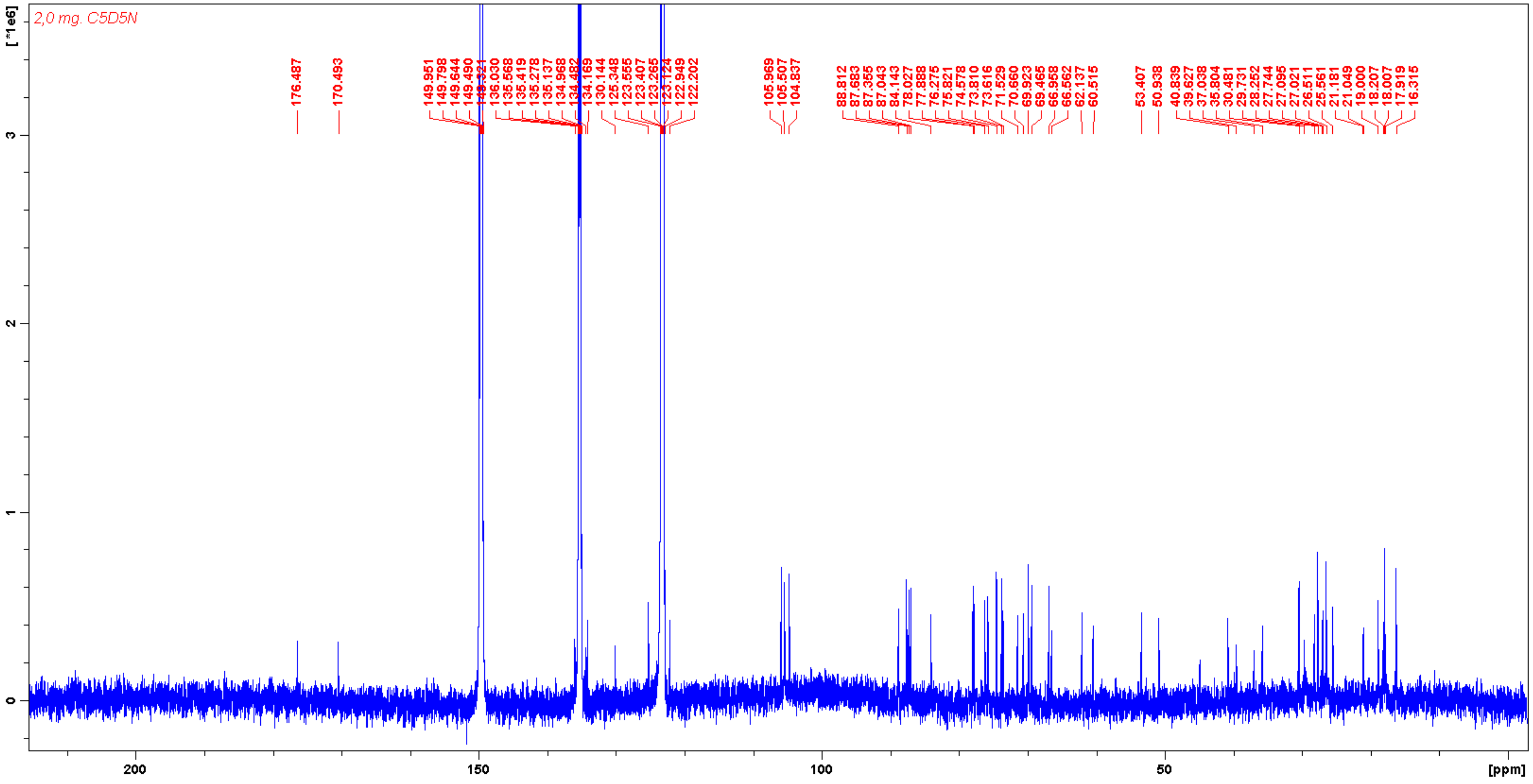

Figure S48. ^{1}H-^{1}H COSY spectrum of pacificusoside Q (**6**) in C_5D_5N.

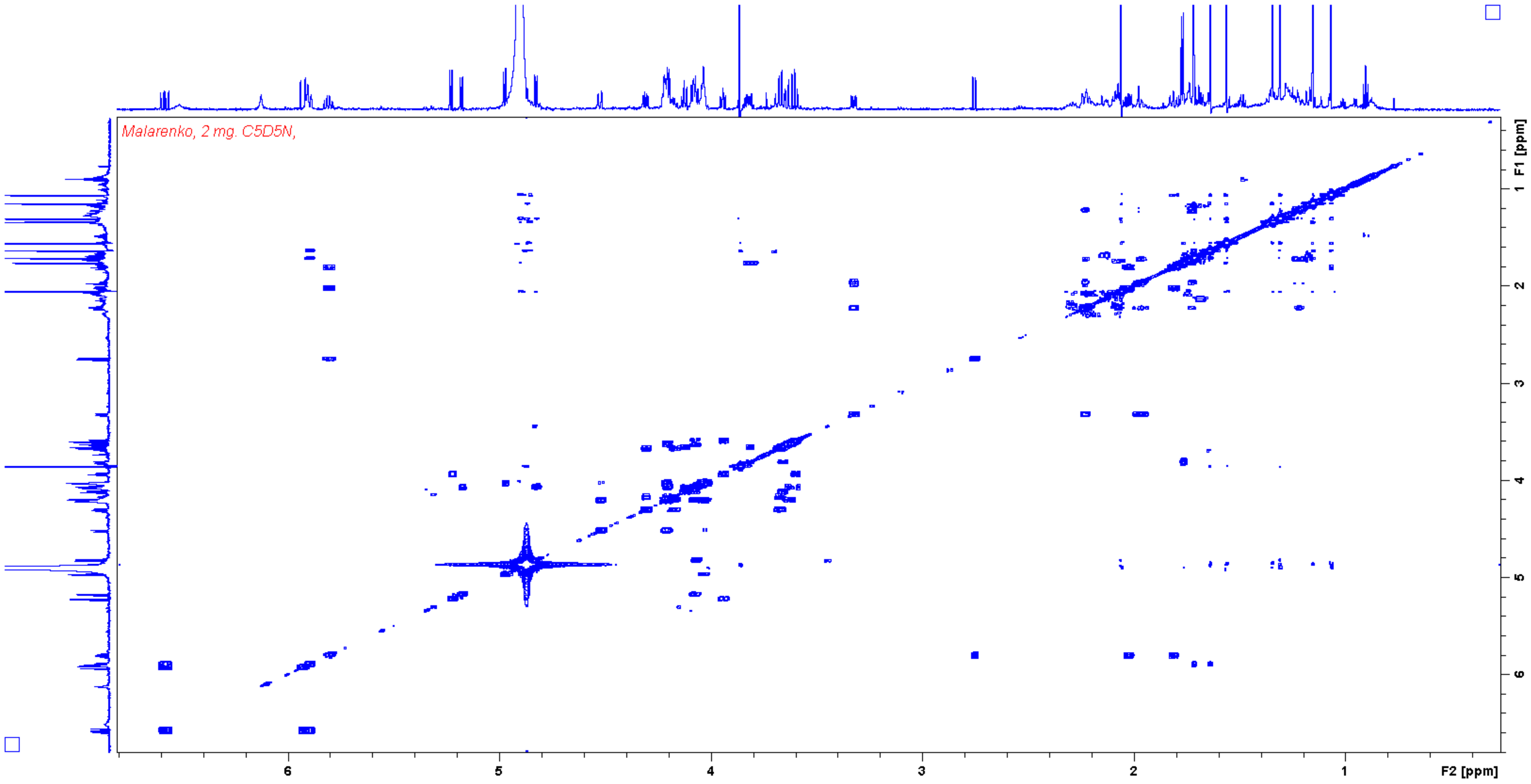

Figure S49. HSQC spectrum of pacificusoside Q (**6**) in C_5D_5N.

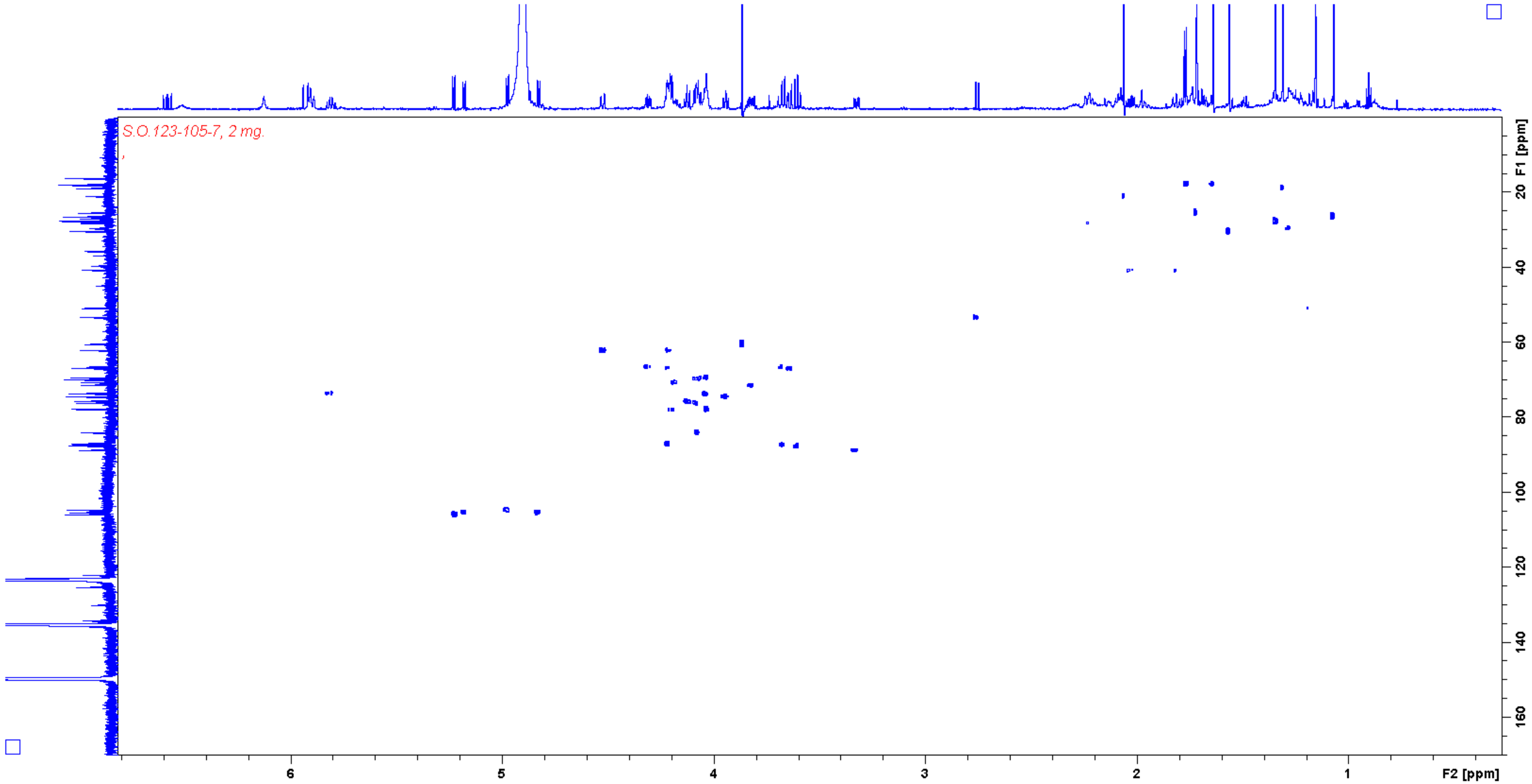

Figure S50. HMBC spectrum of pacificusoside Q (**6**) in C_5D_5N.

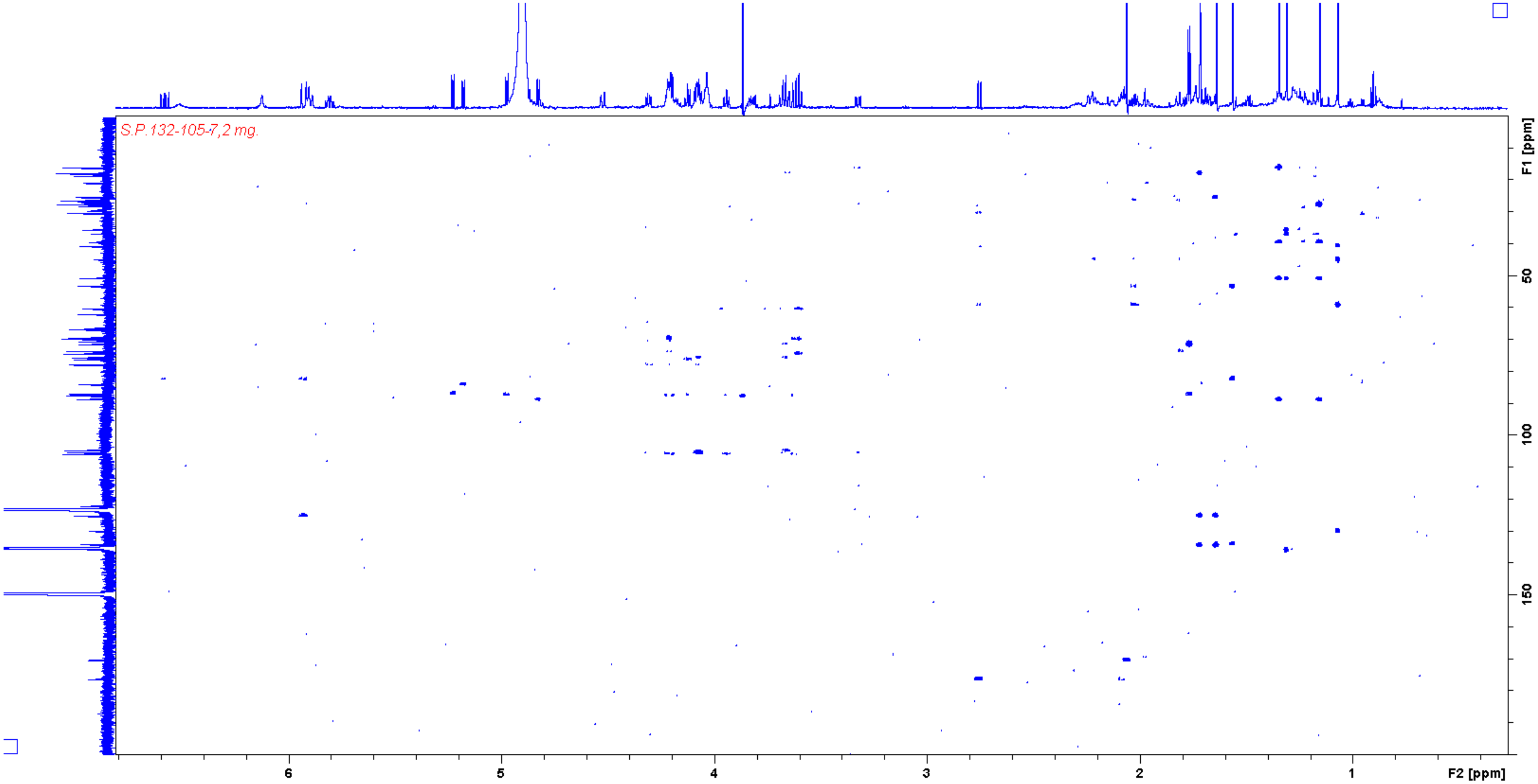

Figure S51. ROESY spectrum of pacificusoside Q (**6**) in C_5D_5N.

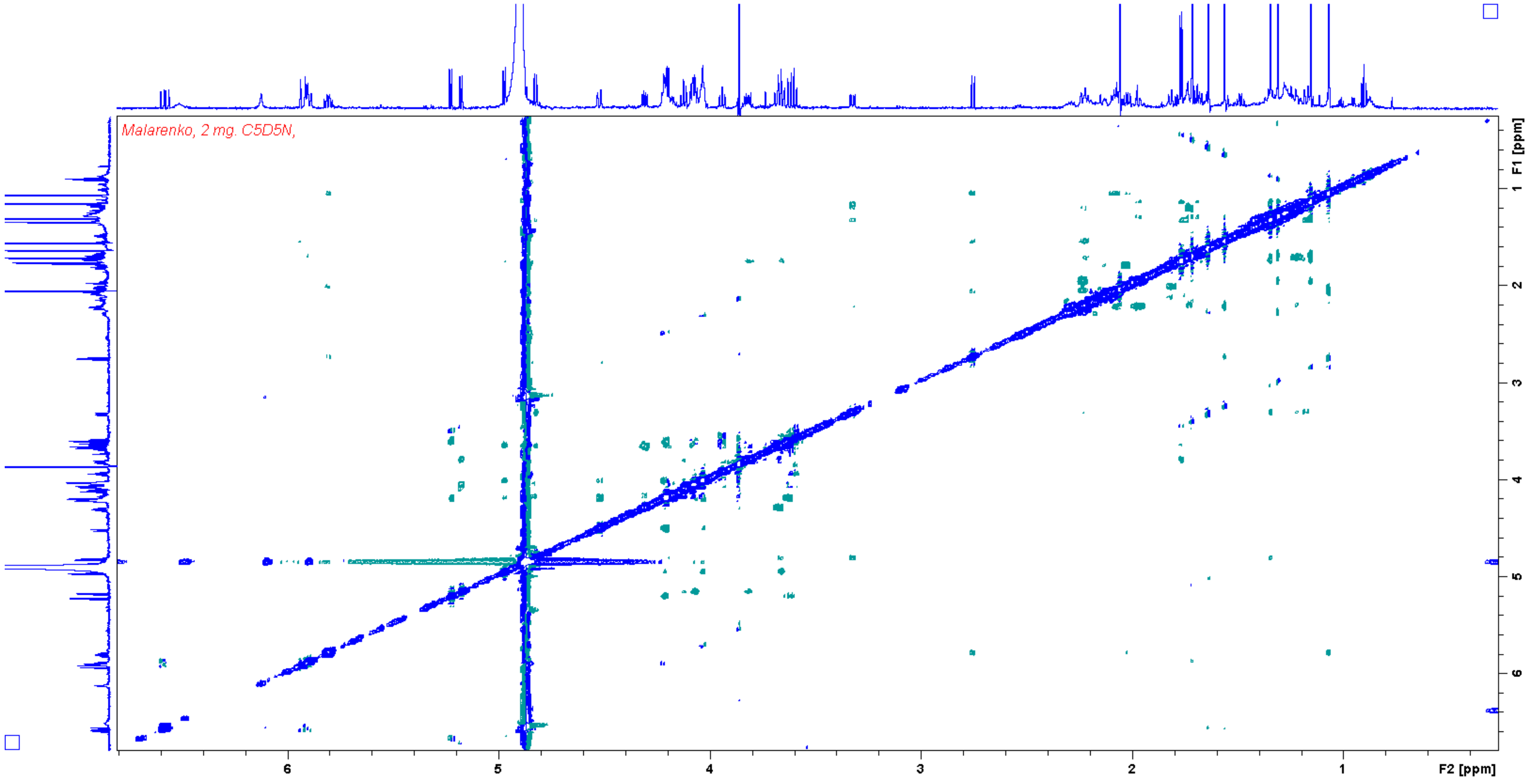

Figure S52. (–)ESIMS/MS spectrum of pacificusoside Q (**6**).

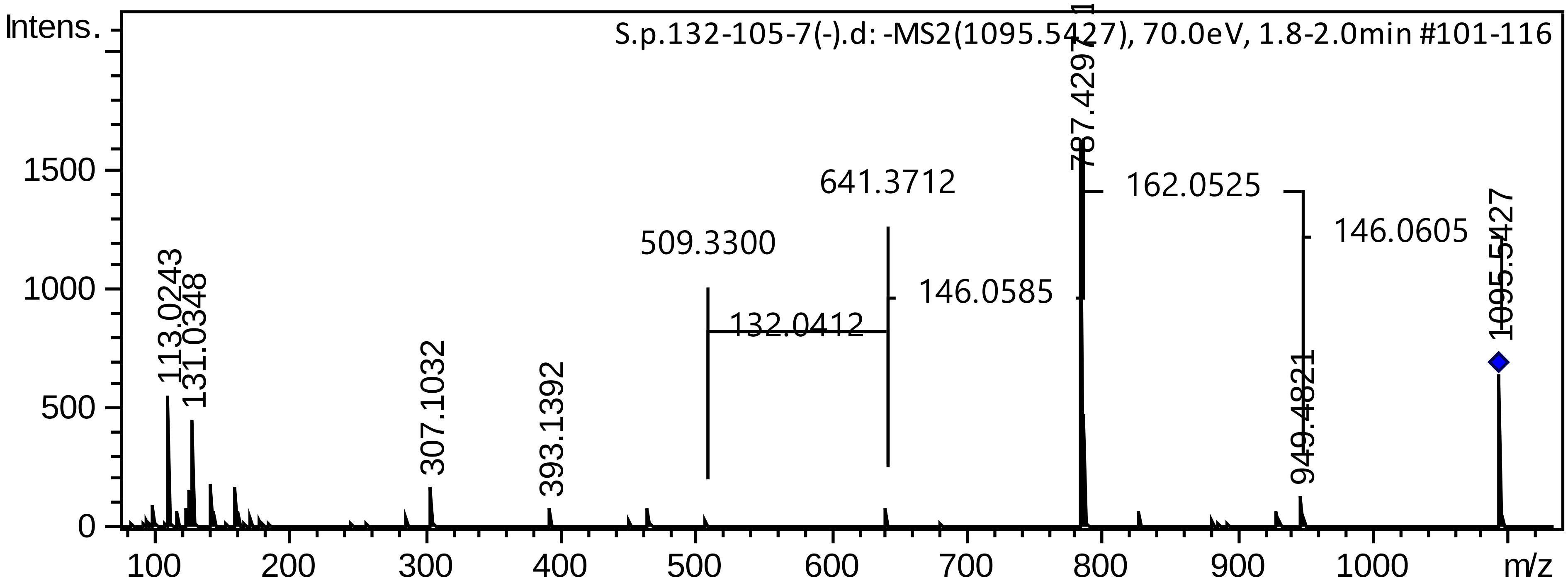

Figure S53. (+)ESIMS/MS spectrum of pacificusoside Q (**6**).

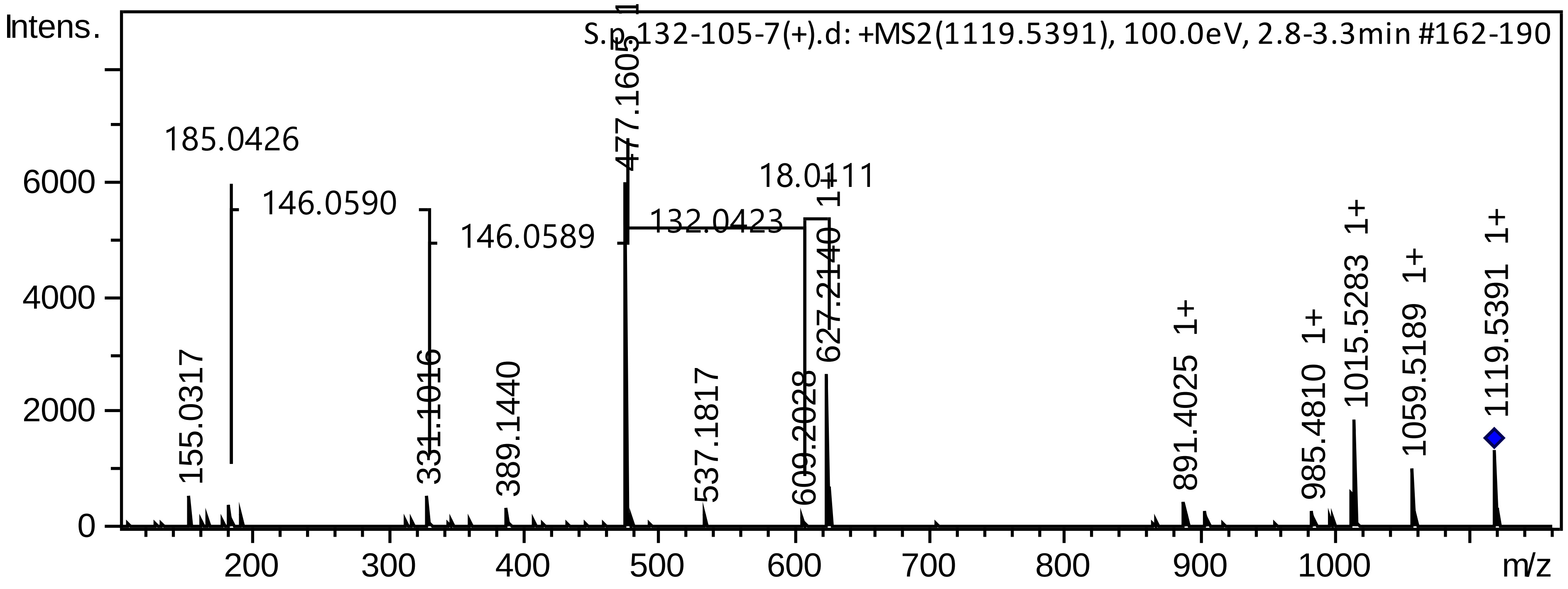

Supplement: Supplementary file 1 [file marinedrugs-22-00019-s001.zip › marinedrugs-2786862-Supplementary material_Malyarenko_MD_2023_clean.pdf]
